# Inequalities in early childhood care and development in low/middle-income countries: 2010–2018

**DOI:** 10.1136/bmjgh-2020-002314

**Published:** 2020-02-04

**Authors:** Chunling Lu, Jorge Cuartas, Günther Fink, Dana McCoy, Kai Liu, Zhihui Li, Bernadette Daelmans, Linda Richter

**Affiliations:** 1 Division of Global Health Equity, Brigham and Women's Hospital/Harvard Medical School, Boston, Massachusetts, USA; 2 Harvard Graduate School of Education, Harvard University, Cambridge, Massachusetts, USA; 3 Instituto Colombiano de Bienestar Familiar (ICBF), Bogotá, Colombia; 4 Department of Epidemiology & Public Health, Swiss Tropical and Public Health Institute, University of Basel, Basel, Switzerland; 5 Department of Social Security, School of Labor and Human Resources, Renmin University of China, Beijing, China; 6 Department of Global Health and Population, Harvard University T.H. Chan School of Public Health, Boston, Massachusetts, USA; 7 Department of Child and Adolescent Health and Development, World Health Organization, Geneva, Switzerland; 8 Centre of Excellence in Human Development, University of the Witwatersrand, Johannesburg-Braamfontein, South Africa

**Keywords:** early childhood development, inequality, children at risk of poor development, home stimulation, early learning, early care and education, Early Childhood Development Index (ECDI)

## Abstract

**Background:**

Inequalities in early childhood development (ECD) tend to persist into adulthood and amplify across the life course. To date, little research on inequalities in early childhood care and development in low/middle-income countries has been available to guide governments, donors and civil society in identifying which young children and families should be targeted by policies and programmes to improve nurturing care that could prevent them from being left behind.

**Methods:**

Using data from 135 Demographic and Health Surveys and Multiple Indicator Cluster Surveys between 2010 and 2018, we assessed levels and trends of inequalities in exposure to risks of stunting or extreme poverty (under age 5; levels in 85 and trends in 40 countries), early attendance of early care and education programmes (36–59 months; 65 and 17 countries), home stimulation (36–59 months; 62 and 14 countries) and child development according to the Early Childhood Development Index (36–59 months; 60 and 13 countries). Inequalities within countries were measured as the absolute gap in three domains—child gender, household wealth and residential area—and compared across regions and country income groups.

**Results:**

63% of children were not exposed to stunting or extreme poverty; 39% of 3–4-year olds attended early care and education; and 69% received a level of reported home stimulation defined as adequate. Sub-Saharan Africa had the lowest proportion of children not exposed to stunting or extreme poverty (45%), attending early care and education (24%) and receiving adequate home stimulation (47%). Substantial gaps in all indicators were found across country income groups, residential areas and household wealth categories. There were no significant reductions in gaps over time for a subset of countries with available data in two survey rounds.

**Conclusions:**

Available data indicate large inequalities in early experiences and outcomes. Efforts of reducing these inequalities must focus on the poorest families and those living in rural areas in the poorest countries. Improving and applying population-level measurements on ECD in more countries over time are important for ensuring equal opportunities for young children globally.

**Key questions**

**What is already known?**

- Some 249 million (or 43% of) children under age 5 in low/middle-income countries (LMICs) were at risk of poor development in 2010 due to exposure to stunting or extreme poverty, mostly concentrated in South Asia and sub-Saharan Africa.
- Large disparities by residential area and wealth quintile in risk exposure, home simulation, early care and education, and early development have also been documented in a subset of countries.

**What are the new findings?**

- The latest available data suggest that 63% of children were not exposed to stunting or extreme poverty, 39% of 3–4-year olds attended early care and education outside of the home and 69% received levels of home stimulation defined as adequate. Children in sub-Saharan Africa were most disadvantaged on all indicators.
- In most countries, children in urban areas or in the richest household wealth quintiles were doing better, on average, on all indicators than those in rural areas or the lowest wealth quintile. Gaps between boys and girls were negligible.
- In most of the subset of countries with available trend data, there was no reduction in disparities on the four indicators by residential area and household wealth status over time.

**What do the new findings imply?**

- More efforts are needed to reduce inequalities in exposure to poverty, stunting, home stimulation and early education in order to improve early childhood development (ECD).
- More investments are needed to improve and expand measures relevant to ECD in LMICs.

## Introduction

Scientific findings from multiple disciplines demonstrate that early childhood development (ECD) provides a critical foundation for lifetime education, work productivity, physical and mental health, and social well-being.^1–5^ Inequalities in ECD tend to persist across the life course and amplify into adulthood.^6^ Investing in ECD is considered one of the most cost-effective ways to promote human development and to narrow socioeconomic inequalities,^7 8^ and is recognised in the sustainable development goals (SDGs). Target 4.2 of the SDGs aims to ‘By 2030, ensure that all girls and boys have access to quality early childhood development, care and pre-primary education so that they are ready for primary education’.^9^ Complementing this effort, the Development Working Group of the G20 launched an initiative for ECD in 2018.^10^

According to the most recent estimates published in a 2017 Lancet Series,^11^ around 249 million (43% of) children under age 5 in low/middle-income countries (LMICs) were at risk of poor development in 2010 due to stunting or exposure to extreme poverty, with the highest proportion of affected children in South Asia and sub-Saharan Africa. Furthermore, estimates using Early Childhood Development Index (ECDI) data from Multiple Indicator Cluster Surveys (MICS) suggest that more than one third of 3–4-year olds living in LMICs were not on track in their cognitive and social-emotional development.^3 4^ A recent study covering 14 LMICs found that risk of poor development was much higher among rural children than their urban counterparts.^11^ Additional work has shown that rural and poor children had much lower access to learning opportunities in and out of the home, and were less likely to be developmentally on track according to the ECDI relative to their urban and wealthier peers.^3 4 12 13^

The SDGs provide a unique opportunity to ensure that all young children achieve their developmental potential, and to ensure that no child is left behind. Meeting the SDG goal on ECD requires ensuring equal access to high-quality services that promote nurturing care. To date, relatively little systematic evidence has been available to guide governments, donors and civil society in identifying which young children and families should be targeted to prevent them from being left behind. ECD is a newly added component in The Countdown to 2030 for Women’s, Children’s and Adolescents’ Health. Our study forms part of a series on ‘Leaving no woman, no child, and no adolescent behind’ providing a descriptive analysis on progress in improving ECD since 2010.

Using data from 135 Demographic and Health Surveys (DHS)^14^ and MICS^15^ from 94 LMICs, we provide an assessment of current levels and trends of inequalities using available data in four domains of ECD: exposure to stunting and/or extreme poverty as risk factors for poor development, attendance of early care and education (ECE) programme, stimulation at home and caregiver-reported levels of early development using the ECDI. These data provide unique information to monitor progress towards achieving the ECD target set out in the SDGs.

## Methods

### Conceptual framework

Our analysis is structured according to the Nurturing Care Framework originally proposed in the 2016 Lancet Series Advancing Early Childhood Development: from science to scale^3–5^ and formally adopted by the WHO, UNICEF and the World Bank in 2018.^16^ The Framework emphasises ‘a stable environment created by parents and other caregivers that ensures children's good health and nutrition, protects them from threats, and gives young children opportunities for early learning, through interactions that are emotionally supportive and responsive’.^16^ Nurturing care includes an enabling policy environment and five core components essential for early development: health, nutrition, early learning, responsive caring, and safety and security.

### Empirical approach

We use all available data from 2010 to 2018 to assess levels and trends of inequalities in the (1) prevalence of children not exposed to stunting or extreme poverty, (2) percentage of children attending early childhood care and education outside of the home, (3) percentage of children experiencing adequate home stimulation in response to questions asked in the MICS and (4) percentage of children developmentally on track according to the ECDI (see box 1 for the skills assessed by the ECDI). Young children’s access to healthcare is analysed as part of another study in this collection.^17^ As illustrated in online supplementary appendix chapter 1 table 1, our analyses explore inequality at two levels. Within countries, we assessed the levels and trends of inequalities of the four ECD indicators by child gender, residential area and household wealth quintile. At the aggregate level, we assessed the average-level inequalities by gender, area and wealth across regions and country income groups.

10.1136/bmjgh-2020-002314.supp1 Supplementary data

Box 1 **Skills assessed by the Early Childhood Development Index**

**Literacy-numeracy**

- Can (name) identify or name at least ten letters of the alphabet?
- Can (name) read at least four simple, popular words?
- Does (name) know the name and recognise the symbol of all numbers from 1 to 10?

**Physical**

- Can (name) pick up a small object with two fingers, like a stick or a rock from the ground?
- Is (name) sometimes too sick to play? (*reverse coded*)

**Approaches to learning**

- Does (name) follow simple directions on how to do something correctly?
- When given something to do, is (name) able to do it independently?

**Social-emotional**

- Does (name) get along well with other children?
- Does (name) kick, bite or hit other children or adults? (*reverse coded*)
- Does (name) get distracted easily? (*reverse coded*)

Source: http://mics.unicef.org.

### Outcome measures

We analysed four indicators of importance to ECD:

1. Prevalence of children under 5 years not at risk of poor development due to stunting or living in extreme poverty. These two risk factors are well-defined, and strongly associated with both childhood and adulthood health and human capital outcomes.^11 18–21^ They have been used in previous studies to measure children at risk of poor development.^11 22^Data on stunting were available in both the DHS and MICS and were defined by WHO growth standards as height-for-age z-score two or more SD below the international reference median.^23^ Neither the DHS nor the MICS collect direct information on an individual’s status of living in extreme poverty. Following previous studies,^11 19^ we used two alternative variables (household wealth index and household weights) together with a country’s rate of extreme poverty (less than $1.9 per person per day at 2011 intecrnational prices) obtained from the World Bank^24^ to generate a dichotomous variable indicating whether a young child lived in extreme poverty. Details are provided in online supplementary appendix chapter 2.
2. Percentage of young children (36–59 months) attending ECE programme outside of the home. Both the MICS and the DHS ask caregivers of 3–4-year olds whether their children have ever attended any form of early childhood care or pre-primary education.
3. Percentage of young children (36–59 months) receiving home stimulation. The home stimulation module in MICS and DHS surveys asks caregivers to report whether an adult engaged with the child in the following six basic activities at home in the 3 days preceding the survey: reading books or looking at pictures; telling stories; singing songs; taking the child outside; playing with the child; and naming, counting or drawing with the child. Following previous work using these data,^12 25^ we totalled the number of activities that adults engaged in with the child, and defined adequate home stimulation as exposure to at least four out of six activities.
4. Percentage of young children (36–59 months) developmentally on track according to the ECDI. The ECDI is a 10-item scale developed by UNICEF that has been included in the MICS and selected DHS since 2009 to assess basic developmental milestones of 3–4-year olds. The ECDI relies on caregiver reports and includes items from four domains of early development: literacy-numeracy, physical, approaches to learning and social-emotional (see box 1).^26^ Following UNICEF guidelines,^26^ we considered a child to be ‘developmentally on track’ if the child was on track in at least three out of the four ECDI domains. Within each of the four ECDI domains, a child was considered to be on track if the caregiver reported the child to have at least 50% of the relevant skills. Although limited in scope, depth and psychometric evidence, the ECDI is the first available tool for capturing population-level development. It is currently adopted as an interim measure for reporting on goal 4.2.1 in the Secretary-General’s Annual SDG Progress Reports.^27^

### Data sources

We used nationally representative household surveys since 2010 for countries with available DHS and MICS data. These comparable surveys use similar sampling design and both collect comprehensive information about the health, nutrition, and well-being of women and children. To measure progress over time, we divided the surveys into two rounds: round one (baseline, surveys between 2010 and 2012 for exposure to stunting or extreme poverty, surveys in 2010 for the other three indicators), and round two (surveys since 2013 for exposure to stunting or extreme poverty, surveys since 2011 for other three indicators). For countries with multiple surveys in each round, we used the most recent.

A total of 135 MICS and DHS surveys administered in 94 countries between 2010 and 2018 were analysed. Data on risk exposure were available in 85 countries (30 low-income, 30 lower-middle income and 25 upper-middle income); data on ECE in 65 countries (19 low-income, 22 lower-middle income and 24 upper-middle income); data on home stimulation in 62 countries (17 low-income, 21 lower-middle income and 24 upper-middle income); and data on ECDI in 60 countries (17 low-income, 20 lower-middle income and 23 upper-middle income). The median survey year was 2014, with an IQR between 2012 and 2015 (online supplementary appendix chapter 1 and table 2).

### Statistical analysis

To quantify inequalities between subgroups, we computed absolute differences between population groups for each of the four ECD indicators. Within countries, we compared the difference in each indicator by child gender (boy/girl), residential area (rural/urban) and household wealth quintiles (poorest/richest). Wealth quintiles are included in both the MICS and the DHS surveys and are based on household asset ownership following the methodology outlined previously.^28^ We did not measure inequality by wealth quintiles for risk exposure, as the indicator includes child poverty status. At the aggregate level, we compared the average level of inequalities in the four indicators across country income groups and regions. Information on measuring within-country and aggregate-level mean values and 95% CIs of absolute inequalities by gender, area and wealth is presented in online supplementary appendix chapter 3.

We used data from the most recent surveys to generate up-to-date percentage or coverage estimates for the four indicators and their inequalities. For countries with data in both rounds (40 for risk exposure, 17 countries on ECE and 14 for home stimulation and ECDI), we tracked changes in the level of estimates and their inequalities over time. Information on the measure of country-level mean values and 95% CIs for each indicator, and their absolute inequalities by different dimensions is presented in online supplementary appendix chapter 3. We used Stata V.14.2 for all analyses.

### Patient and public involvement

Patients were not involved in this study.

## Results

### Aggregate-level estimates by region and country income group in the latest year

On average, 63.2% of children in 85 countries were neither exposed to stunting nor to extreme poverty, with the highest proportion found in Europe and Central Asia (88.3%) and the lowest in sub-Saharan Africa (45.2%) (table 1). Across 65 countries with available data, fewer than two fifths (38.9%) of children ever attended an ECE programme, with the highest level in East Asia and the Pacific (67.4%) and lowest in sub-Saharan Africa (24.1%). Just over two thirds (69.1%) of children were exposed to home stimulation defined as adequate, with the highest percentage in Europe and Central Asia (90.1%) and lowest in sub-Saharan Africa (46.9%). In the 60 countries with ECDI data, 75.1% of children were rated as developmentally on track according to the ECDI cut-offs, with the lowest proportion in sub-Saharan Africa (60.7%).

**Table 1:** Aggregate-level estimates and 95% CIs of the four indicators

	Not exposed to stunting or extreme poverty (%)	ECE (%)*	Home stimulation (%)	ECDI (%)†
	85 countries	65 countries	62 countries	60 countries
Average	63.2 (58.1 to 68.2)	38.9 (36.4 to 41.4)	69.1 (66.8 to 71.4)	75.1 (73.0 to 77.3)
Region				
East Asia and Pacific	65.8 (51.0 to 80.5)	67.4 (65.3 to 69.5)	74.9 (72.7 to 77.2)	79.2 (77.1 to 81.3)
Europe and Central Asia	88.3 (85.4 to 91.2)	43.4 (39.9 to 46.9)	90.1 (88.2 to 92.0)	91.0 (88.9 to 93.0)
Latin America and the Caribbean	81.0 (73.5 to 88.5)	52.8 (49.4 to 56.2)	78.6 (75.7 to 81.4)	84.9 (82.2 to 87.6)
Middle East and North Africa	82.3 (71.2 to 93.4)	42.4 (39.4 to 45.4)	78 (75.0 to 81.0)	75.9 (73.8 to 78.0)
South Asia	62.9 (54.7 to 71.1)	30.7 (29.0 to 32.3)	74.5 (72.9 to 76.2)	74.1 (72.1 to 76.1)
Sub-Saharan Africa	45.2 (39.5 to 50.8)	24.1 (22.5 to 25.8)	46.9 (44.8 to 49.1)	60.7 (58.8 to 62.6)
Country income class				
Low-income	44.7 (37.8 to 51.7)	21.5 (20.1 to 22.8)	54.3 (52.4 to 56.2)	60.1 (58.4 to 61.8)
Lower-middle income	64.4 (57.2 to 71.7)	37.6 (35.3 to 39.9)	61.8 (59.4 to 64.1)	73.6 (71.3 to 75.9)
Upper-middle income	83.8 (79.3 to 88.4)	53.1 (49.6 to 56.6)	85.3 (82.8 to 87.8)	86.9 (84.5 to 89.3)

*Early care and education programmes.

†Early Childhood Development Index.

ECDI, Early Childhood Development Index; ECE, early care and education.

Compared with children in lower income countries, children in higher income countries had higher proportions of children not exposed to stunting or extreme poverty, and higher levels of ECE participation, adequate home stimulation and ECDI scores.

### Aggregate-level inequalities by child gender, residential area and household wealth in the latest year

Table 2 reports the gender gap on the four indicators. On average, the differences between boys and girls were either small (risk exposure 1.7 percentage points (pp) with 95% CI 1.3 pp and 2.2 pp) or not statistically different from zero (eg, ECE −0.6 pp with 95% CI −5.3 pp and 4.2 pp). Girls were less likely to be exposed to stunting or extreme poverty than boys in Latin America and the Caribbean (−1.6 pp with 95% CI −2.2 pp and −1.0 pp), sub-Saharan Africa (−2.5 pp with 95% CI −3.2 pp and −1.9 pp), and in higher income groups relative to low-income groups. For the ECDI, we found a female advantage in the Middle East and North Africa (−6.2 pp with 95% CI −10.4 pp and −2.0 pp), South Asia (−4.6 pp with 95% CI −8.5 pp and −0.7 pp) and sub-Saharan Africa (−4.7 pp with 95% CI −8.5 pp and −0.9 pp).

**Table 2:** Aggregate-level disparity by gender (boy–girl)* and 95% CIs of the four indicators

	Not exposed to stunting or extreme poverty (%)	ECE (%)†	Home stimulation (%)	ECDI (%)‡
	85 countries	65 countries	62 countries	60 countries
Average	−1.7 (−2.2 to −1.3)	−0.6 (−5.3 to 4.2)	−0.6 (−4.8 to 3.6)	−4.0 (−8.3 to 0.3)
Region				
East Asia and Pacific	−1.0 (−2.1 to 0.2)	0.8 (−3.2 to 4.7)	0.8 (−3.2 to 4.7)	−3.1 (−7.4 to 1.1)
Europe and Central Asia	−0.6 (−1.6 to 0.4)	0.6 (−6.0 to 7.2)	−1.0 (−4.8 to 2.7)	−2.2 (−6.2 to 1.8)
Latin America and the Caribbean	−1.6 (−2.2 to −1.0)	−0.3 (−6.9 to 6.3)	−1.2 (−6.9 to 4.5)	−3.7 (−9.1 to 1.7)
Middle East and North Africa	−1.3 (−3.2 to 0.5)	−0.3 (−6.2 to 5.6)	−1.1 (−5.2 to 2.9)	−6.2 (−10.4 to −2.0)
South Asia	−0.8 (−1.6 to 0.1)	0.1 (−3.2 to 3.3)	0.2 (−3.1 to 3.4)	−4.6 (−8.5 to −0.7)
Sub-Saharan Africa	−2.5 (−3.2 to −1.9)	−1.8 (−4.9 to 1.3)	−0.3 (−4.1 to 3.6)	−4.7 (−8.5 to −0.9)
Country income class				
Low-income	−1.5 (−2.2 to −0.8)	−1.0 (−3.5 to 1.6)	0.8 (−2.4 to 4.1)	−5.2 (−8.6 to −1.8)
Lower-middle income	−2.1 (−2.7 to −1.4)	−0.3 (−4.6 to 4.1)	−1.3 (−5.9 to 3.2)	−4.0 (−8.5 to 0.5)
Upper-middle income	−1.6 (−2.5, −0.7)	−0.6 (−7.4 to 6.2)	−0.8 (−5.4 to 3.7)	−3.3 (−7.9 to 1.4)

*Gender gaps are defined as the difference between boy and girl averages. A negative gender gap implies a girl advantage, while a positive gender gap a boy advantage.

†Early care and education programmes.

‡Early Childhood Development Index.

ECDI, Early Childhood Development Index; ECE, early care and education.

Rural–urban differences were observed in almost all regions and incomes, favouring children in urban areas. Specifically, there was an average gap (favouring urban children) of 22.6 pp in the proportion of children not exposed to stunting or extreme poverty, with the largest gap in sub-Saharan Africa (37.2 pp). This rural–urban gap averaged 15.6 pp for ECE participation, with the largest rural–urban gap in Europe and Central Asia (22.2 pp). There was an average rural-urban gap of 9.8 pp for home stimulation, with the largest gap in East Asia and the Pacific (12.6 pp). Finally, there was an average rural-urban gap of 5.7 pp for the proportion of children on track according to the ECDI, with the largest gap in South Asia (10.1 pp). Upper-middle income countries had lower average rural–urban gaps than low-income or lower-middle income countries (table 3).

**Table 3:** Aggregate-level disparity by residential area* and 95% CIs of the four indicators

	Not exposed to stunting or extreme poverty (%)	ECE (%)†	Home stimulation (%)	ECDI (%)‡
	85 countries	65 countries	62 countries	60 countries
Average	−22.6 (−26.5 to −18.7)	−15.6 (−21.1 to −10.1)	−9.8 (−14.3 to −5.3)	−5.7 (−10.4 to −1.0)
Region				
East Asia and Pacific	−18.8 (−27.5 to −10.2)	−5.6 (−9.8 to −1.4)	−12.6 (−16.5 to −8.7)	−6.8 (−11.3 to −2.3)
Europe and Central Asia	−3.8 (−7.1 to −0.5)	−22.2 (−28.6 to −15.8)	−4.2 (−8.1 to −0.3)	−1.1 (−5.2 to 3.1)
Latin America and the Caribbean	−13.7 (−20.1 to −7.3)	−11.8 (−18.5 to −5.0)	−9.7 (−15.7 to −3.7)	−4.2 (−10.4 to 2.0)
Middle East and North Africa	−5.5 (−12.5 to 1.5)	−11.4 (−21.1 to −1.8)	−8.1 (−12.3 to −3.9)	−2.0 (−6.3 to 2.4)
South Asia	−11.8 (−14.6 to −9.0)	−11.0 (−15.1 to −6.9)	−10.2 (−13.8 to −6.6)	−10.1 (−14.6 to −5.7)
Sub-Saharan Africa	−37.2 (−41.6 to −32.8)	−18.6 (−22.4 to −14.7)	−12.4 (−16.8 to −8.0)	−8.7 (−13.1 to −4.4)
Country income class				
Low-income	−34.1 (−40.3 to −28.0)	−15.6 (−19.0 to −12.1)	−10.4 (−14.3 to −6.5)	−8.1 (−12.1 to −4.0)
Lower-middle income	−21.7 (−27.4 to −16.0)	−16.3 (−20.9 to −11.7)	−12.2 (−17.0 to −7.4)	−6.8 (−11.5 to −2.0)
Upper-middle income	−9.8 (−14.9 to −4.7)	−14.9 (−22.8 to −7.1)	−7.1 (−11.8 to −2.4)	−3.1 (−8.2 to 2.1)

*Gaps by residential area are defined as the difference between rural and urban averages. A negative area gap implies urban advantage, while a positive area gap rural advantage.

†Early care and education programme.

‡Early Childhood Development Index.

ECDI, Early Childhood Development Index; ECE, early childhood and education.

For attendance in an ECE programme, on average, the gap between households in the richest quintile and those in the poorest quintile was 29.7 pp, with the largest gap in Europe and Central Asia (39.9 pp), and the smallest in South Asia (16 pp). Regarding home stimulation, on average, the gap between the richest and poorest quintiles was 21.7 pp and was highest in East Asia and Pacific (28.5 pp) and Latin America and the Caribbean (28.5 pp), and lowest in Europe and Central Asia (11.4 pp). Finally, children in the richest quintile of household wealth were more likely to be developmentally on track according to the ECDI than those in the poorest quintile, with an average gap of 12 pp. Household wealth inequalities in the ECDI were lowest in Europe and Central Asia (3.4 pp), and highest in South Asia (16.8 pp) (table 4).

**Table 4:** Aggregate-level disparity by wealth quintiles* and 95% CIs of the three indicators

	ECE (%)†	Home stimulation (%)	ECDI (%)‡
	65 countries	62 countries	60 countries
Average	29.7 (22.1 to 37.3)	21.7 (15.6 to 27.9)	12.0 (5.7 to 18.3)
Region			
East Asia and Pacific	19.9 (14.2 to 25.6)	28.5 (22.5 to 34.5)	13.7 (7.1 to 20.4)
Europe and Central Asia	39.9 (31.2 to 48.6)	11.4 (5.5 to 17.3)	3.4 (−2.1 to 9.0)
Latin America and the Caribbean	30.5 (21.4 to 39.5)	28.5 (20.2 to 36.9)	16.0 (8.1 to 23.9)
Middle East and North Africa	20.4 (5.8 to 35.0)	19.7 (13.9 to 25.4)	11.0 (5.1 to 17.0)
South Asia	16.7 (12.0 to 21.4)	22.1 (17.7 to 26.6)	16.8 (11.0 to 22.7)
Sub-Saharan Africa	31.4 (25.8 to 37.1)	22.3 (16.6 to 28.0)	13.0 (7.4 to 18.7)
Country income class			
Low-income	23.9 (18.6 to 29.3)	20.6 (15.6 to 25.6)	13.2 (8.2 to 18.2)
Lower-middle income	33.7 (27.4 to 40.0)	26.5 (19.8 to 33.2)	12.5 (5.7 to 19.4)
Upper-middle income	30.8 (20.3 to 41.2)	18.2 (11.7 to 24.7)	10.6 (3.9 to 17.4)

*Gaps by wealth quintile are defined as the difference between the richest and poorest quintile averages. A negative area gap implies poorest advantage, while a positive gap richest advantage.

†Early care and education programmes.

‡Early Childhood Development Index.

ECDI, Early Childhood Development Index; ECE, early care and education.

### Country-level prevalence and inequalities in the latest year

#### Children not exposed to the risks of stunting or extreme poverty

**Figure 1:**
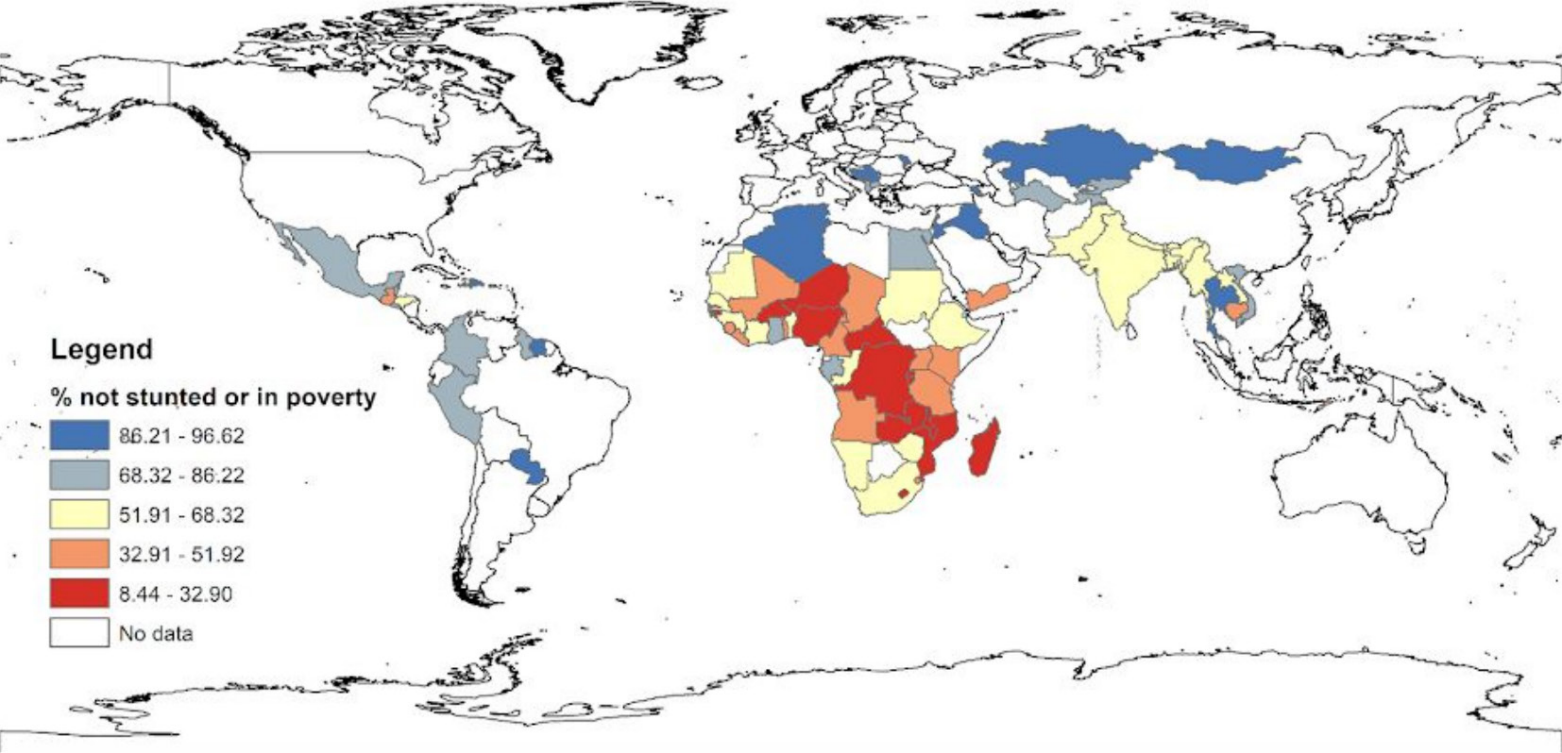
Proportion of children not exposed to stunting or extreme poverty.

Among the 85 countries included in the analysis, the highest proportions of children not exposed to stunting or extreme poverty were found in St Lucia (96.6%), Serbia (93.9%) and Moldova (93.6%), and the lowest in Mozambique (14.2%), Burundi (14.5%) and Malawi (15.8%), all of which were in sub-Saharan Africa (figure 1, online supplementary appendix chapter 4 table 1). The gender gap favoured girls in all countries but Iraq, and was statistically significant (p<0.05) in 26 countries (ranging from 0.7 pp in India to 7.4 pp in Swaziland), mostly in sub-Saharan Africa. The rural–urban gap favoured urban children and was statistically significant (p<0.05) in 68 countries, ranging from 4.7 pp in Moldova to 63 pp in Togo (online supplementary appendix chapter 4 table 1 and 2 for details).

#### ECE participation

**Figure 2:**
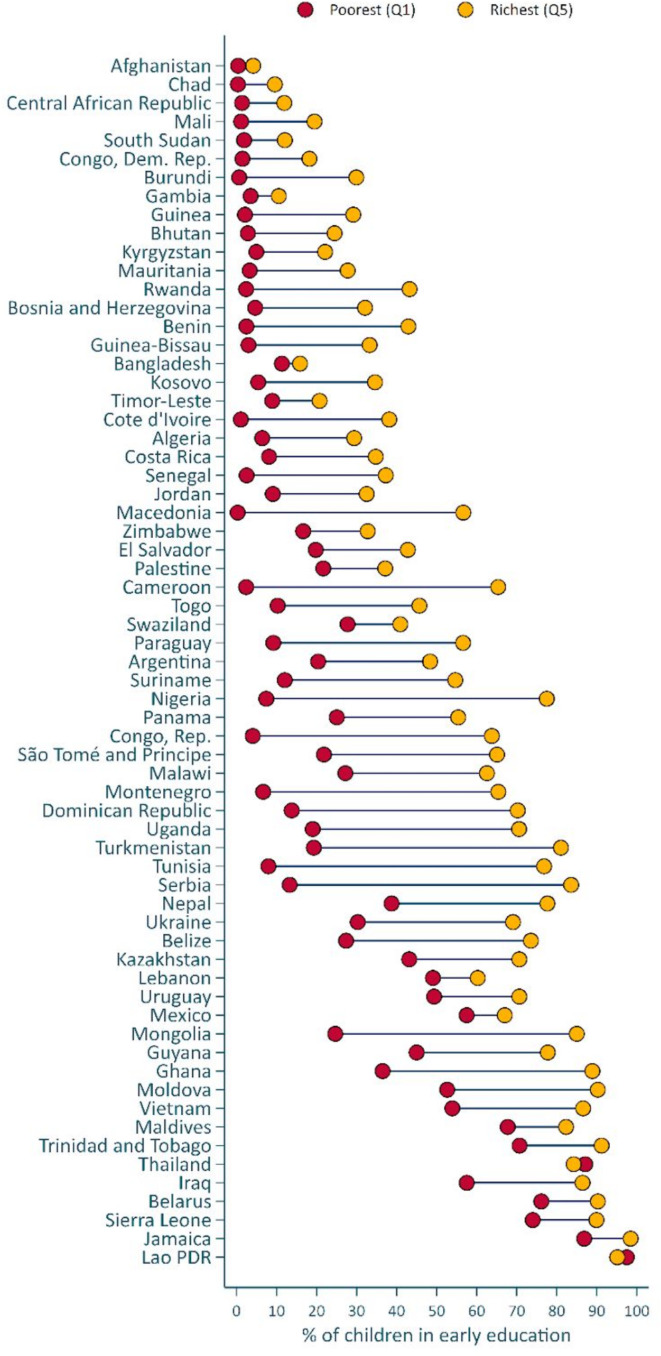
Proportion of children attending early care and education by wealth quintile.

Of the 65 countries with data, the lowest ECE participation was in Afghanistan (1%), Chad (2%) and Central African Republic (4%). ECE participation was highest in Lao PDR (95%), Jamaica (92%) and Sierra Leone (89%). Gender gaps favoured girls and were statistically significant (p value <0.05) in five countries, ranging from 4.1 pp in Malawi to 7.5 pp in Swaziland (online supplementary appendix chapter 4 table 6). The rural–urban gap (ranging from 2.8 pp in Gambia to 42.9 pp in Tunisia) was statistically significant in 52 countries, mostly favouring urban children with the exception of Lebanon (10.2 pp) and Thailand (5.9 pp), where the gap favoured children in rural areas (online supplementary appendix chapter 4 table 7). Figure 2 shows the household wealth gaps among children attending ECE (See online supplementary appendix chapter 4 table 8 for details). Household wealth gaps were observed (p<0.05) in 59 countries, favouring children in the richest quintile. The countries with the smallest wealth gaps were Afghanistan (3.7 pp), Bangladesh (4.5 pp) and Gambia (7.0 pp), whereas the largest gaps were observed in Serbia (70.3 pp), Nigeria (70.1 pp) and Tunisia (68.9 pp).

#### Home stimulation

Among 62 countries with data on home stimulation, the lowest levels were reported in sub-Saharan African, in particular in Senegal (27.9%), Benin (28.1%) and Cote d’Ivoire (28.9%), and the highest in Europe and Central Asia, in particular in Ukraine (99.2%), Montenegro (98.7%) and Maldives (97.7%). Gender gaps, favouring girls, were statistically significant (p value <0.05) in 10 countries (ranging from 3.9 pp in Ghana to 12.8 pp in Guinea-Bissau) (online supplementary appendix chapter 4 Table 13). Rural–urban gaps favoured urban children and were statistically significant (p value <0.05) in 45 countries, ranging from 3.2 pp in Turkmenistan to 30.0 pp in Tunisia (online supplementary appendix chapter 4 table 14). Within-country wealth gaps favoured children in the richest quintile and were statistically significant (p value <0.05) in 56 countries, ranging from 2.3 pp in Maldives to 51.5 pp in Sierra Leone (online supplementary appendix chapter 4 table 15).

#### Children developmentally on track according to the ECDI

Of the 60 countries with data, the countries with the largest percentage of children developmentally on track according to the ECDI were Bosnia and Herzegovina (96.96%), Serbia (96.6%) and Montenegro (96.0%), all located in Europe and Central Asia. The three countries with the lowest percentage were Burundi (40%), Central African Republic (49%) and Chad (36%), all located in sub-Saharan Africa. In 29 countries, girls were statistically more likely to be on track developmentally, according to the ECDI, than boys (p value <0.05) (online supplementary appendix chapter 4 table 20). The proportion of children on track was higher in urban than in rural areas in 29 countries, particularly in sub-Saharan Africa (online supplementary appendix chapter 4 tables 21). Household wealth gaps favoured children in the richest quintile and were statistically significant in 49 countries, ranging from 5.0 pp in Chad to 38.2 pp in Nigeria (online supplementary appendix chapter 4 table 22).

### Country-level progress in reducing inequalities

#### Children not exposed to stunting or extreme poverty

For 40 countries with data on stunting and extreme poverty in two survey rounds, 30 experienced a significant increase (p value<0.05) in the proportion of children not exposed to stunting or extreme poverty. Improvements ranged from 2.7 pp in Palestine (95% CI 1.7 to 3.7 pp) to 25.5 pp in Tajikistan (95% CI 22 to 29 pp). Nigeria is the only country that had a decrease in the percentage of children not exposed to stunting or extreme poverty, from 40.6% in 2011 to 30.9% in 2016 (online supplementary appendix chapter 4 table 3).

The boy–girl gap remained statistically unchanged in 37 countries. Mongolia had a reduction in the boy–girl gap. Iraq and Swaziland had an increase in the gap, resulting from lower exposure to these risk factors among girls than boys in Swaziland and more exposure among girls than boys in Iraq in the second round (online supplementary appendix chapter 4 table 4).

The rural–urban gap in children not exposed to stunting or poverty declined in only seven countries, all in the low-income group. Tajikistan had the largest drop in the gap (from 26.1 pp in 2012 to 1.9 pp in 2017). Four countries had an increase in the rural–urban gap—Democratic Republic of the Congo, Malawi, Sierra Leone and Zimbabwe—due to greater improvement in urban areas (online supplementary appendix chapter 4 table 5). For example, in Sierra Leone, the gap was 23.7 pp, with a rural percentage of children exposed to neither stunting nor poverty of 18.2% and an urban prevalence of 41.9% in 2010. In 2017, the percentage increased to 22.9% in rural areas and 78.3% in urban areas, a gap of 55.4 pp.

#### ECE attendance

Online supplementary appendix chapter 4 table 9 presents changes in the proportion of children participating in ECE in 16 countries with two rounds of data. In most countries, there was a statistically detectable increase in the proportion of children participating in ECE, particularly in Lao PDR (72 pp), Iraq (83 pp) and Sierra Leone (75 pp). In contrast, Mauritania (2 pp) and Nigeria (7 pp) experienced reductions in the proportion of children participating in ECE. The gender gap in ECE participation reduced in Lao PDR by 6 pp and remained statistically unchanged in the remaining countries (online supplementary appendix chapter 6 table 10). A statistically significant reduction (p value <0.05) was observed in the urban–rural gap in ECE participation in Lao PDR (39 pp), Chad (5 pp), Mauritania (9 pp) and Nigeria (5 pp). The urban–rural gap in ECE participation increased in Swaziland (14 pp) (online supplementary appendix chapter 4 table 11). The wealth gap in ECE participation changed in six countries, with four experiencing significant reductions (p value <0.05) (Chad (5.4 pp), Kazakhstan (14.2 pp), Lao PDR (58.9 pp), and Mauritania (12.8 pp)), and one experiencing an increase (p value <0.05, Serbia (15.4 pp)) (online supplementary appendix chapter 4 table 12).

#### Home stimulation

Among 13 countries with data on home stimulation in two survey rounds, two countries had a statistically significant increase in children’s exposure to adequate levels of stimulation at home, particularly salient in Palestine (12.4 pp). Eight countries, however, experienced statistical reductions in children’s exposure to adequate levels of stimulation at home, particularly salient in Sierra Leone (25.9 pp) and Lao PDR (13.2 pp). The only country that had an increase in the home stimulation gap between boys and girls was Swaziland (12.4 pp). The area gap in stimulation increased in Thailand (6.1 pp), Mauritania (14.4 pp) and Sierra Leone (8 pp), and reduced in Iraq (9.8 pp). The wealth gap in average levels of stimulation at home remained mostly unchanged in the 14 countries, except in Belize and Iraq, where the gap reduced (by 11.3 and 14.9 pp, respectively), and Mauritania and Vietnam, where the gap increased (26.4 pp and 14.0 pp, respectively). See online supplementary appendix chapter 4 tables 16–19 for details.

#### Children developmentally on track according to the ECDI

Of the 13 countries with two survey rounds with ECDI data, six experienced statistically significant (p value <0.05) changes in the proportion of children scored as on track over time (online supplementary appendix chapter 4 table 23). Democratic Republic of the Congo experienced the largest increase in proportion of children scored developmentally on track on the ECDI (15.8 pp), whereas Belize and Mongolia experienced reductions (3.7 pp and 12.0 pp, respectively). The gender gap in the ECDI increased in Palestine by 6.4 pp (p value <0.05), but remained unchanged in the other countries (online supplementary appendix chapter 4 table 24). Furthermore, the urban–rural gap in the ECDI remained constant in most countries, but reduced considerably in Cameroon and Democratic Republic of the Congo (online supplementary appendix chapter 4 table 25). Finally, there were reductions in the wealth gap in the ECDI in Cameroon, Chad and Swaziland, whereas wealth inequality increased in Nigeria (by 6.8 pp) and Thailand (5.9 pp).

## Discussion

We used data from 135 national household surveys collected since 2010 from 94 countries, to provide the most comprehensive report to date of four indicators of early childhood experiences and outcomes. At the aggregate level, the proportion of children not exposed to the risks of stunting or extreme poverty was estimated to be around 63%; 69% of children received home simulation defined as adequate, whereas fewer than two-fifths of 3–4-year-old children participated in ECE programmes in the 65 LMICs with data. Finally, about three-quarters of children were considered to be developmentally on track according to the ECDI.

Children in sub-Saharan Africa countries were most disadvantaged on all four indicators. Children in higher income countries did better on all indicators compared with peers in lower income countries. In terms of inequalities, on average, substantial residence and household wealth disparities were observed in almost all regions and country income groups. In most countries, children living in urban areas or in the wealthiest households fared markedly better on the four indicators compared with their peers in rural areas or in the poorest households. For most countries in which a trend could be estimated, inequalities by gender, area and wealth did not reduce over time.

We found small gender differences in our indicators, mostly favouring girls. The data suggest boys and girls have, on average, relatively similar exposure to ECE and home stimulation. Notwithstanding, compared with boys, girls were, on average, both more likely to have higher rate of the ECDI and less likely to be stunted. Although it is possible that girls’ advantage on the ECDI could be attributed to biased reporting by parents, prior evidence suggests that it is also plausible that girls on average attain the relatively basic social or self-control related skills measured by the ECDI earlier than boys.^29^

The estimates produced in this study differed from those reported in prior work. In terms of risk exposure, the proportion of children not experiencing poverty or stunting increased from the 57% reported in the Lancet Series^11^ to 63% in this study. The Lancet global estimate was based on 2010 data from 141 countries. This study limited to 85 LMICs that have nationally representative population data in order to measure within-country inequalities. The surveys analysed in this paper were mostly (90%) conducted since 2013, and the estimates can thus be viewed as an update of the previous ones in a subsample of countries. As for home stimulation and ECE, a prior study^12^ found that 72% of preschool-aged children in LMICs received adequate levels of stimulation at home and 34% attended ECE, compared with 69% and 39%, respectively, in this study. The primary reason for these differences is that a larger number of surveys were available for this analysis.

The largest differences relative to the previous literature were found for the ECDI. The numbers presented in this paper suggest that 75% of 3–4-year olds were developmentally on track according to the ECDI, which is high compared with the estimates of risk exposure discussed previously. As previous work has shown,^13^ not all items on the ECDI are age-appropriate, and therefore may overestimate children’s developmental competence. Indeed, prior analyses limited to a subset of age-appropriate cognitive and socio-emotional items from the ECDI found that 63% of children were on track,^13^ identical to the updated estimate of the percentage of children without major risk exposure from this analysis.

Given the differences in empirical approaches and data sets used, it is difficult to determine whether the updated numbers produced in this paper represent actual changes—in most cases, improvements—in children’s developmental well-being relative to earlier reports, or versus whether they may be artefacts of different surveys or analytic choices selected across the studies. That said, some country-level trends within this study suggest positive changes: 30 countries experienced statistically significant increases in the proportion of children not exposed to stunting or extreme poverty, and 8 out of 16 countries also experienced increases in the proportion of children participating ECE; in some cases, such as Lao PDR and Iraq, these increases were substantial.

The study has the following limitations. First, although this work is as yet the most comprehensive analysis of available global ECD-related data, it only covers a limited number of LMICs. Therefore the aggregate-level estimates should be interpreted with caution, as they do not necessarily represent global/regional/income-level estimates. In addition, analysis of time trends for the four indicators was possible only in a small number of countries; therefore, we shall not generalise the global or regional trends from the findings in those countries. Further efforts are needed to increase the coverage and frequency of national survey programmes with ECD components in more LMICs. Second, variables on ECE, ECDI and home stimulation are reported by caregivers, which could result in recall bias and misreporting. Third, high-quality, comparable data on the developmental status of children remain limited. The ECDI does not fully capture the developmental domains of children in this age range,^13^ and may thus underestimate the true burden of developmental challenges in LMIC settings. A similar concern applies to the home stimulation measure used in MICS surveys, which cover only the presence or absence of six very basic caregiver–child interactions over a very short period of time. Our use of commonly applied though relatively arbitrary cut-offs for adequate stimulation and on track development may mask important heterogeneity. Future work using the forthcoming updated version of the ECDI—which aims to address limitations in conceptual scope, age range and appropriateness, and psychometric properties—is critical for establishing more accurate estimates of developmental well-being. Fourth, to be consistent with previous studies on the risk of poor childhood development, our study included only two risk factors (stunting and extreme poverty). In future, we aim to include additional risk factors that have been shown to undermine healthy childhood development such as maltreatment during early childhood and low maternal schooling.^3–5^ Fifth, for measures such as ECE and stimulation, our study only focused on access, not frequency, duration nor quality. Further research is needed to quantify these features, and to measure access to quality services and home opportunities for learning across countries and time.

The SDGs have placed between/within-country inequalities at central stage. As such, identifying young children who are at the highest risk of being left behind in their early development is essential for policy making, targeted ameliorative action and evaluation. Our study clearly shows that children in the poorest quintile or in rural areas are being left behind and consistently scoring worse on all four indicators of ECD than peers in the richest quintile or urban areas. In many sub-Saharan countries, more than half of young children were exposed to stunting or extreme poverty, indicators that have robust associations with poorer status on subsequent school learning, labour market productivity and health.^3–5 11 22^ These findings can be used to target efforts and resources to meet SDG 4.2. Although improvements are necessary globally, focusing attention on the poorest countries in sub-Saharan Africa and South Asia, and within those countries, on the poorest families and those living rurally, will help to mitigate the largest inequalities. To advance these efforts, we must also invest more in developing, improving and applying measures relevant to ECD, consistent with the SDGs, in all countries. In addition, more research is needed on socioeconomic barriers and facilitators at the individual/household/societal-levels for reducing inequalities in ECD and ensuring that high-quality, affordable care and education is available to all children.
