# Supplementary material for: Inequalities in early childhood care and development in low/middle-income countries: 2010–2018
Source: BMJ Glob Health. 2020 Feb 4;5(2):e002314. doi: 10.1136/bmjgh-2020-002314 (PMC7042597; doi:10.1136/bmjgh-2020-002314)
Supplement: Supplementary data [file bmjgh-2020-002314supp001.pdf]

Appendix

Chapter 1 Assessment framework and data used in estimation

Table 1 Analytical framework

	Risk exposure	ECE[a]	Home learning	ECDI[b]
**(1) Level of analysis**				
Cross-country	X	X	X	X
Within-country	X	X	X	X
**(2) Inequality analysis**				
Cross-country				
Region	X	X	X	X
Country income group	X	X	X	X
Within-country				
Gender	X	X	X	X
Residential area	X	X	X	X
Household wealth quintiles		X	X	X
**(3) Time frame**				
Most recent years	X	X	X	X
Trends between two rounds	X	X	X	X

a: Early care and education programs
b: Early childhood development index

Table 2 DHS and MICS used for estimating the level four indicators and inequalities in most recent years
(a) % of young children exposed to stunting or extreme poverty

Country	Year	Survey	Country income group	Region
Albania	2017	DHS	Upper-middle	Europe and Central Asia
Algeria	2012	MICS	Upper-middle	Middle East and North Africa
Angola	2015	DHS	Upper-middle	Sub-Saharan Africa
Armenia	2010	DHS	Lower-middle	Europe and Central Asia
Armenia	2016	DHS	Lower-middle	Europe and Central Asia
Bangladesh	2011	DHS	Low-income	South Asia
Bangladesh	2014	DHS	Low-income	South Asia
Belize	2011	MICS	Upper-middle	Latin America and the Caribbean
Belize	2015	MICS	Upper-middle	Latin America and the Caribbean
Benin	2012	DHS	Low-income	Sub-Saharan Africa
Benin	2014	MICS	Low-income	Sub-Saharan Africa
Bhutan	2010	MICS	Lower-middle	South Asia
Bosnia and Herzegovina	2011	MICS	Upper-middle	Europe and Central Asia
Burkina Faso	2010	DHS	Low-income	Sub-Saharan Africa
Burundi	2010	DHS	Low-income	Sub-Saharan Africa
Burundi	2017	DHS	Low-income	Sub-Saharan Africa
Cambodia	2010	DHS	Low-income	East Asia and Pacific
Cambodia	2014	DHS	Low-income	East Asia and Pacific
Cameroon	2011	DHS	Lower-middle	Sub-Saharan Africa
Cameroon	2014	MICS	Lower-middle	Sub-Saharan Africa
Central African Republic	2010	MICS	Low-income	Sub-Saharan Africa
Chad	2010	DHS	Low-income	Sub-Saharan Africa
Chad	2015	DHS	Low-income	Sub-Saharan Africa
Colombia	2010	DHS	Upper-middle	Latin America and the Caribbean
Comoros	2012	DHS	Low-income	Sub-Saharan Africa
Congo	2011	DHS	Lower-middle	Sub-Saharan Africa
Congo	2015	MICS	Lower-middle	Sub-Saharan Africa
Congo DR	2010	MICS	Low-income	Sub-Saharan Africa
Congo DR	2013	DHS	Low-income	Sub-Saharan Africa
Cote d'Ivoire	2012	DHS	Lower-middle	Sub-Saharan Africa
Cote d'Ivoire	2016	MICS	Lower-middle	Sub-Saharan Africa
Dominican Republic	2013	DHS	Upper-middle	Latin America and the Caribbean
Egypt	2014	DHS	Lower-middle	Middle East and North Africa

El Salvador	2014	MICS	Lower-middle	Latin America and the Caribbean
Ethiopia	2011	DHS	Low-income	Sub-Saharan Africa
Ethiopia	2016	DHS	Low-income	Sub-Saharan Africa
Gabon	2012	DHS	Upper-middle	Sub-Saharan Africa
Gambia	2010	MICS	Low-income	Sub-Saharan Africa
Gambia	2013	DHS	Low-income	Sub-Saharan Africa
Ghana	2011	MICS	Lower-middle	Sub-Saharan Africa
Ghana	2014	DHS	Lower-middle	Sub-Saharan Africa
Guatemala	2014	DHS	Lower-middle	Latin America and the Caribbean
Guinea	2012	DHS	Low-income	Sub-Saharan Africa
Guinea	2016	MICS	Low-income	Sub-Saharan Africa
Guinea Bissau	2014	MICS	Low-income	Sub-Saharan Africa
Guyana	2014	MICS	Lower-middle	Latin America and the Caribbean
Haiti	2012	DHS	Low-income	Latin America and the Caribbean
Haiti	2017	DHS	Low-income	Latin America and the Caribbean
Honduras	2012	DHS	Lower-middle	Latin America and the Caribbean
India	2015	DHS	Lower-middle	South Asia
Iraq	2011	MICS	Upper-middle	Middle East and North Africa
Iraq	2018	MICS	Upper-middle	Middle East and North Africa
Jordan	2012	DHS	Upper-middle	Middle East and North Africa
Kazakhstan	2010	MICS	Upper-middle	Europe and Central Asia
Kazakhstan	2015	MICS	Upper-middle	Europe and Central Asia
Kenya	2014	DHS	Low-income	Sub-Saharan Africa
Kosovo	2014	MICS	Lower-middle	Europe and Central Asia
Kyrgyzstan	2012	DHS	Lower-middle	Europe and Central Asia
Kyrgyzstan	2014	MICS	Lower-middle	Europe and Central Asia
Lao	2011	MICS	Lower-middle	East Asia and Pacific
Lao	2017	MICS	Lower-middle	East Asia and Pacific
Lebanon	2011	MICS	Upper-middle	Middle East and North Africa
Lesotho	2014	DHS	Lower-middle	Sub-Saharan Africa
Liberia	2013	DHS	Low-income	Sub-Saharan Africa
Macedonia	2011	MICS	Upper-middle	Europe and Central Asia
Malawi	2010	DHS	Low-income	Sub-Saharan Africa
Malawi	2015	DHS	Low-income	Sub-Saharan Africa
Maldives	2017	DHS	Upper-middle	South Asia
Mali	2010	MICS	Low-income	Sub-Saharan Africa
Mali	2015	MICS	Low-income	Sub-Saharan Africa
Mauritania	2011	MICS	Lower-middle	Sub-Saharan Africa
Mauritania	2015	MICS	Lower-middle	Sub-Saharan Africa

Mexico	2015	MICS	Upper-middle	Latin America and the Caribbean
Moldova	2012	MICS	Lower-middle	Europe and Central Asia
Mongolia	2010	MICS	Lower-middle	East Asia and Pacific
Mongolia	2013	MICS	Lower-middle	East Asia and Pacific
Montenegro	2013	MICS	Upper-middle	Europe and Central Asia
Mozambique	2011	DHS	Low-income	Sub-Saharan Africa
Myanmar	2016	DHS	Low-income	East Asia and Pacific
Namibia	2013	DHS	Upper-middle	Sub-Saharan Africa
Nepal	2011	DHS	Low-income	South Asia
Nepal	2016	DHS	Low-income	South Asia
Niger	2012	DHS	Low-income	Sub-Saharan Africa
Nigeria	2011	MICS	Lower-middle	Sub-Saharan Africa
Nigeria	2016	MICS	Lower-middle	Sub-Saharan Africa
Pakistan	2013	DHS	Lower-middle	South Asia
Pakistan	2018	DHS	Lower-middle	South Asia
Palestine	2010	MICS	Low-income	Middle East and North Africa
Palestine	2014	MICS	Low-income	Middle East and North Africa
Paraguay	2016	MICS	Lower-middle	Latin America and the Caribbean
Peru	2010	DHS	Upper-middle	Latin America and the Caribbean
Peru	2012	DHS	Upper-middle	Latin America and the Caribbean
Rwanda	2010	DHS	Low-income	Sub-Saharan Africa
Rwanda	2015	DHS	Low-income	Sub-Saharan Africa
STP	2014	MICS	Lower-middle	Sub-Saharan Africa
Senegal	2011	DHS	Lower-middle	Sub-Saharan Africa
Senegal	2017	DHS	Lower-middle	Sub-Saharan Africa
Serbia	2010	MICS	Upper-middle	Europe and Central Asia
Serbia	2014	MICS	Upper-middle	Europe and Central Asia
Sierra Leone	2010	MICS	Low-income	Sub-Saharan Africa
Sierra Leone	2017	MICS	Low-income	Sub-Saharan Africa
South Africa	2016	DHS	Upper-middle	Sub-Saharan Africa
St. Lucia	2012	MICS	Upper-middle	Latin America and the Caribbean
Sudan	2014	MICS	Lower-middle	Sub-Saharan Africa
Suriname	2010	MICS	Upper-middle	Latin America and the Caribbean
Swaziland	2010	MICS	Lower-middle	Sub-Saharan Africa
Swaziland	2014	MICS	Lower-middle	Sub-Saharan Africa
Tajikistan	2012	DHS	Low-income	Europe and Central Asia
Tajikistan	2017	DHS	Low-income	Europe and Central Asia
Tanzania	2010	DHS	Low-income	Sub-Saharan Africa
Tanzania	2015	DHS	Low-income	Sub-Saharan Africa

Thailand	2012	MICS	Upper-middle	East Asia and Pacific
Thailand	2016	MICS	Upper-middle	East Asia and Pacific
Timor-Leste	2016	DHS	Lower-middle	East Asia and Pacific
Togo	2010	MICS	Low-income	Sub-Saharan Africa
Togo	2014	DHS	Low-income	Sub-Saharan Africa
Tunisia	2012	MICS	Upper-middle	Middle East and North Africa
Turkmenistan	2015	MICS	Upper-middle	Europe and Central Asia
Uganda	2011	DHS	Low-income	Sub-Saharan Africa
Uganda	2016	DHS	Low-income	Sub-Saharan Africa
Vietnam	2011	MICS	Lower-middle	East Asia and Pacific
Yemen	2013	DHS	Lower-middle	Middle East and North Africa
Zambia	2013	DHS	Lower-middle	Sub-Saharan Africa
Zimbabwe	2010	DHS	Low-income	Sub-Saharan Africa
Zimbabwe	2015	DHS	Low-income	Sub-Saharan Africa

(b) ECE

Country	Year	Survey	Country income group	Region
Afghanistan	2010	MICS	Low-income	South Asia
Algeria	2012	MICS	Upper-middle	Middle East and North Africa
Argentina	2011	MICS	Upper-middle	Latin America and the Caribbean
Bangladesh	2012	MICS	Low-income	South Asia
Belarus	2012	MICS	Upper-middle	Europe and Central Asia
Belize	2011	MICS	Upper-middle	Latin America and the Caribbean
Belize	2015	MICS	Upper-middle	Latin America and the Caribbean
Benin	2014	MICS	Low-income	Sub-Saharan Africa
Bhutan	2010	MICS	Lower-middle	South Asia
Bosnia and Herzegovina	2011	MICS	Upper-middle	Europe and Central Asia
Burundi	2016	DHS	Low-income	Sub-Saharan Africa
Cameroon	2011	DHS	Lower-middle	Sub-Saharan Africa
Cameroon	2014	MICS	Lower-middle	Sub-Saharan Africa
Central African Republic	2010	MICS	Low-income	Sub-Saharan Africa
Chad	2010	DHS	Low-income	Sub-Saharan Africa
Chad	2014	MICS	Low-income	Sub-Saharan Africa
Congo, Dem. Rep.	2010	MICS	Low-income	Sub-Saharan Africa
Congo, Dem. Rep.	2013	DHS	Low-income	Sub-Saharan Africa
Congo, Rep.	2014	MICS	Lower-middle	Sub-Saharan Africa
Costa Rica	2011	MICS	Upper-middle	Latin America and the Caribbean
Cote d'Ivoire	2016	MICS	Lower-middle	Sub-Saharan Africa
Dominican Republic	2014	MICS	Upper-middle	Latin America and the Caribbean

El Salvador	2014	MICS	Lower-middle	Latin America and the Caribbean
Gambia	2010	MICS	Low-income	Sub-Saharan Africa
Ghana	2011	MICS	Lower-middle	Sub-Saharan Africa
Guinea	2016	MICS	Low-income	Sub-Saharan Africa
Guinea-Bissau	2014	MICS	Low-income	Sub-Saharan Africa
Guyana	2014	MICS	Lower-middle	Latin America and the Caribbean
Iraq	2011	MICS	Upper-middle	Middle East and North Africa
Iraq	2018	MICS	Upper-middle	Middle East and North Africa
Jamaica	2011	MICS	Upper-middle	Latin America and the Caribbean
Jordan	2012	DHS	Upper-middle	Middle East and North Africa
Kazakhstan	2010	MICS	Upper-middle	Europe and Central Asia
Kazakhstan	2015	MICS	Upper-middle	Europe and Central Asia
Kosovo	2013	MICS	Lower-middle	Europe and Central Asia
Kyrgyzstan	2014	MICS	Lower-middle	Europe and Central Asia
Lao PDR	2011	MICS	Lower-middle	East Asia and Pacific
Lao PDR	2017	MICS	Lower-middle	East Asia and Pacific
Lebanon	2011	MICS	Upper-middle	Middle East and North Africa
Macedonia	2011	MICS	Upper-middle	Europe and Central Asia
Malawi	2013	MICS	Low-income	Sub-Saharan Africa
Maldives	2016	DHS	Upper-middle	South Asia
Mali	2015	MICS	Low-income	Sub-Saharan Africa
Mauritania	2015	MICS	Lower-middle	Sub-Saharan Africa
Mexico	2015	MICS	Upper-middle	Latin America and the Caribbean
Moldova	2012	MICS	Lower-middle	Europe and Central Asia
Mongolia	2010	MICS	Lower-middle	East Asia and Pacific
Mongolia	2013	MICS	Lower-middle	East Asia and Pacific
Montenegro	2013	MICS	Upper-middle	Europe and Central Asia
Nepal	2014	MICS	Low-income	South Asia
Nigeria	2011	MICS	Lower-middle	Sub-Saharan Africa
Nigeria	2016	MICS	Lower-middle	Sub-Saharan Africa
Palestine	2010	MICS	Low-income	Middle East and North Africa
Palestine	2014	MICS	Low-income	Middle East and North Africa
Panama	2013	MICS	Upper-middle	Latin America and the Caribbean
Paraguay	2016	MICS	Lower-middle	Latin America and the Caribbean
Rwanda	2014	DHS	Low-income	Sub-Saharan Africa
Senegal	2017	DHS	Lower-middle	Sub-Saharan Africa
Serbia	2010	MICS	Upper-middle	Europe and Central Asia
Serbia	2014	MICS	Upper-middle	Europe and Central Asia
Sierra Leone	2010	MICS	Low-income	Sub-Saharan Africa
Sierra Leone	2017	MICS	Low-income	Sub-Saharan Africa
South Sudan	2010	MICS	Lower-middle	Sub-Saharan Africa
Suriname	2010	MICS	Upper-middle	Latin America and the Caribbean
Swaziland	2010	MICS	Lower-middle	Sub-Saharan Africa

Swaziland	2014	MICS	Lower-middle	Sub-Saharan Africa
São Tomé and Principe	2014	MICS	Lower-middle	Sub-Saharan Africa
Thailand	2012	MICS	Upper-middle	East Asia and Pacific
Thailand	2015	MICS	Upper-middle	East Asia and Pacific
Timor-Leste	2016	DHS	Lower-middle	East Asia and Pacific
Togo	2010	MICS	Low-income	Sub-Saharan Africa
Trinidad and Tobago	2011	MICS	Upper-middle	Latin America and the Caribbean
Tunisia	2011	MICS	Upper-middle	Middle East and North Africa
Turkmenistan	2015	MICS	Upper-middle	Europe and Central Asia
Uganda	2016	DHS	Low-income	Sub-Saharan Africa
Ukraine	2012	MICS	Lower-middle	Europe and Central Asia
Uruguay	2012	MICS	Upper-middle	Latin America and the Caribbean
Vietnam	2011	MICS	Lower-middle	East Asia and Pacific
Vietnam	2013	MICS	Lower-middle	East Asia and Pacific
Zimbabwe	2014	MICS	Low-income	Sub-Saharan Africa

(c) Home stimulation

Country	Year	Survey	Country income group	Region
Afghanistan	2010	MICS	Low-income	South Asia
Algeria	2012	MICS	Upper-middle	Middle East and North Africa
Argentina	2011	MICS	Upper-middle	Latin America and the Caribbean
Bangladesh	2012	MICS	Low-income	South Asia
Belarus	2012	MICS	Upper-middle	Europe and Central Asia
Belize	2011	MICS	Upper-middle	Latin America and the Caribbean
Belize	2015	MICS	Upper-middle	Latin America and the Caribbean
Benin	2014	MICS	Low-income	Sub-Saharan Africa
Bhutan	2010	MICS	Lower-middle	South Asia
Bosnia and Herzegovina	2011	MICS	Upper-middle	Europe and Central Asia
Burundi	2016	DHS	Low-income	Sub-Saharan Africa
Cameroon	2014	MICS	Lower-middle	Sub-Saharan Africa
Central African Republic	2010	MICS	Low-income	Sub-Saharan Africa
Congo, Rep.	2014	MICS	Lower-middle	Sub-Saharan Africa
Costa Rica	2011	MICS	Upper-middle	Latin America and the Caribbean
Cote d'Ivoire	2016	MICS	Lower-middle	Sub-Saharan Africa
Dominican Republic	2014	MICS	Upper-middle	Latin America and the Caribbean
El Salvador	2014	MICS	Lower-middle	Latin America and the Caribbean
Gambia	2010	MICS	Low-income	Sub-Saharan Africa
Ghana	2011	MICS	Lower-middle	Sub-Saharan Africa
Guinea	2016	MICS	Low-income	Sub-Saharan Africa
Guinea-Bissau	2014	MICS	Low-income	Sub-Saharan Africa
Guyana	2014	MICS	Lower-middle	Latin America and the Caribbean
Iraq	2011	MICS	Upper-middle	Middle East and North Africa

Iraq	2018	MICS	Upper-middle	Middle East and North Africa
Jamaica	2011	MICS	Upper-middle	Latin America and the Caribbean
Jordan	2012	DHS	Upper-middle	Middle East and North Africa
Kazakhstan	2010	MICS	Upper-middle	Europe and Central Asia
Kazakhstan	2015	MICS	Upper-middle	Europe and Central Asia
Kosovo	2013	MICS	Lower-middle	Europe and Central Asia
Kyrgyzstan	2014	MICS	Lower-middle	Europe and Central Asia
Lao PDR	2011	MICS	Lower-middle	East Asia and Pacific
Lao PDR	2017	MICS	Lower-middle	East Asia and Pacific
Lebanon	2011	MICS	Upper-middle	Middle East and North Africa
Macedonia	2011	MICS	Upper-middle	Europe and Central Asia
Malawi	2013	MICS	Low-income	Sub-Saharan Africa
Maldives	2016	DHS	Upper-middle	South Asia
Mali	2015	MICS	Low-income	Sub-Saharan Africa
Mauritania	2011	MICS	Lower-middle	Sub-Saharan Africa
Mauritania	2015	MICS	Lower-middle	Sub-Saharan Africa
Mexico	2015	MICS	Upper-middle	Latin America and the Caribbean
Moldova	2012	MICS	Lower-middle	Europe and Central Asia
Mongolia	2010	MICS	Lower-middle	East Asia and Pacific
Mongolia	2013	MICS	Lower-middle	East Asia and Pacific
Montenegro	2013	MICS	Upper-middle	Europe and Central Asia
Nepal	2014	MICS	Low-income	South Asia
Nigeria	2011	MICS	Lower-middle	Sub-Saharan Africa
Nigeria	2016	MICS	Lower-middle	Sub-Saharan Africa
Palestine	2010	MICS	Low-income	Middle East and North Africa
Palestine	2014	MICS	Low-income	Middle East and North Africa
Panama	2013	MICS	Upper-middle	Latin America and the Caribbean
Paraguay	2016	MICS	Lower-middle	Latin America and the Caribbean
Rwanda	2014	DHS	Low-income	Sub-Saharan Africa
Senegal	2017	DHS	Lower-middle	Sub-Saharan Africa
Serbia	2010	MICS	Upper-middle	Europe and Central Asia
Serbia	2014	MICS	Upper-middle	Europe and Central Asia
Sierra Leone	2010	MICS	Low-income	Sub-Saharan Africa
Sierra Leone	2017	MICS	Low-income	Sub-Saharan Africa
Suriname	2010	MICS	Upper-middle	Latin America and the Caribbean
Swaziland	2010	MICS	Lower-middle	Sub-Saharan Africa
Swaziland	2014	MICS	Lower-middle	Sub-Saharan Africa
São Tomé and Principe	2014	MICS	Lower-middle	Sub-Saharan Africa
Thailand	2012	MICS	Upper-middle	East Asia and Pacific
Thailand	2015	MICS	Upper-middle	East Asia and Pacific
Timor-Leste	2016	DHS	Lower-middle	East Asia and Pacific
Togo	2010	MICS	Low-income	Sub-Saharan Africa
Trinidad and Tobago	2011	MICS	Upper-middle	Latin America and the Caribbean

Tunisia	2011	MICS	Upper-middle	Middle East and North Africa
Turkmenistan	2015	MICS	Upper-middle	Europe and Central Asia
Uganda	2016	DHS	Low-income	Sub-Saharan Africa
Ukraine	2012	MICS	Lower-middle	Europe and Central Asia
Uruguay	2012	MICS	Upper-middle	Latin America and the Caribbean
Vietnam	2010	MICS	Lower-middle	East Asia and Pacific
Vietnam	2013	MICS	Lower-middle	East Asia and Pacific
Zimbabwe	2014	MICS	Low-income	Sub-Saharan Africa

(d) ECDI

Country	Year	Survey	Country income group	Region
Algeria	2012	MICS	Upper-middle	Middle East and North Africa
Argentina	2011	MICS	Upper-middle	Latin America and the Caribbean
Bangladesh	2012	MICS	Low-income	South Asia
Belarus	2012	MICS	Upper-middle	Europe and Central Asia
Belize	2011	MICS	Upper-middle	Europe and Central Asia
Belize	2015	MICS	Upper-middle	Latin America and the Caribbean
Benin	2014	MICS	Low-income	Sub-Saharan Africa
Bhutan	2010	MICS	Lower-middle	South Asia
Bosnia and Herzegovina	2011	MICS	Upper-middle	Europe and Central Asia
Burundi	2016	DHS	Low-income	Sub-Saharan Africa
Cameroon	2011	DHS	Lower-middle	Sub-Saharan Africa
Cameroon	2014	MICS	Lower-middle	Sub-Saharan Africa
Central African Republic	2010	MICS	Low-income	Sub-Saharan Africa
Chad	2010	DHS	Low-income	Sub-Saharan Africa
Chad	2014	MICS	Low-income	Sub-Saharan Africa
Congo, Dem. Rep.	2010	MICS	Low-income	Sub-Saharan Africa
Congo, Dem. Rep.	2013	DHS	Low-income	Sub-Saharan Africa
Congo, Rep.	2014	MICS	Lower-middle	Sub-Saharan Africa
Costa Rica	2011	MICS	Upper-middle	Latin America and the Caribbean
Cote d'Ivoire	2016	MICS	Lower-middle	Sub-Saharan Africa
Dominican Republic	2014	MICS	Upper-middle	Latin America and the Caribbean
El Salvador	2014	MICS	Lower-middle	Latin America and the Caribbean
Gambia	2010	MICS	Low-income	Sub-Saharan Africa
Ghana	2011	MICS	Lower-middle	Sub-Saharan Africa
Guinea	2016	MICS	Low-income	Sub-Saharan Africa
Guinea-Bissau	2014	MICS	Low-income	Sub-Saharan Africa
Guyana	2014	MICS	Lower-middle	Latin America and the Caribbean
Jamaica	2011	MICS	Upper-middle	Latin America and the Caribbean
Jordan	2012	DHS	Upper-middle	Middle East and North Africa
Kazakhstan	2010	MICS	Upper-middle	Sub-Saharan Africa
Kazakhstan	2015	MICS	Upper-middle	Europe and Central Asia

Kosovo	2013	MICS	Lower-middle	Europe and Central Asia
Kyrgyzstan	2014	MICS	Lower-middle	Europe and Central Asia
Lebanon	2011	MICS	Upper-middle	Middle East and North Africa
Macedonia	2011	MICS	Upper-middle	Europe and Central Asia
Malawi	2013	MICS	Low-income	Sub-Saharan Africa
Maldives	2016	DHS	Upper-middle	South Asia
Mali	2015	MICS	Low-income	Sub-Saharan Africa
Mauritania	2011	MICS	Lower-middle	Sub-Saharan Africa
Mauritania	2015	MICS	Lower-middle	Sub-Saharan Africa
Mexico	2015	MICS	Upper-middle	Latin America and the Caribbean
Moldova	2012	MICS	Lower-middle	Europe and Central Asia
Mongolia	2010	MICS	Lower-middle	East Asia and Pacific
Mongolia	2013	MICS	Lower-middle	East Asia and Pacific
Montenegro	2013	MICS	Upper-middle	Europe and Central Asia
Nepal	2014	MICS	Low-income	South Asia
Nigeria	2011	MICS	Lower-middle	Sub-Saharan Africa
Nigeria	2016	MICS	Lower-middle	Sub-Saharan Africa
Palestine	2010	MICS	Low-income	Middle East and North Africa
Palestine	2014	MICS	Low-income	Middle East and North Africa
Panama	2013	MICS	Upper-middle	Latin America and the Caribbean
Paraguay	2016	MICS	Lower-middle	Latin America and the Caribbean
Rwanda	2014	DHS	Low-income	Sub-Saharan Africa
Senegal	2017	DHS	Lower-middle	Sub-Saharan Africa
Serbia	2010	MICS	Upper-middle	Europe and Central Asia
Serbia	2014	MICS	Upper-middle	Europe and Central Asia
Suriname	2010	MICS	Upper-middle	Latin America and the Caribbean
Swaziland	2010	MICS	Lower-middle	Sub-Saharan Africa
Swaziland	2014	MICS	Lower-middle	Sub-Saharan Africa
São Tomé and Principe	2014	MICS	Lower-middle	Sub-Saharan Africa
Thailand	2012	MICS	Upper-middle	East Asia and Pacific
Thailand	2015	MICS	Upper-middle	East Asia and Pacific
Timor-Leste	2016	DHS	Lower-middle	East Asia and Pacific
Togo	2010	MICS	Low-income	Sub-Saharan Africa
Trinidad and Tobago	2011	MICS	Upper-middle	Latin America and the Caribbean
Tunisia	2011	MICS	Upper-middle	Middle East and North Africa
Turkmenistan	2015	MICS	Upper-middle	Europe and Central Asia
Uganda	2016	DHS	Low-income	Sub-Saharan Africa
Ukraine	2012	MICS	Lower-middle	Europe and Central Asia
Uruguay	2012	MICS	Upper-middle	Latin America and the Caribbean
Vietnam	2010	MICS	Lower-middle	East Asia and Pacific
Vietnam	2013	MICS	Lower-middle	East Asia and Pacific
Zimbabwe	2014	MICS	Low-income	Sub-Saharan Africa

Chapter 2 Constructing micro-level variable on extreme poverty status in DHS and MICS

We followed the method used in a previous study on children exposed to stunting or extreme poverty.[1] All the Demographic & Health Surveys[2] and Multiple Indicator Cluster Surveys[3] data included variables indicating a household wealth index scores. We estimated the % of under-five children living in extreme poverty ($1.9 per day) with the following steps.

Firstly, we obtained a country's poverty rate at $1.9 per day (2011 PPP) from the World Bank.[4]There were 121 countries with estimates available in some years between 2000 and 2018. Among the 85 countries under this study, the World Bank has poverty estimates available for 34 countries.

Secondly, we followed previous studies[1] and imputed poverty rates for countries and years without available data using the following methods: average poverty rates by income group, regression with generalized linear model (GLM) for prediction, linear extrapolation, or replacing the missing with the value in the closest year. We compared these methods in terms of their root-mean-square errors (RMSE) using existing estimates between 2000 and 2018. The RMSE measures the difference between predicted values and the observed values and is calculated as the square root of the mean of the squares of the deviations between predicted values and observed values. The smaller the RMSE is, the closer is the imputed value to the observed value, and the more accurate of the imputed value is.[5] To calculate the RMSE, we first randomly selected 20% of the existing poverty estimates in 121 countries between 2000 and 2018 and assigned them with missing values. We then predicted the values for those 20% using the 80% of existing estimates and the four imputation methods. *With the average income method*, we obtained the average poverty ratio of the 80% data by income group and then assigned them to the 20% data according to a country's corresponding income group. *With the closest-year method*, we assigned the estimate in the year that was closest to the year with missing data. *With the GLM method*, we used a generalized linear model with a logit link and binomial distribution. This model is a commonly used with dependent variable as proportion data (values between zero and one).[6] Following previous studies,[1] we generated predicted values for the 20% missing values by regressing poverty estimates on GDP per capita. *With the linear extrapolation or interpolation method*, we first checked the assumption of approximately linear trends of poverty estimates by graphing the trends of poverty ratios for countries with 3 data points or more between 2000 and 2018. We found that most of the countries in most years followed approximately linear trends (**Figure 1**). This method has been used to impute missing values for poverty ratios in previous studies[7,8] and the limitation of this assumption is that we had to assume no change in distribution of poverty.

We obtained the RMSE for each method as shown in **Table 1**. Among the four methods, linear interpolation/extrapolation has the smallest RMSE, followed by the closest-year, GLM regression, and average income. We calculated the correlation between actual values and the predicted values derived from the four methods: the correlation between the actual values and the predicted values from the linear extrapolation is the highest (0.98), followed by predicted values derived from the closest-year method (0.97), average income (0.64), and GLM regression (0.64).

Based on these findings, we decided that (1) for countries with two or more data points, we replaced the missing values with the predicted values derived from the linear extrapolation method. For those years with predicted values equal to or greater than 1 or less than zero, we replaced them with the existing poverty estimates in the closest year; (2) for countries with only one data point between 2000 and 2018, we replaced the missing values with the available poverty estimates; (3) for countries without poverty estimates between 2000 and 2018, we replaced the missing data with the predicted values derived from the GLM.

Thirdly, we derived % of population living in extreme poverty by ranking the wealth index score of the sampled household members with household weights. We applied a country's poverty rate to the ranked wealth scores and located the cutoff point of extreme poverty in the ranked wealth index. For example, according to the World Bank, the 2016 extreme poverty headcount ratio in Uganda is 42%. We used the Uganda DHS 2016 and ranked its household members' wealth index score with household weights. We then located the 42th percentile of the ranked scores as the cutoff point for the extreme poverty. All under-five children with wealth score below the 42th percentile were considered to be living in extreme poverty.

References

1. Lu C, Black MM, Richter LM. Risk of poor development in young children in low-income and middle-income countries: an estimation and analysis at the global, regional, and country level. Lancet Glob Health 2016; **4**:e916–e922.
2. http://www.dhsprogram.com/Data/ (accessed September 6, 2014)
3. United Nations Children's Fund. Multiple indicator cluster surveys. UNICEF 2014
4. https://data.worldbank.org/indicator/SI.POV.DDAY?locations=1W
5. Willmott, Cort; Matsuura, Kenji (2006). "On the use of dimensioned measures of error to evaluate the performance of spatial interpolators". International Journal of Geographic Information Science. **20**: 89–102.
6. Baum CF. Modeling proportions. The Stata Journal (2008), No 2, pp. 299-303.
7. Chen S, Ravallion M. How have the world's poorest fared since the early 1980s? World Bank Research Observer, 01 September 2004, Vol.19(2), pp.141-170.
8. Chen S, Ravallion M. The developing world is poorer than we thought, but no less successful in the fight against poverty. Quarterly Journal Of Economics, 2010 Nov, Vol.125(4), pp.1577-1625.

Table 1 RMSE for predicting extreme poverty estimates derived from four different methods

**Imputation methods**	Root-mean-square-errors
Linear interpolation/extrapolation	0.026
Closest year	0.031
GLM regression	0.105
Average income	0.108

Figure 1 Country-level trends of extreme poverty ratios

Low-income countries

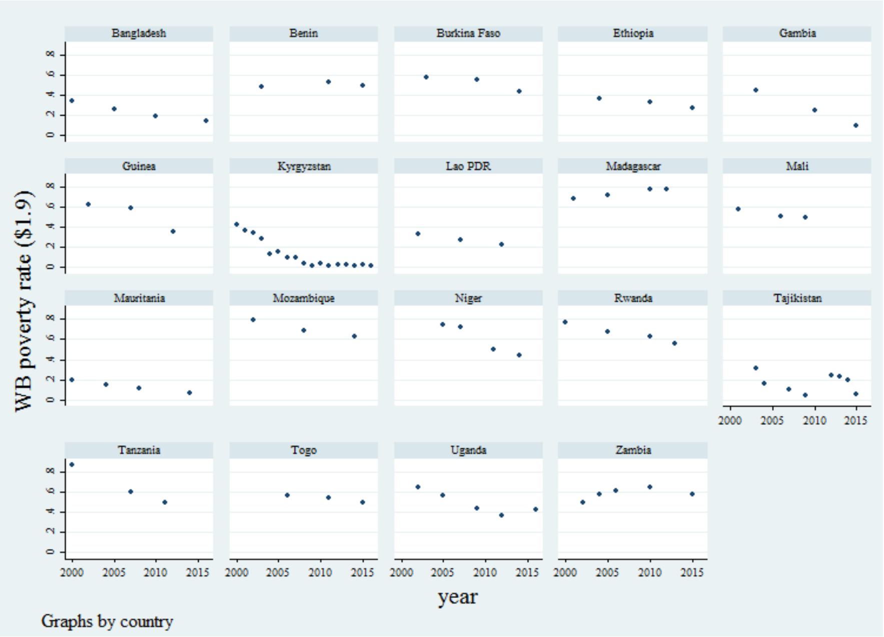

Lower-middle income countries

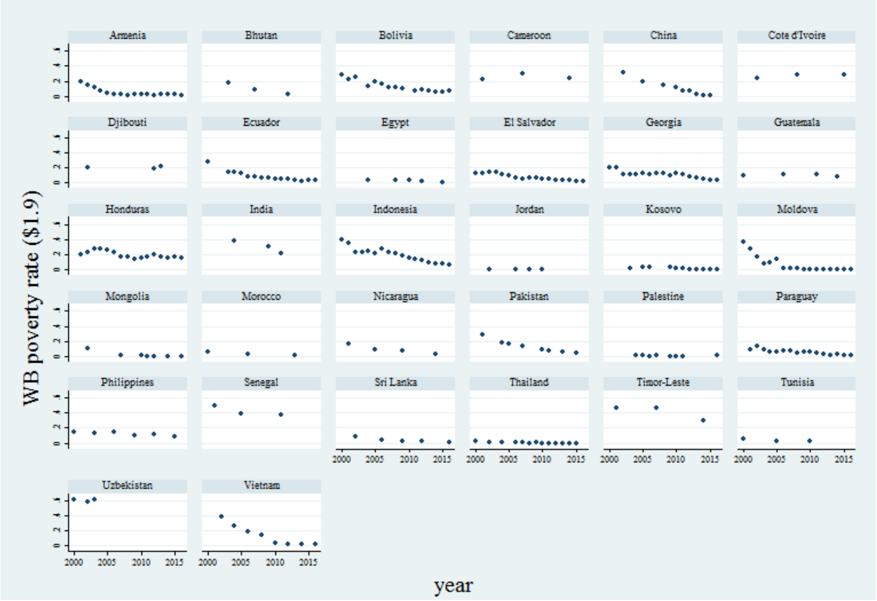

Upper-middle income countries

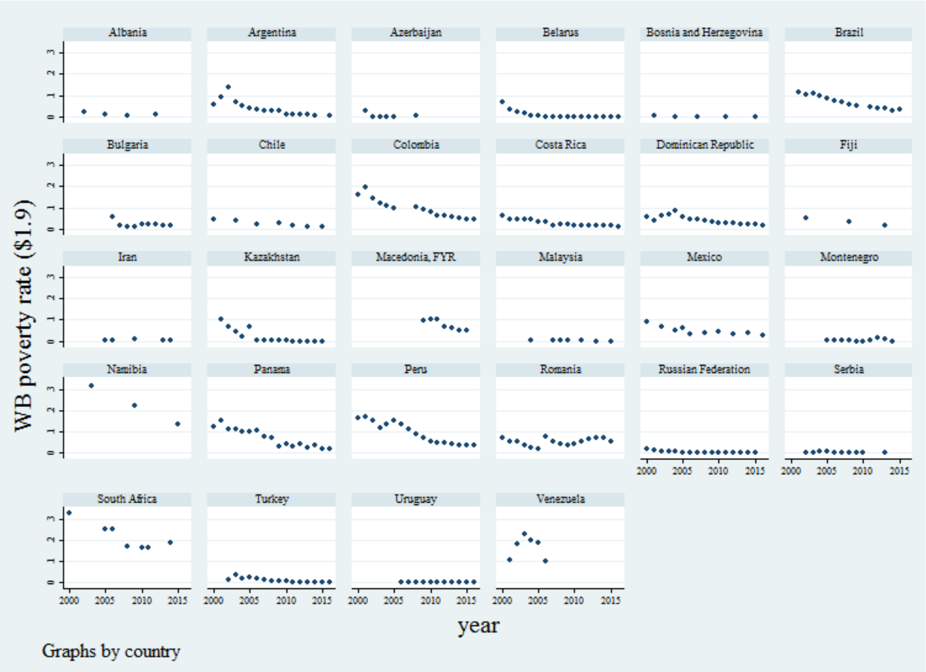
Albania
Argentina
Azerbaijan
Belarus
Bosnia and Herzegovina
Brazil
Bulgaria
Chile
Colombia
Costa Rica
Dominican Republic
Fiji
Iran
Kazakhstan
Macedonia, FYR
Malaysia
Mexico
Montenegro
Namibia
Panama
Peru
Romania
Russian Federation
Serbia
South Africa
Turkey
Uruguay
Venezuela
WB poverty rate ($1.9)
year
Graphs by country

14

Chapter 3 Measuring the level and trends of inequalities in four indicators

(1) Calculating aggregate-level mean values and their 95% confidence intervals (95% CI) of the inequalities in the most recent years since 2010

Following previous studies (1,2), the mean values as well as 95% CI at the aggregate level were computed by averaging country-level estimates. Taking inequality of prevalence of children exposure to the two risk factors by gender in 85 countries as an example. In a first step, we estimated mean difference (absolute inequality) by gender (and their 95% confidence intervals) and adjusted for the sample design, including sample weights, primary sampling units (psu), and strata. In the second step, we obtained aggregate-level inequality by averaging over country-level mean difference across the countries and computed 95% CI of the aggregate-level inequality using

$$\bar{X} \pm Z_{\alpha}\frac{S}{\sqrt{N}} \qquad (1)$$

Where $\bar{X}$ represents the aggregate-level mean inequality, $Z\alpha$ is the z-value (Z=1.96 for 95% CI) and S is the standard deviation of the means of the 85 countries. N is the number of countries (85).

Among the 85 countries, four countries (Uganda 2011, Lao 2017, Palestine 2010, and Sierra Leone 2017) have no information on psu or strata. We were not able to generate national-level estimates adjusted for these variables and therefore used the sample means and 95% CI in these countries by assuming that the sample was randomly drawn from the populations directly.

Reference

1. Barros AJ, Ronsmans C, Axelson H, Loaiza E, Bertoldi AD, França GV, et al. Equity in maternal, newborn, and child health interventions in Countdown to 2015: a retrospective review of survey data from 54 countries. Lancet. 2012;379(9822):1225–33.
2. Li Z, Li M, Subramanian S V, Lu C. Assessing levels and trends of child health inequality in 88 developing countries: from 2000 to 2014. Glob Health Action. 2017;10(1):1408385.

(2) Trend analysis for four indicators at the country level

We conducted trend analysis on inequalities at the country level for countries with data available in the two rounds.

Taking inequality of prevalence of risk exposure by gender as an example. For each country with data in two rounds, we first conducted logit regression (adjusted for sample design) with a dichotomous variable indicating a child's status of being not exposed to stunting and poverty as the outcome variable, and gender, round indicator, and interaction between gender and round indicator as the independent variable. Then we used linear combination to generate the absolute difference of inequalities (and their 95% confidence intervals) by gender between the two rounds.

Chapter 4 Country-level estimates for four indicators in the most recent years since 2010

(1) Not exposed to stunting or extreme poverty

Table 1. Prevalence (%) of children NOT stunted or in extreme poverty by gender with 95% confidence intervals, the most recent years in 85 countries

Country	Year	National	Male	Female	Difference
Albania	2017	86.2(83.8, 88.7)	84.7(81.0, 88.5)	87.7(84.5, 91.0)	-3.0(-8.2, 2.2)
Algeria	2012	88.2(87.3, 89.1)	87.3(86.1, 88.5)	89.1(88.0, 90.2)	-1.8(-3.1, -0.5)
Angola	2015	48.5(46.1, 50.9)	45.5(42.6, 48.4)	51.5(48.6, 54.4)	-6.0(-9.4, -2.6)
Armenia	2016	89.6(87.7, 91.5)	88.3(85.8, 90.7)	91.1(88.6, 93.6)	-2.8(-6.0, 0.4)
Bangladesh	2014	53.8(51.5, 56.2)	53.7(51.0, 56.3)	54.0(51.1, 57.0)	-0.4(-3.2, 2.5)
Belize	2015	82.4(80.1, 84.7)	81.0(78.0, 84.1)	83.9(81.2, 86.6)	-2.9(-6.3, 0.6)
Benin	2014	54.5(54.5, 54.5)	53.0(53.0, 53.0)	55.9(55.9, 55.9)	-2.9(-2.9, -2.9)
Bhutan	2010	63.5(63.5, 63.5)	63.7(63.7, 63.7)	63.4(63.4, 63.4)	0.3(0.3, 0.3)
Bosnia and Herzegovina	2011	91.1(91.1, 91.1)	91.0(91.0, 91.0)	91.1(91.1, 91.1)	-0.1(-0.1, -0.1)
Burkina Faso	2010	29.7(27.8, 31.6)	29.4(27.2, 31.6)	30.0(27.7, 32.3)	-0.6(-3.1, 1.9)
Burundi	2017	14.5(12.8, 16.3)	14.0(12.4, 15.7)	15.1(12.8, 17.3)	-1.1(-2.9, 0.7)
Cambodia	2014	46.1(43.2, 49.1)	46.5(42.8, 50.1)	45.8(42.5, 49.2)	0.6(-3.2, 4.4)
Cameroon	2014	48.1(45.8, 50.4)	47.2(44.5, 50.0)	49.1(46.3, 51.9)	-1.9(-4.9, 1.2)
Central African Republic	2010	23.5(21.6, 25.3)	21.9(19.9, 23.9)	25.0(22.8, 27.1)	-3.1(-4.9, -1.2)
Chad	2015	42.0(39.9, 44.0)	41.9(39.5, 44.3)	42.0(39.6, 44.4)	-0.1(-2.5, 2.3)
Colombia	2010	81.9(80.8, 83.0)	80.8(79.5, 82.2)	83.0(81.6, 84.3)	-2.1(-3.7, -0.6)
Comoros	2012	57.7(54.2, 61.1)	55.2(50.7, 59.8)	60.1(55.7, 64.4)	-4.8(-10.6, 0.9)
Congo	2015	68.3(66.5, 70.2)	67.8(65.6, 70.1)	68.8(66.6, 71.1)	-1.0(-3.6, 1.5)
Congo DR	2013	18.1(15.7, 20.6)	17.9(15.1, 20.7)	18.4(15.6, 21.2)	-0.5(-3.1, 2.0)
Cote d'Ivoire	2016	58.9(56.5, 61.3)	58.3(55.7, 61.0)	59.5(56.6, 62.4)	-1.2(-3.9, 1.6)
Dominican Republic	2013	92.1(90.8, 93.3)	91.0(89.2, 92.8)	93.1(91.5, 94.7)	-2.1(-4.5, 0.2)
Egypt	2014	77.3(75.8, 78.8)	75.4(73.4, 77.4)	79.3(77.7, 80.9)	-3.8(-6.0, -1.7)
El Salvador	2014	84.0(82.7, 85.3)	82.5(80.8, 84.3)	85.5(83.9, 87.1)	-2.9(-5.1, -0.8)
Ethiopia	2016	52.6(50.1, 55.0)	50.9(48.1, 53.8)	54.2(51.3, 57.2)	-3.3(-6.4, -0.2)
Gabon	2012	83.2(80.7, 85.6)	80.6(76.8, 84.4)	85.8(82.7, 88.8)	-5.2(-10.2, -0.1)
Gambia	2013	65.8(62.5, 69.0)	64.5(60.6, 68.3)	67.2(63.5, 70.8)	-2.7(-6.4, 1.0)
Ghana	2014	77.9(75.6, 80.3)	76.5(73.7, 79.2)	79.5(76.5, 82.4)	-3.0(-6.2, 0.2)
Guatemala	2014	49.4(47.6, 51.2)	48.8(46.6, 50.9)	50.0(48.0, 52.1)	-1.3(-3.4, 0.8)
Guinea	2016	58.8(56.9, 60.8)	57.2(54.9, 59.5)	60.6(58.2, 62.9)	-3.4(-6.1, -0.6)
Guinea Bissau	2014	24.7(22.9, 26.5)	24.0(22.1, 25.9)	25.4(22.7, 28.1)	-1.4(-4.4, 1.6)

Guyana	2014	84.6(82.3, 86.9)	84.0(81.1, 86.9)	85.3(82.4, 88.1)	-1.3(-4.7, 2.1)
Haiti	2017	62.3(59.3, 65.3)	62.6(59.5, 65.7)	62.0(58.3, 65.7)	0.6(-2.6, 3.8)
Honduras	2012	68.2(66.5, 69.9)	67.0(65.0, 68.9)	69.5(67.4, 71.6)	-2.5(-4.6, -0.4)
India	2015	57.7(57.7, 57.7)	57.4(57.4, 57.4)	58.1(58.1, 58.1)	-0.7(-0.7, -0.7)
Iraq	2018	87.9(86.7, 89.2)	90.1(88.9, 91.4)	85.6(84.0, 87.2)	4.6(3.0, 6.1)
Jordan	2012	92.2(90.9, 93.4)	90.7(88.9, 92.5)	93.8(92.2, 95.3)	-3.0(-5.4, -0.7)
Kazakhstan	2015	92.0(90.9, 93.0)	92.3(90.7, 93.9)	91.7(90.1, 93.2)	0.6(-1.7, 2.9)
Kenya	2014	51.9(50.3, 53.5)	49.7(47.8, 51.6)	54.2(52.3, 56.1)	-4.5(-6.3, -2.6)
Kosovo	2014	94.2(92.6, 95.8)	94.2(92.3, 96.2)	94.2(92.2, 96.2)	0.0(-2.2, 2.2)
Kyrgyzstan	2014	85.9(84.6, 87.3)	85.0(83.3, 86.7)	86.9(85.1, 88.7)	-1.9(-4.1, 0.4)
Lao	2017	56.0(53.8, 58.1)	55.3(52.9, 57.8)	56.7(54.3, 59.0)	-1.4(-3.4, 0.7)
Lebanon	2011	87.7(87.7, 87.7)	87.7(87.7, 87.7)	87.8(87.8, 87.8)	-0.1(-0.1, -0.1)
Lesotho	2014	32.9(28.3, 37.6)	30.3(25.3, 35.3)	35.1(29.0, 41.3)	-4.9(-11.3, 1.6)
Liberia	2013	46.2(42.8, 49.5)	44.9(40.1, 49.6)	47.7(43.8, 51.6)	-2.8(-8.4, 2.9)
Macedonia	2011	82.7(77.2, 88.1)	83.9(79.5, 88.3)	81.4(73.6, 89.2)	2.5(-4.0, 9.0)
Malawi	2015	15.8(14.2, 17.3)	15.5(13.5, 17.5)	16.0(14.1, 17.9)	-0.5(-2.9, 1.9)
Maldives	2017	82.2(80.0, 84.4)	81.0(77.8, 84.2)	83.4(80.7, 86.2)	-2.4(-6.4, 1.6)
Mali	2015	40.4(38.2, 42.6)	39.3(37.0, 41.6)	41.5(39.1, 43.9)	-2.2(-3.9, -0.5)
Mauritania	2015	67.8(66.0, 69.5)	66.5(64.4, 68.5)	69.1(66.9, 71.2)	-2.6(-4.9, -0.4)
Mexico	2015	85.9(83.9, 88.0)	85.3(82.9, 87.6)	86.5(83.8, 89.2)	-1.3(-4.2, 1.7)
Moldova	2012	93.6(92.3, 94.8)	94.2(92.5, 95.9)	92.9(91.0, 94.9)	1.3(-1.4, 4.0)
Mongolia	2013	88.9(87.9, 89.9)	88.7(87.4, 90.0)	89.1(87.8, 90.4)	-0.4(-2.2, 1.3)
Montenegro	2013	89.0(85.8, 92.3)	87.7(83.8, 91.5)	90.6(86.9, 94.4)	-3.0(-6.8, 0.8)
Mozambique	2011	14.2(12.8, 15.5)	14.5(13.0, 16.0)	13.9(12.3, 15.4)	0.6(-0.9, 2.1)
Myanmar	2016	64.5(61.8, 67.2)	65.0(61.7, 68.3)	64.0(60.4, 67.5)	1.0(-3.3, 5.4)
Namibia	2013	61.4(58.0, 64.7)	59.0(54.5, 63.5)	63.7(59.7, 67.7)	-4.7(-10.0, 0.5)
Nepal	2016	57.9(55.1, 60.6)	57.9(54.5, 61.3)	57.8(54.1, 61.5)	0.1(-4.4, 4.7)
Niger	2012	25.5(23.1, 27.9)	24.2(21.7, 26.8)	26.8(23.9, 29.7)	-2.6(-5.1, -0.1)
Nigeria	2016	30.9(29.3, 32.4)	30.6(28.9, 32.4)	31.1(29.4, 32.8)	-0.5(-2.0, 1.0)
Pakistan	2018	62.0(59.1, 65.0)	61.3(57.8, 64.9)	62.8(59.0, 66.5)	-1.4(-5.8, 2.9)
Palestine	2014	91.5(91.5, 91.5)	90.7(90.7, 90.7)	92.5(92.5, 92.5)	-1.8(-1.8, -1.8)
Paraguay	2016	92.9(91.4, 94.5)	91.8(90.0, 93.6)	94.0(92.3, 95.8)	-2.2(-4.0, -0.4)
Peru	2012	79.9(78.4, 81.5)	79.8(78.0, 81.7)	80.0(78.2, 81.9)	-0.2(-2.2, 1.8)
Rwanda	2015	29.1(27.1, 31.0)	27.3(24.9, 29.6)	30.9(28.3, 33.6)	-3.7(-6.8, -0.6)
STP	2014	59.0(55.2, 62.7)	56.7(52.3, 61.1)	61.2(56.7, 65.7)	-4.5(-9.3, 0.4)
Senegal	2017	57.3(54.4, 60.3)	56.0(52.8, 59.1)	58.8(55.6, 62.0)	-2.8(-5.0, -0.7)
Serbia	2014	93.9(92.2, 95.7)	93.2(90.5, 95.9)	94.8(93.2, 96.3)	-1.5(-4.5, 1.4)
Sierra Leone	2017	43.4(42.5, 44.3)	44.4(43.0, 45.8)	42.3(40.9, 43.7)	2.1(-0.0, 4.3)
South Africa	2016	58.4(53.6, 63.2)	57.3(51.2, 63.4)	59.5(53.2, 65.8)	-2.2(-10.1, 5.7)
St. Lucia	2012	96.6(94.5, 98.7)	95.6(91.9, 99.4)	97.6(95.4, 99.7)	-2.0(-6.4, 2.5)

Sudan	2014	54.0(52.1, 55.9)	51.5(49.3, 53.7)	56.6(54.4, 58.8)	-5.1(-7.4, -2.9)
Suriname	2010	92.7(91.2, 94.1)	92.3(90.3, 94.3)	93.0(90.9, 95.1)	-0.7(-3.6, 2.2)
Swaziland	2014	47.0(43.5, 50.6)	43.4(39.2, 47.5)	50.8(46.9, 54.6)	-7.4(-11.2, -3.5)
Tajikistan	2017	79.7(78.0, 81.3)	79.5(77.3, 81.7)	79.8(77.9, 81.7)	-0.3(-2.7, 2.1)
Tanzania	2015	37.2(34.8, 39.6)	36.2(33.6, 38.7)	38.2(35.6, 40.9)	-2.1(-4.3, 0.1)
Thailand	2016	89.5(88.2, 90.8)	88.5(86.4, 90.6)	90.6(89.0, 92.1)	-2.1(-4.7, 0.5)
Timor-Leste	2016	40.3(38.3, 42.3)	38.6(36.3, 41.0)	42.1(39.5, 44.6)	-3.4(-6.4, -0.4)
Togo	2014	42.9(39.5, 46.3)	43.5(39.8, 47.2)	42.3(38.1, 46.4)	1.2(-2.8, 5.2)
Tunisia	2012	89.1(87.5, 90.6)	87.7(85.6, 89.8)	90.6(88.8, 92.4)	-2.9(-5.3, -0.5)
Turkmenistan	2015	81.9(79.3, 84.4)	82.2(79.3, 85.2)	81.5(78.6, 84.5)	0.7(-2.2, 3.6)
Uganda	2016	44.5(42.0, 47.0)	43.8(40.7, 46.9)	45.1(42.2, 48.0)	-1.3(-4.7, 2.1)
Vietnam	2011	75.2(73.0, 77.4)	74.6(71.9, 77.4)	75.7(72.9, 78.5)	-1.1(-4.5, 2.3)
Yemen	2013	44.9(43.0, 46.8)	44.1(42.0, 46.2)	45.7(43.5, 47.9)	-1.5(-3.5, 0.4)
Zambia	2013	22.1(20.7, 23.5)	21.8(20.2, 23.4)	22.4(20.6, 24.1)	-0.5(-2.4, 1.3)
Zimbabwe	2015	54.1(51.5, 56.7)	51.1(48.2, 54.1)	56.9(53.8, 60.0)	-5.8(-8.9, -2.6)

*: Gender gaps are defined as the difference between boy and girl averages. A negative gender gap implies a girl advantage, a positive gender gap a boy advantage.

Table 2. Prevalence (%) of children NOT stunted or in extreme poverty by place of residence with 95% confidence intervals, the most recent years in 85 countries

Country	Year	Urban	Rural	Difference
Albania	2017	87.5(84.5, 90.5)	84.6(80.9, 88.2)	-2.9(-7.7, 1.8)
Algeria	2012	88.7(87.0, 90.4)	87.3(86.2, 88.4)	-1.4(-3.5, 0.7)
Angola	2015	67.0(64.0, 70.0)	20.6(17.2, 23.9)	-46.4(-50.9, -41.9)
Armenia	2016	92.7(89.2, 96.1)	85.7(83.8, 87.6)	-7.0(-10.9, -3.0)
Bangladesh	2014	65.7(62.9, 68.5)	49.9(46.2, 53.5)	-15.8(-20.4, -11.2)
Belize	2015	89.2(85.8, 92.6)	78.3(75.7, 80.8)	-10.9(-15.2, -6.7)
Benin	2014	63.2(63.2, 63.2)	47.8(47.8, 47.8)	-15.4(-15.4, -15.4)
Bhutan	2010	72.0(72.0, 72.0)	60.0(60.0, 60.0)	-12.0(-12.0, -12.0)
Bosnia and Herzegovina	2011	89.1(89.1, 89.1)	92.1(92.1, 92.1)	2.9(2.9, 2.9)
Burkina Faso	2010	72.4(70.5, 74.4)	21.0(17.4, 24.7)	-51.4(-55.6, -47.3)
Burundi	2017	68.2(66.7, 69.6)	9.3(2.8, 15.7)	-58.9(-65.6, -52.2)
Cambodia	2014	74.8(71.4, 78.1)	41.4(37.8, 45.0)	-33.4(-38.3, -28.5)
Cameroon	2014	74.3(71.4, 77.2)	29.7(27.4, 32.0)	-44.7(-48.4, -41.0)

Central African Republic	2010	49.4(47.9, 50.9)	9.4(6.3, 12.5)	-40.0(-43.5, -36.5)
Chad	2015	62.6(60.1, 65.1)	37.1(33.4, 40.7)	-25.5(-30.0, -21.0)
Colombia	2010	87.8(85.0, 90.7)	67.0(66.1, 67.9)	-20.9(-23.9, -17.9)
Comoros	2012	68.3(64.1, 72.5)	53.8(48.5, 59.1)	-14.5(-21.2, -7.7)
Congo	2015	82.9(79.6, 86.2)	44.2(42.1, 46.3)	-38.7(-42.6, -34.8)
Congo DR	2013	53.3(52.1, 54.5)	2.7(-1.6, 6.9)	-50.7(-55.1, -46.2)
Cote d'Ivoire	2016	86.5(83.2, 89.8)	41.3(39.4, 43.3)	-45.2(-49.0, -41.3)
Dominican Republic	2013	92.7(89.7, 95.8)	90.1(88.8, 91.4)	-2.6(-6.0, 0.7)
Egypt	2014	76.4(74.6, 78.2)	77.7(75.0, 80.4)	1.3(-2.0, 4.5)
El Salvador	2014	87.9(85.6, 90.1)	78.8(77.4, 80.3)	-9.0(-11.7, -6.3)
Ethiopia	2016	72.3(69.6, 74.9)	50.1(44.9, 55.4)	-22.1(-28.0, -16.2)
Gabon	2012	86.1(82.3, 89.9)	67.7(64.9, 70.5)	-18.4(-23.2, -13.7)
Gambia	2013	78.4(73.8, 83.0)	55.8(52.4, 59.1)	-22.6(-28.4, -16.9)
Ghana	2014	86.2(82.6, 89.7)	71.0(68.5, 73.5)	-15.2(-19.5, -10.8)
Guatemala	2014	63.7(61.5, 66.0)	41.4(38.4, 44.5)	-22.3(-26.1, -18.5)
Guinea	2016	78.5(76.0, 81.1)	48.7(46.9, 50.5)	-29.9(-33.0, -26.7)
Guinea Bissau	2014	59.3(58.2, 60.4)	5.0(1.6, 8.5)	-54.2(-57.8, -50.6)
Guyana	2014	90.1(87.4, 92.8)	82.9(78.7, 87.1)	-7.2(-12.2, -2.1)
Haiti	2017	81.9(77.7, 86.1)	52.4(49.9, 55.0)	-29.4(-34.4, -24.5)
Honduras	2012	84.2(81.7, 86.7)	55.0(53.2, 56.8)	-29.2(-32.3, -26.1)
India	2015	68.1(68.1, 68.1)	53.8(53.8, 53.8)	-14.3(-14.3, -14.3)
Iraq	2018	89.7(86.5, 92.9)	84.2(82.9, 85.5)	-5.5(-9.0, -2.0)
Jordan	2012	92.5(91.1, 94.0)	90.8(89.3, 92.3)	-1.7(-3.8, 0.4)
Kazakhstan	2015	92.8(91.2, 94.5)	91.1(89.8, 92.5)	-1.7(-3.8, 0.4)
Kenya	2014	73.6(71.7, 75.4)	40.6(38.3, 42.9)	-32.9(-35.9, -30.0)
Kosovo	2014	95.6(93.5, 97.6)	93.5(90.8, 96.1)	-2.1(-5.5, 1.3)
Kyrgyzstan	2014	87.6(85.9, 89.3)	85.2(83.2, 87.2)	-2.4(-5.0, 0.3)
Lao	2017	77.2(74.7, 79.8)	48.2(45.8, 50.6)	-29.0(-32.5, -25.5)
Lebanon	2011	88.0(88.0, 88.0)	87.1(87.1, 87.1)	-0.9(-0.9, -0.9)
Lesotho	2014	66.2(62.2, 70.1)	20.4(12.1, 28.6)	-45.8(-55.0, -36.6)

Liberia	2013	64.6(61.0, 68.2)	27.3(22.7, 32.0)	-37.3(-43.2, -31.4)
Macedonia	2011	92.4(82.6, 102.1)	72.8(69.8, 75.7)	-19.6(-29.8, -9.4)
Malawi	2015	60.8(59.4, 62.2)	9.0(3.9, 14.2)	-51.7(-57.1, -46.4)
Maldives	2017	86.5(84.5, 88.5)	80.3(74.8, 85.8)	-6.2(-12.1, -0.3)
Mali	2015	76.7(74.3, 79.2)	31.4(26.2, 36.5)	-45.4(-51.1, -39.6)
Mauritania	2015	76.6(74.1, 79.0)	61.0(58.9, 63.1)	-15.6(-18.9, -12.4)
Mexico	2015	89.7(84.9, 94.6)	74.6(72.7, 76.5)	-15.1(-20.4, -9.9)
Moldova	2012	96.7(94.9, 98.4)	92.0(90.7, 93.3)	-4.7(-6.9, -2.4)
Mongolia	2013	91.6(89.7, 93.5)	84.7(83.7, 85.8)	-6.8(-9.0, -4.6)
Montenegro	2013	89.3(83.6, 95.0)	88.7(84.6, 92.7)	-0.6(-7.6, 6.4)
Mozambique	2011	40.4(39.4, 41.5)	4.5(0.4, 8.7)	-35.9(-40.2, -31.6)
Myanmar	2016	79.7(76.7, 82.7)	59.9(55.8, 63.9)	-19.8(-24.9, -14.8)
Namibia	2013	81.3(76.9, 85.6)	46.1(41.8, 50.4)	-35.1(-41.3, -29.0)
Nepal	2016	63.9(59.7, 68.2)	51.0(47.3, 54.8)	-12.9(-18.7, -7.2)
Niger	2012	69.0(66.6, 71.3)	19.1(15.0, 23.2)	-49.9(-54.6, -45.1)
Nigeria	2016	60.9(59.4, 62.5)	17.6(14.4, 20.8)	-43.4(-47.0, -39.7)
Pakistan	2018	68.5(64.5, 72.5)	58.9(55.2, 62.5)	-9.6(-15.1, -4.2)
Palestine	2014	91.4(91.4, 91.4)	92.1(92.1, 92.1)	0.6(0.6, 0.6)
Paraguay	2016	95.6(92.3, 98.9)	88.9(87.7, 90.1)	-6.7(-10.2, -3.2)
Peru	2012	89.5(86.4, 92.6)	60.9(59.7, 62.2)	-28.6(-32.0, -25.2)
Rwanda	2015	68.1(66.1, 70.1)	21.4(16.5, 26.2)	-46.7(-52.0, -41.4)
STP	2014	63.9(57.7, 70.1)	49.5(44.9, 54.1)	-14.4(-22.2, -6.6)
Senegal	2017	86.0(81.9, 90.1)	40.9(39.1, 42.7)	-45.1(-49.6, -40.6)
Serbia	2014	93.5(91.3, 95.6)	94.7(92.3, 97.1)	1.2(-2.0, 4.5)
Sierra Leone	2017	78.3(77.3, 79.3)	22.9(21.3, 24.5)	-55.4(-57.3, -53.4)
South Africa	2016	67.1(60.0, 74.2)	47.0(40.4, 53.6)	-20.1(-29.8, -10.4)
St. Lucia	2012	91.5(89.4, 93.6)	97.8(91.4, 104.2)	6.3(-0.6, 13.1)
Sudan	2014	71.4(69.1, 73.8)	46.7(43.9, 49.5)	-24.7(-28.5, -21.0)
Suriname	2010	93.2(90.9, 95.4)	91.0(89.2, 92.7)	-2.2(-5.0, 0.7)
Swaziland	2014	77.3(73.6, 81.0)	38.4(32.5, 44.3)	-38.9(-45.9, -31.9)

Tajikistan	2017	81.1(79.2, 83.1)	79.3(76.8, 81.8)	-1.9(-5.1, 1.3)
Tanzania	2015	70.0(67.5, 72.4)	25.7(21.0, 30.5)	-44.2(-49.5, -38.9)
Thailand	2016	90.2(88.5, 92.0)	89.0(87.0, 91.1)	-1.2(-3.9, 1.5)
Timor-Leste	2016	57.5(55.1, 59.8)	33.6(29.4, 37.9)	-23.8(-28.7, -19.0)
Togo	2014	84.0(80.5, 87.6)	21.1(18.4, 23.9)	-62.9(-67.4, -58.4)
Tunisia	2012	91.6(88.8, 94.5)	84.7(82.9, 86.5)	-6.9(-10.3, -3.5)
Turkmenistan	2015	86.2(82.5, 89.8)	79.6(76.9, 82.3)	-6.6(-11.1, -2.0)
Uganda	2016	69.2(66.5, 71.9)	38.2(33.5, 42.8)	-31.0(-36.4, -25.6)
Vietnam	2011	88.0(85.2, 90.8)	70.3(67.8, 72.8)	-17.7(-21.5, -14.0)
Yemen	2013	66.1(63.9, 68.4)	36.9(34.3, 39.5)	-29.2(-32.7, -25.7)
Zambia	2013	53.8(52.9, 54.7)	6.3(3.5, 9.2)	-47.4(-50.4, -44.5)
Zimbabwe	2015	77.6(74.3, 80.9)	44.1(41.2, 47.0)	-33.5(-37.9, -29.1)

*: Gaps by residential area are defined as the difference between rural and urban averages. A negative area gap implies urban advantage, a positive area gap rural advantage.

Table 3. Level and trends of national prevalence (%) of children NOT stunted or in extreme poverty with 95% confidence intervals in 40 countries with data available in two rounds

Country	Round 1	Round 2	Round 1-Round 2
Armenia	79.9(77.0, 82.8)	89.6(87.7, 91.5)	-9.7(-13.2, -6.2)
Bangladesh	47.4(45.4, 49.5)	53.8(51.5, 56.2)	-6.4(-9.6, -3.2)
Belize	75.2(72.2, 78.2)	82.4(80.1, 84.7)	-7.2(-11.0, -3.4)
Benin	31.1(29.1, 33.0)	54.5(52.4, 56.6)	-23.4(-26.3, -20.6)
Burundi	9.2(7.9, 10.5)	14.5(12.7, 16.3)	-5.4(-7.6, -3.1)
Cambodia	32.3(29.4, 35.1)	46.1(43.0, 49.3)	-13.9(-18.1, -9.6)
Cameroon	51.6(48.6, 54.5)	48.1(45.0, 51.2)	3.4(-0.5, 7.4)
Chad	33.4(31.1, 35.8)	42.0(39.8, 44.1)	-8.5(-11.7, -5.4)
Congo	62.8(60.0, 65.6)	68.3(66.3, 70.4)	-5.5(-8.9, -2.1)
Congo DR	10.2(8.5, 11.9)	18.1(15.6, 20.7)	-7.9(-11.0, -4.8)
Cote d'Ivoire	49.2(45.6, 52.9)	58.9(56.5, 61.4)	-9.7(-14.1, -5.3)
Ethiopia	39.5(37.0, 42.0)	52.6(50.0, 55.1)	-13.1(-16.7, -9.5)
Gambia	63.4(61.4, 65.5)	65.8(62.5, 69.1)	-2.4(-6.2, 1.5)
Ghana	73.9(72.2, 75.7)	77.9(75.3, 80.6)	-4.0(-7.2, -0.7)
Guinea	42.9(39.5, 46.2)	58.8(56.9, 60.8)	-16.0(-19.8, -12.1)
Haiti	63.1(59.7, 66.5)	62.3(59.1, 65.5)	0.8(-4.0, 5.6)
Iraq	77.1(76.2, 77.9)	87.9(86.7, 89.2)	-10.9(-12.4, -9.4)
Kazakhstan	86.9(85.6, 88.1)	92.0(90.9, 93.0)	-5.1(-6.7, -3.4)
Kyrgyzstan	79.9(77.8, 82.1)	85.9(84.6, 87.3)	-6.0(-8.5, -3.5)
Lao	44.1(41.8, 46.4)	56.0(53.8, 58.1)	-11.8(-14.8, -8.8)
Malawi	17.9(15.7, 20.1)	15.8(14.2, 17.3)	2.1(-0.5, 4.8)
Mali	36.1(34.0, 38.3)	40.4(37.8, 42.9)	-4.2(-7.8, -0.7)
Mauritania	64.6(63.0, 66.2)	67.8(65.9, 69.6)	-3.2(-5.7, -0.7)
Mongolia	84.2(82.7, 85.7)	88.9(87.9, 89.9)	-4.7(-6.5, -2.9)
Nepal	52.2(48.5, 55.9)	57.9(55.0, 60.7)	-5.7(-10.3, -1.0)
Nigeria	40.6(38.1, 43.0)	30.9(29.3, 32.4)	9.7(6.8, 12.6)
Pakistan	52.5(48.8, 56.1)	62.0(59.0, 65.1)	-9.5(-14.3, -4.8)
Palestine	88.8(88.1, 89.5)	91.5(90.8, 92.2)	-2.7(-3.7, -1.7)
Peru	74.0(72.2, 75.8)	79.9(78.3, 81.6)	-5.9(-8.6, -3.3)
Rwanda	21.9(20.0, 23.9)	29.1(26.8, 31.4)	-7.1(-10.3, -4.0)
Senegal	53.2(49.3, 57.1)	57.3(53.7, 61.0)	-4.1(-10.0, 1.8)
Serbia	93.4(91.9, 94.8)	93.9(92.2, 95.7)	-0.6(-2.9, 1.7)
Sierra Leone	24.7(23.6, 25.7)	43.4(42.5, 44.3)	-18.7(-20.1, -17.3)
Swaziland	38.0(35.1, 41.0)	47.0(43.3, 50.8)	-9.0(-13.6, -4.3)
Tajikistan	54.2(50.9, 57.5)	79.7(78.0, 81.3)	-25.5(-29.0, -21.9)

Tanzania	25.9(23.1, 28.6)	37.2(34.8, 39.6)	-11.3(-15.0, -7.7)
Thailand	83.6(81.9, 85.4)	89.5(88.2, 90.8)	-5.9(-8.0, -3.7)
Togo	38.3(35.4, 41.3)	42.9(39.4, 46.3)	-4.5(-9.1, -0.0)
Uganda	40.9(37.3, 44.6)	44.5(41.6, 47.3)	-3.5(-8.0, 1.0)
Zimbabwe	53.2(50.9, 55.6)	54.1(51.2, 56.9)	-0.8(-4.5, 2.8)

*: A negative sign implies an increase of percentage, a positive sign a decrease of percentage.

Lu C, *et al. BMJ Global Health* 2020; 5:e002314. doi: 10.1136/bmjgh-2020-002314

Table 4. Level and trends of gender inequalities in the prevalence (%) of children NOT stunted or in extreme poverty with 95% confidence intervals in 40 countries with data available in two rounds

Country	Round 1			Round 2			Round1 – Round2
	Male	Female	Gap	Male	Female	Gap	
Armenia	78.7	81.2	-2.4(-7.5, 2.7)	88.3	91.1	-2.8(-6.0, 0.4)	0.4(-5.7, 6.4)
Bangladesh	48.4	46.4	2.0(-0.6, 4.7)	53.7	54.0	-0.4(-3.2, 2.4)	-1.6(-5.5, 2.3)
Belize	76.8	73.5	3.3(-0.7, 7.4)	81.0	83.9	-2.9(-6.3, 0.6)	-0.4(-5.8, 4.9)
Benin	30.5	31.6	-1.0(-3.3, 1.2)	53.0	55.9	-2.9(-5.1, -0.7)	1.8(-1.3, 5.0)
Burundi	7.7	10.7	-3.0(-5.1, -0.9)	14.0	15.1	-1.1(-2.9, 0.7)	-1.9(-4.7, 0.8)
Cambodia	29.7	35.0	-5.3(-8.9, -1.8)	46.5	45.8	0.6(-3.2, 4.4)	-4.7(-9.9, 0.5)
Cameroon	49.6	53.5	-3.9(-7.1, -0.8)	47.2	49.1	-1.9(-4.9, 1.1)	-2.1(-6.4, 2.3)
Chad	33.2	33.7	-0.5(-2.6, 1.7)	41.9	42.0	-0.1(-2.5, 2.3)	-0.4(-3.6, 2.8)
Congo	63.3	62.3	1.0(-3.6, 5.6)	67.8	68.8	-1.0(-3.6, 1.5)	0.0(-5.2, 5.3)
Congo DR	9.3	11.1	-1.9(-3.2, -0.5)	17.9	18.4	-0.5(-3.1, 2.0)	-1.3(-4.2, 1.6)
Cote d'Ivoire	48.5	49.9	-1.3(-5.7, 3.0)	58.3	59.5	-1.2(-3.9, 1.6)	-0.2(-5.3, 4.9)
Ethiopia	38.4	40.6	-2.2(-4.9, 0.6)	50.9	54.2	-3.3(-6.4, -0.2)	1.1(-3.0, 5.3)
Gambia	61.4	65.5	-4.1(-6.4, -1.8)	64.5	67.2	-2.7(-6.4, 1.0)	-1.4(-5.7, 3.0)
Ghana	71.3	76.5	-5.3(-8.1, -2.4)	76.5	79.5	-3.0(-6.2, 0.2)	-2.3(-6.5, 2.0)
Guinea	42.4	43.3	-0.9(-4.9, 3.2)	57.2	60.6	-3.4(-6.1, -0.6)	2.5(-2.4, 7.4)
Haiti	62.5	63.8	-1.3(-4.5, 1.9)	62.6	62.0	0.6(-2.6, 3.8)	-0.7(-5.3, 3.8)
Iraq	76.5	77.7	-1.2(-2.6, 0.2)	90.1	85.6	4.6(3.0, 6.1)	3.3(1.3, 5.4)
Kazakhstan	86.8	87.0	-0.2(-2.4, 2.0)	92.3	91.7	0.6(-1.7, 2.9)	0.4(-2.8, 3.5)
Kyrgyzstan	77.6	82.4	-4.8(-7.8, -1.8)	85.0	86.9	-1.9(-4.1, 0.4)	-3.0(-6.8, 0.8)
Lao	42.5	45.8	-3.3(-5.5, -1.1)	55.3	56.7	-1.4(-3.4, 0.7)	-1.9(-5.0, 1.1)
Malawi	16.8	18.9	-2.1(-5.4, 1.2)	15.5	16.0	-0.5(-2.9, 1.9)	-1.6(-5.7, 2.5)
Mali	34.9	37.5	-2.6(-4.1, -1.2)	39.3	41.5	-2.2(-3.9, -0.5)	-0.4(-2.7, 1.8)
Mauritania	63.2	66.0	-2.7(-5.1, -0.4)	66.5	69.1	-2.6(-4.9, -0.4)	-0.1(-3.3, 3.1)
Mongolia	82.4	86.1	-3.7(-6.1, -1.3)	88.7	89.1	-0.4(-2.2, 1.3)	-3.2(-6.2, -0.3)
Nepal	51.8	52.6	-0.8(-5.1, 3.5)	57.9	57.8	0.1(-4.4, 4.6)	-0.7(-6.9, 5.6)
Nigeria	40.7	40.5	0.2(-1.5, 1.8)	30.6	31.1	-0.5(-2.0, 1.0)	0.3(-1.9, 2.6)
Pakistan	50.2	54.9	-4.7(-9.2, -0.2)	61.3	62.8	-1.4(-5.7, 2.9)	-3.3(-9.5, 2.9)
Palestine	87.9	89.8	-1.9(-3.3, -0.5)	90.7	92.5	-1.8(-3.2, -0.4)	-0.1(-2.1, 1.9)
Peru	72.5	75.5	-3.1(-5.2, -0.9)	79.8	80.0	-0.2(-2.2, 1.8)	-2.9(-5.8, 0.1)
Rwanda	21.7	22.2	-0.5(-3.2, 2.1)	27.3	30.9	-3.7(-6.8, -0.6)	3.2(-0.9, 7.2)
Senegal	51.6	54.9	-3.4(-7.9, 1.2)	56.0	58.8	-2.8(-5.0, -0.7)	-0.5(-5.5, 4.5)
Serbia	94.0	92.7	1.3(-1.3, 3.9)	93.2	94.8	-1.5(-4.5, 1.4)	0.2(-3.7, 4.2)
Sierra Leone	23.4	26.0	-2.6(-4.8, -0.4)	44.4	42.3	2.1(-0.0, 4.3)	-0.4(-3.5, 2.6)
Swaziland	37.6	38.5	-0.9(-4.8, 3.0)	43.4	50.8	-7.4(-11.2, -3.5)	6.5(0.9, 12.1)

Tajikistan	54.2	54.2	0.0(-3.3, 3.3)	79.5	79.8	-0.3(-2.6, 2.1)	0.3(-3.9, 4.5)
Tanzania	24.2	27.5	-3.4(-5.7, -1.0)	36.2	38.2	-2.1(-4.3, 0.1)	-1.3(-4.5, 2.0)
Thailand	83.6	83.7	-0.1(-3.0, 2.9)	88.5	90.6	-2.1(-4.7, 0.5)	2.0(-1.9, 5.9)
Togo	36.7	40.1	-3.4(-6.9, 0.0)	43.5	42.3	1.2(-2.8, 5.2)	-2.2(-7.5, 3.0)
Uganda	39.5	42.3	-2.8(-8.0, 2.4)	43.8	45.1	-1.3(-4.7, 2.1)	-1.5(-7.7, 4.7)
Zimbabwe	50.5	56.0	-5.5(-8.7, -2.4)	51.1	56.9	-5.8(-8.9, -2.6)	0.2(-4.2, 4.7)

*: Gender gaps are defined as the difference between boy and girl averages. A positive sign implies an increase of inequality, a negative sign implies a reduction of inequality.

Table 5. Level and trends of area inequalities in the prevalence (%) of children NOT stunted or in extreme poverty with 95% confidence intervals in 40 countries with data available in two rounds

**Country**	**Round 1**			**Round 2**			**Round1 – Round2**
	Rural	Urban	Gap	Rural	Urban	Gap	
Armenia	76.6	82.3	-5.7(-11.6, 0.2)	85.7	92.7	-7.0(-10.9, -3.0)	1.3(-5.8, 8.4)
Bangladesh	43.7	60.6	-16.9(-20.9, -12.8)	49.9	65.7	-15.8(-20.4, -11.2)	-1.0(-7.1, 5.1)
Belize	70.1	83.6	-13.5(-19.6, -7.4)	78.3	89.2	-10.9(-15.2, -6.7)	-2.6(-10.0, 4.8)
Benin	19.8	48.5	-28.7(-32.6, -24.8)	47.8	63.2	-15.4(-19.5, -11.3)	-13.3(-18.9, -7.7)
Burundi	5.0	55.6	-50.6(-58.7, -42.4)	9.3	68.2	-58.9(-65.6, -52.2)	8.3(-2.2, 18.9)
Cambodia	25.8	67.4	-41.5(-46.9, -36.1)	41.4	74.8	-33.4(-38.4, -28.4)	-8.1(-15.4, -0.8)
Cameroon	31.2	78.3	-47.1(-51.0, -43.1)	29.7	74.3	-44.7(-49.2, -40.2)	-2.4(-8.5, 3.7)
Chad	24.4	64.6	-40.2(-43.8, -36.5)	37.1	62.6	-25.5(-30.1, -20.9)	-14.7(-20.5, -8.8)
Congo	36.7	80.6	-43.9(-48.9, -38.9)	44.2	82.9	-38.7(-42.6, -34.8)	-5.2(-11.6, 1.2)
Congo DR	0.8	36.1	-35.3(-40.7, -30.0)	2.7	53.3	-50.7(-55.1, -46.3)	15.3(8.4, 22.3)
Cote d'Ivoire	32.4	79.6	-47.1(-52.4, -41.9)	41.3	86.5	-45.2(-49.0, -41.3)	-1.9(-8.4, 4.6)
Ethiopia	35.6	66.5	-30.9(-37.2, -24.5)	50.1	72.3	-22.1(-28.4, -15.8)	-8.7(-17.9, 0.5)
Gambia	50.1	81.3	-31.2(-35.0, -27.4)	55.8	78.4	-22.6(-28.4, -16.9)	-8.6(-15.5, -1.7)
Ghana	67.9	81.8	-13.8(-17.3, -10.4)	71.0	86.2	-15.2(-19.9, -10.4)	1.3(-4.6, 7.3)
Guinea	30.2	81.1	-50.9(-55.9, -45.9)	48.7	78.5	-29.9(-33.0, -26.8)	-21.1(-26.9, -15.2)
Haiti	52.6	83.9	-31.3(-36.7, -25.9)	52.4	81.9	-29.4(-34.4, -24.5)	-1.9(-9.2, 5.5)
Iraq	73.0	79.1	-6.1(-7.7, -4.4)	84.2	89.7	-5.5(-9.0, -2.0)	-0.5(-4.4, 3.3)
Kazakhstan	86.6	87.2	-0.6(-3.1, 1.8)	91.1	92.8	-1.7(-3.8, 0.4)	1.0(-2.3, 4.3)
Kyrgyzstan	81.4	76.3	5.1(-0.3, 10.5)	85.2	87.6	-2.4(-5.0, 0.3)	-2.7(-8.7, 3.3)
Lao	37.2	70.3	-33.1(-37.2, -29.0)	48.2	77.2	-29.0(-32.5, -25.5)	-4.0(-9.4, 1.3)
Malawi	12.1	50.6	-38.6(-45.0, -32.2)	9.0	60.8	-51.7(-57.1, -46.4)	13.2(4.8, 21.5)
Mali	23.9	74.7	-50.9(-54.1, -47.6)	31.4	76.7	-45.4(-51.2, -39.6)	-5.5(-12.2, 1.2)
Mauritania	58.0	74.7	-16.7(-19.7, -13.7)	61.0	76.6	-15.6(-18.9, -12.3)	-1.1(-5.6, 3.4)
Mongolia	78.9	88.1	-9.2(-12.1, -6.2)	84.7	91.6	-6.8(-9.0, -4.6)	-2.3(-6.0, 1.3)
Nepal	50.5	70.2	-19.7(-26.4, -13.0)	51.0	63.9	-12.9(-18.7, -7.1)	-6.8(-15.6, 2.0)
Nigeria	27.4	70.7	-43.2(-48.4, -38.1)	17.6	60.9	-43.4(-47.0, -39.7)	0.1(-6.2, 6.4)
Pakistan	47.8	63.0	-15.2(-21.5, -9.0)	58.9	68.5	-9.6(-15.2, -4.1)	-5.6(-13.9, 2.7)
Palestine	87.9	89.0	-1.1(-3.0, 0.8)	92.1	91.4	0.6(-1.2, 2.4)	-0.5(-3.1, 2.1)
Peru	53.9	85.4	-31.4(-34.7, -28.1)	60.9	89.5	-28.6(-32.0, -25.2)	-2.9(-7.6, 1.9)
Rwanda	16.8	60.9	-44.1(-52.0, -36.1)	21.4	68.1	-46.7(-52.2, -41.2)	2.6(-7.1, 12.4)
Senegal	37.2	77.8	-40.6(-46.6, -34.6)	40.9	86.0	-45.1(-50.3, -39.9)	4.5(-3.6, 12.5)
Serbia	91.9	94.5	-2.5(-5.6, 0.5)	94.7	93.5	1.2(-2.0, 4.5)	-1.3(-5.7, 3.1)
Sierra Leone	18.2	41.9	-23.7(-26.2, -21.3)	22.9	78.3	-55.4(-57.3, -53.4)	31.6(28.5, 34.8)
Swaziland	30.2	69.9	-39.7(-45.5, -34.0)	38.4	77.3	-38.9(-45.8, -31.9)	-0.9(-9.7, 7.9)

Tajikistan	48.6	74.7	-26.1(-31.4, -20.7)	79.3	81.1	-1.9(-5.1, 1.3)	-24.2(-30.3, -18.1)
Tanzania	16.6	64.1	-47.5(-53.2, -41.9)	25.7	70.0	-44.2(-49.6, -38.8)	-3.3(-11.1, 4.5)
Thailand	81.8	86.6	-4.8(-7.8, -1.7)	89.0	90.2	-1.2(-3.9, 1.5)	-3.5(-7.6, 0.5)
Togo	18.5	80.1	-61.6(-65.9, -57.3)	21.1	84.0	-62.9(-67.4, -58.4)	1.3(-4.9, 7.4)
Uganda	35.1	77.7	-42.6(-50.0, -35.2)	38.2	69.2	-31.0(-36.9, -25.2)	-11.6(-20.8, -2.3)
Zimbabwe	46.4	72.3	-25.9(-29.8, -21.9)	44.1	77.6	-33.5(-37.9, -29.1)	7.6(1.7, 13.5)

*: Gaps by residential area are defined as the difference between rural and urban averages. A positive sign implies an increase of inequality, a negative sign implies a reduction of inequality.

(2) Attendance of early care and early education

Table 6. Prevalence (%) of children attending early care and education by gender with 95% confidence intervals, the most recent years in 65 countries

Country	National	Male	Female	Difference*
Afghanistan	1.1(0.8, 1.3)	1.0(0.7, 1.3)	1.1(0.8, 1.5)	-0.1(-0.6, 0.4)
Algeria	18.3(16.8, 19.8)	17.1(15.2, 18.9)	16.4(14.5, 18.2)	0.7(-1.9, 3.4)
Argentina	63.8(61.1, 66.4)	32.0(29.7, 34.2)	32.2(29.8, 34.5)	-0.2(-3.5, 3.1)
Bangladesh	13.9(12.9, 14.8)	13.1(11.9, 14.3)	13.9(12.5, 15.2)	-0.8(-2.6, 1.0)
Belarus	88.0(85.6, 90.3)	86.6(83.1, 90.0)	89.3(86.3, 92.3)	-2.7(-7.3, 1.8)
Belize	55.6(52.0, 59.3)	53.2(48.3, 58.1)	59.0(53.8, 64.1)	-5.8(-12.9, 1.3)
Benin	13.1(12.0, 14.2)	12.8(11.2, 14.4)	13.7(12.1, 15.3)	-0.9(-3.1, 1.4)
Bhutan	9.8(8.2, 11.4)	9.6(7.4, 11.8)	9.5(7.4, 11.6)	0.1(-2.9, 3.2)
Bosnia and Herzegovina	12.7(9.7, 15.8)	12.2(7.5, 16.9)	14.0(9.7, 18.3)	-1.8(-8.1, 4.6)
Burundi	6.7(6.3, 7.2)	6.7(6.1, 7.3)	6.8(6.1, 7.4)	-0.1(-1.0, 0.8)
Cameroon	27.9(26.0, 29.8)	27.0(24.4, 29.5)	29.2(26.6, 31.9)	-2.3(-5.9, 1.4)
Central African Republic	4.5(3.6, 5.5)	4.6(3.2, 6.0)	5.5(4.1, 6.9)	-0.9(-2.9, 1.0)
Chad	2.5(2.0, 2.9)	2.9(2.1, 3.6)	2.9(2.3, 3.5)	-0.0(-1.1, 1.0)
Congo, Dem. Rep.	6.6(5.4, 7.8)	6.7(5.0, 8.5)	6.6(5.0, 8.2)	0.1(-2.2, 2.5)
Congo, Rep.	37.2(34.7, 39.6)	36.6(33.3, 39.9)	37.3(33.7, 40.8)	-0.7(-5.5, 4.2)
Costa Rica	15.9(11.7, 20.1)	16.7(9.6, 23.7)	17.7(12.0, 23.4)	-1.0(-10.1, 8.1)
Cote d'Ivoire	14.3(12.7, 16.0)	14.1(11.9, 16.3)	15.0(12.6, 17.4)	-0.9(-4.2, 2.3)
Dominican Republic	40.9(39.1, 42.7)	41.0(38.6, 43.4)	41.7(39.2, 44.1)	-0.6(-4.1, 2.8)
El Salvador	25.1(23.0, 27.1)	24.6(21.7, 27.4)	26.1(23.2, 29.1)	-1.6(-5.7, 2.5)
Gambia	17.9(16.2, 19.6)	6.4(5.6, 7.3)	7.2(6.2, 8.2)	-0.8(-2.1, 0.6)
Ghana	67.9(65.5, 70.3)	65.0(61.6, 68.5)	71.7(68.6, 74.8)	-6.7(-11.3, -2.0)
Guinea	9.4(8.2, 10.6)	8.9(7.3, 10.5)	9.5(7.8, 11.1)	-0.6(-2.9, 1.7)
Guinea-Bissau	12.9(11.1, 14.7)	12.9(10.5, 15.2)	13.6(11.2, 16.1)	-0.8(-4.2, 2.7)
Guyana	62.3(59.4, 65.2)	64.7(60.9, 68.4)	60.1(56.1, 64.1)	4.6(-0.9, 10.0)
Iraq	86.9(80.8, 92.9)	84.8(75.1, 94.4)	88.9(81.8, 96.0)	-4.1(-16.2, 7.9)
Jamaica	92.1(89.4, 94.8)	92.9(89.5, 96.3)	91.7(88.0, 95.4)	1.2(-3.9, 6.3)
Jordan	21.0(19.3, 22.7)	20.8(18.4, 23.1)	21.0(18.5, 23.5)	-0.2(-3.6, 3.2)
Kazakhstan	56.9(53.6, 60.2)	54.0(49.6, 58.4)	58.8(54.1, 63.5)	-4.8(-11.2, 1.6)
Kosovo	13.8(11.0, 16.6)	14.5(10.6, 18.3)	13.9(9.9, 18.0)	0.5(-5.1, 6.1)
Kyrgyzstan	23.2(20.8, 25.5)	10.6(9.2, 12.1)	11.3(9.7, 12.8)	-0.6(-2.7, 1.5)
Lao PDR	94.8(93.6, 96.1)	95.6(93.8, 97.3)	94.2(92.5, 95.9)	1.3(-1.1, 3.8)
Lebanon	56.3(52.6, 60.1)	58.6(53.6, 63.6)	54.2(48.8, 59.6)	4.4(-3.0, 11.8)
Macedonia	22.5(18.4, 26.5)	24.5(18.8, 30.3)	18.8(13.8, 23.8)	5.7(-1.9, 13.4)
Malawi	39.8(38.3, 41.4)	38.0(35.8, 40.1)	42.1(39.9, 44.3)	-4.1(-7.2, -1.1)
Maldives	76.9(74.1, 79.7)	76.6(72.6, 80.6)	78.0(74.2, 81.9)	-1.5(-7.0, 4.1)
Mali	5.5(4.9, 6.2)	5.7(4.8, 6.6)	5.0(4.1, 5.9)	0.7(-0.6, 2.0)
Mauritania	12.7(11.4, 14.0)	11.9(10.2, 13.5)	12.2(10.5, 13.8)	-0.3(-2.7, 2.1)

Mexico	60.8(56.1, 65.5)	58.6(54.8, 62.4)	62.7(55.1, 70.3)	-4.1(-12.6, 4.4)
Moldova	71.2(67.3, 75.1)	73.6(68.7, 78.5)	67.5(61.7, 73.3)	6.1(-1.6, 13.7)
Mongolia	68.1(66.1, 70.2)	61.2(58.9, 63.6)	63.2(60.8, 65.5)	-1.9(-5.3, 1.4)
Montenegro	40.8(35.8, 45.8)	39.4(32.9, 45.9)	41.8(34.8, 48.9)	-2.5(-12.1, 7.2)
Nepal	51.7(49.0, 54.3)	52.5(48.9, 56.2)	49.9(46.1, 53.7)	2.6(-2.7, 7.9)
Nigeria	36.1(34.9, 37.2)	36.1(34.5, 37.6)	35.8(34.3, 37.4)	0.2(-2.0, 2.4)
Palestine	27.1(25.5, 28.7)	27.7(25.5, 29.9)	26.1(23.8, 28.3)	1.6(-1.5, 4.8)
Panama	37.1(33.4, 40.8)	38.3(33.2, 43.4)	35.0(29.9, 40.2)	3.2(-4.0, 10.5)
Paraguay	31.7(28.8, 34.6)	30.5(26.6, 34.4)	32.5(28.3, 36.6)	-1.9(-7.6, 3.7)
Rwanda	12.8(11.8, 13.9)	12.2(10.7, 13.6)	13.4(11.9, 15.0)	-1.3(-3.4, 0.9)
Senegal	18.4(17.1, 19.6)	18.3(16.6, 20.1)	17.8(16.1, 19.5)	0.5(-1.9, 3.0)
Serbia	51.4(46.0, 56.8)	52.4(44.8, 60.0)	49.4(42.1, 56.7)	3.0(-7.5, 13.6)
Sierra Leone	89.3(86.2, 92.5)	85.6(80.2, 91.0)	92.2(88.8, 95.5)	-6.6(-13.0, -0.2)
South Sudan	5.7(4.8, 6.6)	5.8(4.5, 7.0)	5.6(4.3, 6.8)	0.2(-1.6, 2.0)
Suriname	34.5(31.1, 38.0)	33.4(28.7, 38.1)	35.5(31.0, 40.1)	-2.1(-8.7, 4.4)
Swaziland	29.2(26.1, 32.4)	26.3(22.2, 30.4)	33.7(29.1, 38.4)	-7.5(-13.6, -1.3)
São Tomé and Principe	37.6(33.7, 41.6)	35.4(30.2, 40.7)	39.3(34.0, 44.6)	-3.9(-11.3, 3.6)
Thailand	86.3(84.3, 88.3)	85.5(82.6, 88.3)	86.2(83.2, 89.2)	-0.7(-4.8, 3.4)
Timor-Leste	15.1(12.7, 17.5)	14.8(11.6, 18.0)	13.7(10.8, 16.6)	1.1(-3.3, 5.5)
Togo	28.8(26.2, 31.4)	26.9(23.5, 30.2)	30.7(27.0, 34.5)	-3.9(-8.9, 1.1)
Trinidad and Tobago	85.1(81.6, 88.6)	85.9(81.2, 90.6)	84.2(79.4, 88.9)	1.7(-5.0, 8.4)
Tunisia	44.7(41.1, 48.2)	42.5(37.9, 47.1)	46.8(41.7, 51.8)	-4.3(-11.2, 2.5)
Turkmenistan	44.0(41.3, 46.7)	43.5(39.8, 47.2)	43.7(39.8, 47.7)	-0.2(-5.6, 5.2)
Uganda	42.4(41.2, 43.5)	41.8(40.2, 43.4)	43.5(41.8, 45.1)	-1.7(-4.0, 0.6)
Ukraine	52.9(49.3, 56.4)	54.5(49.8, 59.1)	50.7(45.8, 55.7)	3.7(-3.1, 10.5)
Uruguay	81.9(75.6, 88.2)	59.1(51.6, 66.6)	55.8(44.4, 67.3)	3.2(-10.4, 16.9)
Vietnam	72.5(69.6, 75.4)	74.6(70.7, 78.5)	70.6(66.5, 74.8)	4.0(-1.7, 9.7)
Zimbabwe	21.8(20.4, 23.2)	20.6(18.7, 22.5)	23.9(21.9, 25.9)	-3.3(-6.0, -0.6)

*: Gender gaps are defined as the difference between boy and girl averages. A negative gender gap implies a girl advantage, a positive gender gap a boy advantage.

Table 7. Prevalence (%) of children attending early care and education by place of residence with 95% confidence intervals, the most recent years in 65 countries

Country	Rural	Urban	Difference*
Afghanistan	4.0(2.9, 5.0)	0.6(0.3, 0.8)	-3.4(-4.5, -2.3)
Algeria	22.8(20.9, 24.6)	6.6(5.0, 8.2)	-16.2(-18.6, -13.7)
Argentina	NA	32.1(30.4, 33.7)	NA
Bangladesh	14.2(11.5, 16.8)	13.3(12.4, 14.2)	-0.9(-3.7, 1.9)
Belarus	90.1(87.6, 92.6)	82.5(77.6, 87.5)	-7.5(-13.1, -2.0)
Belize	67.4(62.0, 72.8)	49.1(44.6, 53.7)	-18.3(-25.3, -11.2)
Benin	21.5(19.5, 23.5)	6.8(5.6, 8.1)	-14.6(-17.0, -12.3)
Bhutan	18.4(14.5, 22.4)	6.0(4.6, 7.4)	-12.4(-16.6, -8.2)
Bosnia and Herzegovina	23.1(17.0, 29.2)	7.9(4.3, 11.5)	-15.2(-22.3, -8.1)
Burundi	37.6(34.7, 40.4)	4.1(3.7, 4.4)	-33.5(-36.4, -30.6)
Cameroon	45.3(42.2, 48.4)	15.9(14.0, 17.9)	-29.3(-33.0, -25.7)
Central African Republic	9.7(7.4, 12.0)	2.5(1.7, 3.2)	-7.2(-9.6, -4.8)
Chad	9.2(7.4, 11.0)	1.5(1.0, 2.0)	-7.7(-9.6, -5.9)
Congo, Dem. Rep.	13.4(11.0, 15.8)	3.8(2.5, 5.1)	-9.6(-12.3, -6.9)
Congo, Rep.	50.9(47.5, 54.4)	12.6(11.0, 14.2)	-38.3(-42.2, -34.5)
Costa Rica	21.8(14.5, 29.0)	11.4(6.7, 16.1)	-10.3(-19.0, -1.7)
Cote d'Ivoire	30.1(26.5, 33.7)	5.1(4.0, 6.2)	-25.1(-28.8, -21.3)
Dominican Republic	46.9(44.9, 49.0)	24.4(22.0, 26.8)	-22.5(-25.7, -19.4)
El Salvador	29.3(26.3, 32.3)	20.2(17.6, 22.8)	-9.1(-13.1, -5.1)
Gambia	8.4(7.3, 9.6)	5.6(4.8, 6.4)	-2.8(-4.2, -1.4)
Ghana	80.6(76.9, 84.3)	58.9(55.9, 61.9)	-21.7(-26.5, -17.0)
Guinea	23.0(20.0, 26.0)	2.5(1.8, 3.1)	-20.6(-23.6, -17.5)
Guinea-Bissau	29.4(25.3, 33.4)	3.7(2.9, 4.6)	-25.6(-29.7, -21.5)
Guyana	75.7(70.5, 80.9)	58.9(55.8, 62.1)	-16.8(-22.9, -10.7)
Iraq	88.0(82.1, 93.9)	67.8(32.9, 102.6)	-20.2(-55.7, 15.3)
Jamaica	91.2(87.5, 94.9)	93.8(90.6, 97.0)	2.6(-2.3, 7.5)
Jordan	21.4(19.4, 23.4)	18.6(16.5, 20.8)	-2.7(-5.7, 0.2)
Kazakhstan	63.6(60.1, 67.2)	49.7(44.6, 54.8)	-14.0(-20.2, -7.7)
Kosovo	23.5(17.8, 29.2)	8.8(6.1, 11.6)	-14.7(-21.0, -8.3)
Kyrgyzstan	18.6(16.2, 20.9)	7.7(6.7, 8.8)	-10.8(-13.4, -8.2)
Lao People's Democratic Republic	95.3(93.6, 97.0)	94.4(92.7, 96.2)	-0.8(-3.3, 1.6)
Lebanon	53.0(48.4, 57.7)	63.2(57.3, 69.1)	10.2(2.7, 17.7)
Macedonia	37.2(31.0, 43.4)	6.0(3.3, 8.7)	-31.3(-38.0, -24.5)
Malawi	67.1(62.4, 71.8)	36.5(35.0, 38.1)	-30.6(-35.6, -25.7)
Maldives	81.9(75.3, 88.5)	74.9(72.5, 77.2)	-7.0(-14.1, -0.0)
Mali	15.9(13.7, 18.1)	2.8(2.3, 3.4)	-13.0(-15.3, -10.8)
Mauritania	19.7(17.4, 22.0)	6.4(5.3, 7.4)	-13.3(-15.8, -10.8)
Mexico	60.4(54.5, 66.4)	62.2(57.2, 67.2)	1.8(-6.0, 9.5)
Moldova	82.6(78.9, 86.4)	63.7(58.2, 69.2)	-19.0(-25.6, -12.3)
Mongolia	69.2(67.1, 71.4)	51.8(49.2, 54.4)	-17.4(-20.7, -14.1)
Montenegro	51.9(45.7, 58.1)	19.8(13.8, 25.8)	-32.1(-40.7, -23.4)

Nepal	78.4(73.9, 82.9)	47.1(44.2, 49.9)	-31.3(-36.7, -26.0)
Nigeria	57.0(54.5, 59.4)	26.7(25.6, 27.8)	-30.2(-32.9, -27.5)
Palestine	26.1(24.3, 28.0)	29.3(26.3, 32.4)	3.2(-0.4, 6.8)
Panama	39.4(34.0, 44.8)	32.7(28.9, 36.6)	-6.7(-13.3, -0.0)
Paraguay	40.2(36.1, 44.4)	18.7(15.5, 21.8)	-21.6(-26.8, -16.4)
Rwanda	36.3(32.5, 40.1)	8.4(7.4, 9.4)	-27.9(-31.8, -24.0)
Senegal	31.7(29.0, 34.5)	11.0(9.9, 12.1)	-20.7(-23.7, -17.7)
Serbia	63.2(56.5, 69.8)	28.0(22.5, 33.5)	-35.2(-43.8, -26.6)
Sierra Leone	90.2(86.8, 93.7)	82.2(74.3, 90.1)	-8.0(-16.7, 0.6)
South Sudan	6.2(4.4, 8.0)	5.5(4.5, 6.5)	-0.7(-2.8, 1.4)
Suriname	44.2(39.3, 49.2)	19.7(17.1, 22.4)	-24.5(-30.1, -18.8)
Swaziland	43.4(34.1, 52.6)	26.6(23.6, 29.7)	-16.7(-26.5, -7.0)
São Tomé and Principe	37.8(32.9, 42.7)	36.6(31.1, 42.0)	-1.2(-8.6, 6.1)
Thailand	82.4(78.7, 86.0)	88.2(85.8, 90.6)	5.9(1.5, 10.2)
Timor-Leste	16.9(12.0, 21.9)	13.2(10.8, 15.5)	-3.8(-9.2, 1.7)
Togo	44.8(39.5, 50.2)	21.2(18.7, 23.8)	-23.6(-29.5, -17.7)
Trinidad and Tobago	84.7(79.9, 89.5)	85.4(80.9, 89.9)	0.7(-5.9, 7.3)
Tunisia	60.3(56.0, 64.7)	17.4(13.0, 21.7)	-42.9(-49.1, -36.8)
Turkmenistan	71.3(67.4, 75.2)	29.4(26.2, 32.6)	-41.9(-47.0, -36.9)
Uganda	61.4(58.6, 64.3)	38.4(37.2, 39.6)	-23.1(-26.2, -19.9)
Ukraine	59.4(55.1, 63.7)	36.8(31.9, 41.8)	-22.6(-29.2, -16.0)
Uruguay	59.0(52.1, 65.9)	42.7(28.7, 56.7)	-16.4(-32.0, -0.7)
Vietnam	81.0(76.9, 85.1)	69.1(65.5, 72.8)	-11.9(-17.3, -6.4)
Zimbabwe	27.1(24.1, 30.0)	20.7(19.1, 22.2)	-6.4(-9.8, -3.1)

*: Gaps by residential area are defined as the difference between rural and urban averages. A negative area gap implies urban advantage, a positive area gap rural advantage.

Table 8. Prevalence (%) of children attending early care and education by household wealth quintile with 95% confidence intervals, the most recent years in 65 countries

Country	Wealth quintile (%)					Difference (richest - poorest)*
	Poorest	2	3	4	Richest	
Afghanistan	0.42	0.43	0.75	0.54	4.16	3.7(2.5, 5.0)
Algeria	6.42	11.35	14.83	19.00	29.39	23.0(18.8, 27.1)
Argentina	20.40	24.75	29.38	41.15	48.36	28.0(23.0, 32.9)
Bangladesh	11.35	13.26	12.80	12.76	15.88	4.5(1.7, 7.3)
Belarus	76.16	90.72	89.16	93.98	90.28	14.1(5.6, 22.6)
Belize	27.38	47.11	72.23	64.80	73.54	46.2(36.6, 55.7)
Benin	2.48	5.81	9.04	19.08	42.93	40.5(36.0, 44.9)
Bhutan	2.83	1.56	2.86	9.73	24.50	21.7(16.9, 26.4)
Bosnia and Herzegovina	4.67	2.33	5.14	19.02	32.11	27.4(17.9, 37.0)
Burundi	0.65	2.36	1.76	5.12	29.93	29.3(27.2, 31.3)
Cameroon	2.40	21.54	24.69	41.58	65.37	63.0(58.4, 67.5)
Central African Republic	1.39	1.45	1.88	5.04	11.93	10.5(7.5, 13.5)
Chad	0.35	1.79	1.39	2.35	9.58	9.2(7.4, 11.1)
Congo, Dem. Rep.	1.49	1.05	3.88	4.55	18.22	16.7(13.1, 20.3)
Congo, Rep.	4.05	7.88	13.49	28.67	63.78	59.7(55.4, 64.1)
Costa Rica	8.12	6.84	16.66	11.36	34.78	26.7(13.6, 39.7)
Cote d'Ivoire	1.08	1.86	5.00	10.47	38.16	37.1(32.7, 41.5)
Dominican Republic	13.79	20.49	34.25	47.52	70.25	56.5(52.0, 60.9)
El Salvador	19.79	17.92	18.93	26.03	42.79	23.0(16.1, 29.9)
Gambia	3.52	5.62	5.85	6.18	10.55	7.0(5.2, 8.9)
Ghana	36.50	42.22	54.11	71.34	88.90	52.4(46.0, 58.8)
Guinea	2.12	1.93	2.54	8.28	29.17	27.0(23.0, 31.1)
Guinea-Bissau	2.99	4.04	5.06	4.20	33.26	30.3(25.4, 35.1)
Guyana	44.99	49.31	64.26	72.79	77.77	32.8(24.3, 41.2)
Iraq	57.52	84.36	84.78	94.16	86.45	28.9(-18.5, 76.4)
Jamaica	86.86	88.52	91.02	97.73	98.47	11.6(4.6, 18.6)
Jordan	9.07	11.61	19.00	26.17	32.51	23.4(18.6, 28.3)
Kazakhstan	43.15	54.63	50.99	66.71	70.65	27.5(18.1, 36.9)
Kosovo	5.41	7.86	7.83	13.97	34.60	29.2(19.8, 38.6)
Kyrgyzstan	4.95	5.40	7.32	12.46	22.13	17.2(13.8, 20.6)
Lao PDR	97.51	95.99	93.85	93.61	95.12	-2.4(-5.4, 0.6)
Lebanon	49.07	50.84	59.19	62.25	60.27	11.2(-0.5, 22.9)
Macedonia	0.29	7.38	13.37	36.58	56.66	56.4(45.9, 66.9)
Malawi	27.19	29.90	36.83	41.66	62.56	35.4(30.9, 39.8)
Maldives	67.75	77.12	77.53	74.08	82.33	14.6(6.8, 22.3)
Mali	1.15	0.97	1.52	4.67	19.49	18.3(15.7, 20.9)
Mauritania	3.30	7.19	8.78	14.51	27.77	24.5(20.4, 28.6)
Mexico	57.51	61.33	53.04	62.28	67.01	9.5(-4.8, 23.9)
Moldova	52.63	70.46	73.47	85.32	90.25	37.6(27.9, 47.3)
Mongolia	24.67	59.85	63.56	73.25	85.00	60.3(56.1, 64.6)

Montenegro	6.66	22.04	35.57	63.40	65.41	58.7(47.5, 70.0)
Nepal	38.74	43.50	39.07	45.77	77.67	38.9(31.8, 46.1)
Nigeria	7.43	14.67	29.43	52.62	77.51	70.1(67.5, 72.7)
Palestine	21.66	25.24	21.77	28.92	37.16	15.5(10.5, 20.5)
Panama	25.07	29.55	26.37	24.85	55.40	30.3(21.7, 38.9)
Paraguay	9.21	20.77	27.57	34.32	56.60	47.4(40.2, 54.6)
Rwanda	2.40	5.43	9.18	11.04	43.24	40.8(36.7, 45.0)
São Tomé and Principe	21.84	26.20	24.76	38.53	65.07	43.2(32.5, 53.9)
Senegal	2.53	5.19	12.14	21.46	37.29	34.8(31.4, 38.2)
Serbia	13.28	37.82	48.37	60.75	83.60	70.3(62.4, 78.2)
Sierra Leone	74.01	83.88	79.73	89.23	89.93	15.9(-16.7, 48.5)
South Sudan	1.90	4.08	4.74	8.61	12.06	10.2(7.3, 13.0)
Suriname	12.04	15.43	26.62	36.02	54.65	42.6(35.0, 50.2)
Swaziland	27.75	23.75	26.81	26.32	40.92	13.2(3.3, 23.1)
Thailand	87.13	85.41	88.97	83.77	84.24	-2.9(-8.7, 2.9)
Timor-Leste	8.94	10.73	15.59	14.59	20.76	11.8(4.8, 18.8)
Togo	10.28	19.26	25.87	35.25	45.69	35.4(28.6, 42.2)
Trinidad and Tobago	70.71	83.07	91.83	86.03	91.22	20.5(8.9, 32.1)
Tunisia	7.97	29.25	43.45	46.53	76.84	68.9(61.5, 76.2)
Turkmenistan	19.30	34.27	37.81	68.25	81.05	61.7(54.9, 68.6)
Uganda	19.04	29.35	38.88	52.27	70.58	51.5(48.2, 54.9)
Ukraine	30.30	41.68	52.96	63.30	69.07	38.8(29.3, 48.2)
Uruguay	49.36	56.32	55.95	62.62	70.65	21.3(7.4, 35.1)
Vietnam	53.92	68.59	71.97	80.09	86.59	32.7(24.0, 41.3)
Zimbabwe	16.65	19.07	23.19	21.74	32.77	16.1(11.7, 20.6)

*: Gaps by wealth quintile are defined as the difference between the richest and poorest quintile averages. A negative area gap implies poorest advantage. A positive gap richest advantage.

Table 9. Level and trends of national prevalence (%) of attending early care and education with 95% confidence intervals in 16 countries with data available in two rounds

Country	Round 1	Round 2	Round 1-Round 2
Belize	31.8(28.3, 35.3)	56.0(52.4, 59.6)	-24.2(-29.2, -19.2)
Cameroon	29.0(27.3, 30.8)	28.1(26.3, 29.9)	0.9(-1.6, 3.5)
Chad	4.6(4.0, 5.2)	2.9(2.4, 3.4)	1.7(0.9, 2.5)
Congo, Dem. Rep.	4.9(4.1, 5.6)	6.7(5.5, 7.8)	-1.8(-3.2, -0.4)
Iraq	3.8(3.3, 4.4)	86.9(80.8, 92.9)	-83.0(-89.1, -77.0)
Kazakhstan	37.0(34.6, 39.3)	56.4(53.2, 59.7)	-19.5(-23.5, -15.5)
Lao	23.1(21.7, 24.6)	94.8(93.6, 96.1)	-71.7(-73.2, -69.6)
Mauritania	14.0(12.8, 15.2)	12.0(10.8, 13.2)	2.0(0.3, 3.7)
Mongolia	59.8(56.8, 62.9)	62.2(60.5, 63.9)	-2.4(-5.9, 1.1)
Nigeria	42.8(41.4, 44.2)	36.0(34.9, 37.1)	6.9(5.1, 8.7)
Palestine	17.7(16.5, 18.9)	26.9(25.4, 28.5)	-9.2(-11.2, -7.2)
Serbia	43.8(39.9, 47.8)	51.0(45.7, 56.3)	-7.1(-13.7, -0.5)
Sierra Leone	14.0(12.7, 15.3)	88.9(85.8, 92.1)	-74.9(-78.4, -71.6)
Swaziland	33.1(30.2, 36.0)	30.0(26.9, 33.1)	3.1(-1.1, 7.3)
Thailand	86.1(84.6, 87.7)	85.8(83.7, 87.9)	0.3(-2.3, 2.9)
Vietnam	72.0(69.4, 74.6)	72.6(69.8, 75.5)	-0.6(-4.5, 3.3)

*: A negative sign implies an increase in the % of children attending early care and education, a positive sign a reduction in the % of children attending early care and education

Table 10. Level and trends of gender inequalities in the prevalence (%) of attending early care and education with 95% confidence intervals in 16 countries with data available in two rounds

Country	Round 1			Round 2			Round1 – Round2
	Male	Female	Gap	Male	Female	Gap	
Belize	29.6	33.9	-4.3(-11.3, 2.7)	53.2	59.0	-5.8(-12.9, 1.3)	1.5(-8.4, 11.5)
Cameroon	29.1	28.9	0.2(-3.3, 3.8)	27.0	29.2	-2.3(-5.9, 1.4)	2.3(-2.8, 7.4)
Chad	5.2	4.0	1.2(-0.1, 2.4)	2.9	2.9	-0.0(-1.1, 1.0)	1.2(-0.4, 2.8)
Congo, Dem. Rep.	4.7	5.0	-0.3(-1.9, 1.2)	6.7	6.6	0.1(-2.2, 2.5)	-0.2(-3.4, 2.2)
Iraq	4.1	3.6	0.5(-0.6, 1.6)	84.8	88.9	-4.1(-16.1, 7.9)	3.6(-8.4, 15.6)
Kazakhstan	35.9	38.1	-2.2(-6.9, 2.4)	54.0	58.8	-4.8(-11.2, 1.6)	2.6(-5.4, 10.5)
Lao	21.0	25.3	-4.2(-7.1, -1.3)	95.6	94.2	1.3(-1.1, 3.8)	-2.9(-6.7, 0.9)
Mauritania	13.9	14.1	-0.2(-2.6, 2.3)	11.9	12.2	-0.3(-2.7, 2.1)	0.1(-3.3, 3.5)
Mongolia	58.1	61.5	-3.5(-9.6, 2.7)	61.2	63.2	-1.9(-5.3, 1.4)	-1.5(-8.5, 5.5)
Nigeria	42.5	43.1	-0.6(-3.4, 2.2)	36.1	35.8	0.2(-2.0, 2.4)	-0.4(-4.0, 3.2)
Palestine	18.1	17.3	0.7(-1.7, 3.2)	27.7	26.1	1.6(-1.5, 4.8)	0.9(-3.1, 4.9)
Serbia	40.7	46.6	-6.0(-13.8, 1.9)	52.4	49.4	3.0(-7.5, 13.6)	-3.0(-16.2, 10.1)
Sierra Leone	13.4	14.6	-1.2(-3.8, 1.4)	85.6	92.2	-6.6(-13.0, -0.2)	5.4(-1.4, 12.3)
Swaziland	32.2	33.9	-1.7(-7.5, 4.1)	26.3	33.7	-7.5(-13.6, -1.3)	5.8(-2.7, 14.2)
Thailand	85.2	87.0	-1.8(-4.9, 1.3)	85.5	86.2	-0.7(-4.8, 3.4)	-1.1(-6.2, 4.1)

Vietnam	70.8	73.2	-2.3(-7.6, 2.9)	74.6	70.6	4.0(-1.7, 9.7)	1.7(-6.1, 9.5)

*: Gender gaps are defined as the difference between boy and girl averages. A negative sign implies a reduction of inequality, a positive sign implies an increase of inequality.

Table 11. Level and trends of area inequalities in the prevalence (%) of attending early care and education with 95% confidence intervals in 16 countries with data available in two rounds

Country	Round 1			Round 2			Round1 – Round2
	Rural	Urban	Gap	Rural	Urban	Gap	
Belize	26.4	40.6	-14.1(-21.5, -6.8)	49.1	67.4	-18.3(-25.3, -11.2)	4.2(-6.0, 14.3)
Cameroon	15.1	49.6	-34.5(-38.1, -30.9)	15.9	45.3	-29.3(-33.0, -25.7)	-5.2(-10.3, -0.1)
Chad	2.2	14.7	-12.5(-14.9, -10.1)	1.5	9.2	-7.7(-9.6, -5.9)	-4.8(-7.8, -1.7)
Congo, Dem. Rep.	2.3	12.1	-9.8(-11.9, -7.7)	3.8	13.4	-9.6(-12.3,-6.9)	-0.2(-3.6, 3.2)
Iraq	1.1	5.3	-4.1(-5.1, -3.2)	67.8	88.0	-20.2(-55.5, 15.1)	16.0(-19.3, 51.3)
Kazakhstan	29.4	45.3	-15.9(-20.5, -11.4)	49.7	63.6	-14.0(-20.2, -7.7)	-2.0(-9.7, 5.8)
Lao	15.2	54.9	-39.7(-43.9, -35.6)	94.4	95.3	-0.8(-3.3, 1.6)	-38.9(-43.7, -34.1)
Mauritania	6.2	28.2	-21.9(-24.8, -19.0)	6.4	19.7	-13.3(-15.8, -10.8)	-8.6(-12.5, -4.8)
Mongolia	46.7	69.4	-22.7(-28.6, -16.8)	51.8	69.2	-17.4(-20.7, -14.1)	-5.3(-12.0, 1.4)
Nigeria	32.7	67.5	-34.8(-38.3, -31.3)	26.7	57.0	-30.2(-32.9, -27.5)	-4.6(-9.0, -0.1)
Palestine	18.4	17.4	0.9(-1.7, 3.6)	29.3	26.1	3.2(-0.4, 6.8)	2.3(-2.2, 6.7)
Serbia	28.7	56.6	-27.9(-35.8, -20.0)	28.0	63.2	-35.2(-43.8, -26.6)	7.3(-4.4, 18.9)
Sierra Leone	10.5	23.6	-13.1(-16.4, -9.8)	82.2	90.2	-8.0(-16.7, 0.6)	-5.1(-14.3, 4.1)
Swaziland	32.5	35.6	-3.1(-10.1, 3.9)	26.6	43.4	-16.7(-26.5, -7.0)	13.6(1.6, 25.6)
Thailand	88.7	81.9	6.9(3.7, 10.1)	88.2	82.4	5.9(1.5, 10.2)	-1.0(-6.5, 4.4)
Vietnam	70.6	75.8	-5.2(-10.5, 0.1)	69.1	81.0	-11.9(-17.3, -6.4)	6.7(-1.0, 14.3)

*: Gaps by residential area are defined as the difference between rural and urban averages. A negative sign implies a reduction of inequality, a positive sign implies an increase of inequality.

Table 12. Level and trends of wealth inequalities in the prevalence (%) of attending early care and education with 95% confidence intervals in 16 countries with data available in two rounds

Country	Round 1			Round 2			Round1 – Round2
	Q5	Q1	Gap	Q5	Q1	Gap	
Belize	50.8	12.7	38.1(27.8, 48.4)	73.5	27.4	46.2(36.6, 55.7)	8.1(-6.0, 22.1)
Cameroon	69.0	3.7	65.3(60.7, 69.8)	65.4	2.4	63.0(58.4, 67.5)	-2.3(-8.7, 4.1)
Chad	15.8	1.2	14.7(11.9, 17.4)	9.6	0.3	9.2(7.4, 11.1)	-5.4(-8.8, -2.1)
Congo, Dem. Rep.	16.6	1.6	14.9(11.6, 18.2)	18.2	1.5	16.7(13.1, 20.3)	1.8(-3.1, 6.7)
Iraq	8.9	0.6	8.3(6.6, 10.0)	86.4	57.5	28.9(-17.6, 75.5)	20.6(-26.0, 67.2)
Kazakhstan	60.3	18.6	41.7(35.1, 48.2)	70.6	43.1	27.5(18.1, 36.9)	-14.2(-25.6, -2.7)
Lao	60.8	4.2	56.5(52.7, 60.3)	95.1	97.5	-2.4(-5.4, 0.6)	-54.1(-59.0, -49.3)
Mauritania	38.7	1.4	37.2(33.0, 41.4)	27.8	3.3	24.5(20.4, 28.6)	-12.8(-18.6, -6.9)
Mongolia	79.8	21.0	58.7(51.1, 66.4)	85.0	24.7	60.3(56.1, 64.6)	1.6(-7.1, 10.4)
Nigeria	79.6	9.6	69.9(66.9, 73.0)	77.5	7.4	70.1(67.5, 72.7)	0.1(-3.8, 4.1)
Palestine	27.3	10.2	17.1(13.4, 20.8)	37.2	21.7	15.5(10.5, 20.5)	-1.6(-7.8, 4.6)

Serbia	79.2	24.3	55.0(44.2, 65.8)	83.6	13.3	70.3(62.4, 78.2)	15.4(2.0, 28.7)
Sierra Leone	37.9	5.1	32.9(28.3, 37.4)	89.9	74.0	15.9(-16.7, 48.5)	-16.9(-49.7, 15.9)
Swaziland	45.5	34.8	10.6(1.3, 19.9)	40.9	27.8	13.2(3.3, 23.1)	2.5(-11.0, 16.1)
Thailand	84.3	87.4	-3.1(-8.1, 1.9)	84.2	87.1	-2.9(-8.7, 2.9)	-0.2(-7.8, 7.4)
Vietnam	90.5	62.8	27.7(20.3, 35.1)	86.6	53.9	32.7(24.1, 41.3)	5.0(-6.4, 16.4)

*: Gaps by wealth quintile are defined as the difference between the richest and poorest quintile averages. A negative sign implies a reduction of inequality, a positive sign implies an increase of inequality.

Lu C, *et al. BMJ Global Health* 2020; 5:e002314. doi: 10.1136/bmjgh-2020-002314

(3) Home simulation

Table 13. Percentage of children exposed to high level of home stimulation by gender with 95% confidence intervals, the most recent years in 62 countries

Country	National	Male	Female	Difference*
Afghanistan	73.6(72.3, 74.9)	74.2(72.4, 75.9)	73.4(71.6, 75.2)	0.8(-1.8, 3.3)
Algeria	79.7(78.3, 81.2)	79.3(77.5, 81.2)	78.3(76.4, 80.3)	1.0(-1.7, 3.7)
Argentina	84.0(81.9, 86.1)	73.8(71.7, 76.0)	74.3(72.0, 76.5)	-0.4(-3.5, 2.7)
Bangladesh	79.1(78.0, 80.1)	78.5(77.0, 79.9)	78.5(77.0, 80.1)	-0.1(-2.2, 2.0)
Belarus	96.1(94.5, 97.7)	94.4(91.6, 97.1)	97.4(96.0, 98.9)	-3.1(-6.2, 0.1)
Belize	89.9(87.5, 92.3)	90.8(87.9, 93.6)	88.4(84.7, 92.2)	2.3(-2.4, 7.0)
Benin	28.1(26.6, 29.6)	28.6(26.5, 30.8)	27.5(25.4, 29.6)	1.2(-1.9, 4.2)
Bhutan	54.5(51.9, 57.0)	52.0(48.5, 55.5)	56.6(53.2, 60.1)	-4.6(-9.6, 0.3)
Bosnia and Herzegovina	95.2(93.4, 97.1)	94.9(91.8, 97.9)	95.6(93.8, 97.4)	-0.7(-4.2, 2.9)
Burundi	59.8(59.0, 60.6)	59.2(58.0, 60.3)	60.2(59.0, 61.4)	-1.0(-2.7, 0.6)
Cameroon	45.5(43.3, 47.7)	45.7(42.6, 48.7)	44.7(41.7, 47.7)	1.0(-3.3, 5.2)
Central African Republic	74.1(72.3, 75.9)	74.5(71.9, 77.1)	74.3(71.8, 76.8)	0.2(-3.4, 3.8)
Congo, Rep.	59.8(57.5, 62.2)	60.1(56.9, 63.3)	59.2(55.9, 62.6)	0.8(-3.8, 5.5)
Costa Rica	68.9(63.2, 74.7)	69.2(61.6, 76.7)	66.9(58.6, 75.3)	2.2(-9.1, 13.5)
Cote d'Ivoire	28.9(26.9, 30.8)	29.1(26.4, 31.8)	29.0(26.2, 31.8)	0.1(-3.8, 4.0)
Dominican Republic	60.8(59.1, 62.5)	59.9(57.6, 62.2)	60.6(58.3, 62.9)	-0.7(-4.0, 2.6)
El Salvador	60.6(58.4, 62.9)	57.9(54.6, 61.1)	62.9(59.7, 66.1)	-5.1(-9.6, -0.5)
Gambia	48.3(46.2, 50.4)	49.1(46.2, 52.1)	47.4(44.5, 50.3)	1.7(-2.4, 5.9)
Ghana	40.4(37.7, 43.1)	38.0(34.3, 41.6)	41.9(38.2, 45.7)	-3.9(-9.2, 1.3)
Guinea	31.2(29.4, 33.0)	32.6(30.1, 35.1)	30.1(27.5, 32.7)	2.5(-1.1, 6.1)
Guinea-Bissau	33.0(30.9, 35.1)	41.0(38.0, 44.0)	28.3(25.5, 31.1)	12.7(8.6, 16.8)
Guyana	89.5(87.7, 91.3)	87.7(85.1, 90.4)	90.3(87.9, 92.7)	-2.6(-6.1, 1.0)
Iraq	72.6(64.9, 80.3)	43.7(40.9, 46.4)	45.4(42.8, 47.9)	-1.7(-5.5, 2.1)
Jamaica	88.6(84.7, 92.5)	86.1(80.1, 92.1)	90.1(86.1, 94.2)	-4.0(-11.3, 3.2)
Jordan	80.4(78.8, 82.0)	79.5(77.2, 81.7)	81.0(78.7, 83.3)	-1.6(-4.8, 1.6)
Kazakhstan	87.3(84.4, 90.2)	85.2(80.8, 89.6)	88.1(84.3, 91.9)	-2.9(-8.8, 2.9)
Kosovo	68.2(64.5, 72.0)	67.7(62.6, 72.7)	67.7(62.4, 72.9)	0.0(-7.3, 7.3)
Kyrgyzstan	73.6(71.2, 76.1)	75.0(71.8, 78.2)	72.4(68.9, 75.9)	2.7(-2.1, 7.4)
Lao PDR	63.4(60.7, 66.1)	42.9(40.7, 45.2)	45.7(43.4, 48.0)	-2.8(-6.0, 0.4)
Lebanon	85.0(82.4, 87.7)	85.6(82.1, 89.2)	83.9(79.9, 87.9)	1.7(-3.6, 7.1)
Macedonia	91.8(89.2, 94.5)	92.1(88.5, 95.7)	91.3(87.6, 94.9)	0.8(-4.3, 5.9)
Malawi	30.1(28.6, 31.6)	29.1(27.1, 31.1)	30.7(28.7, 32.8)	-1.7(-4.5, 1.2)
Maldives	97.7(96.7, 98.6)	96.9(95.5, 98.4)	98.2(97.1, 99.4)	-1.3(-3.1, 0.5)
Mali	54.9(53.4, 56.4)	54.3(52.2, 56.4)	55.1(53.0, 57.2)	-0.8(-3.8, 2.2)
Mauritania	45.5(43.6, 47.4)	46.3(43.7, 48.9)	42.4(39.9, 45.0)	3.9(0.3, 7.5)
Mexico	77.0(73.7, 80.2)	72.4(69.1, 75.8)	80.6(75.9, 85.3)	-8.1(-14.0, -2.3)
Moldova	89.4(86.9, 91.9)	86.2(82.4, 89.9)	92.4(89.4, 95.4)	-6.2(-11.0, -1.4)
Mongolia	55.2(53.1, 57.4)	55.2(52.2, 58.1)	54.7(51.8, 57.7)	0.4(-3.8, 4.6)

Montenegro	98.7(97.8, 99.6)	98.6(97.4, 99.7)	98.5(97.0, 99.9)	0.1(-1.7, 2.0)
Nepal	67.9(65.4, 70.4)	70.7(67.4, 74.0)	64.6(61.0, 68.3)	6.1(1.2, 11.0)
Nigeria	63.6(62.4, 64.8)	63.2(61.6, 64.8)	63.8(62.2, 65.5)	-0.7(-3.0, 1.6)
Palestine	79.0(77.5, 80.5)	79.0(76.9, 81.0)	79.1(77.0, 81.2)	-0.1(-3.1, 2.8)
Panama	73.7(70.7, 76.8)	73.2(69.1, 77.3)	73.9(69.4, 78.4)	-0.7(-6.8, 5.4)
Paraguay	64.9(62.0, 67.8)	63.1(59.1, 67.0)	66.1(61.9, 70.2)	-3.0(-8.7, 2.7)
Rwanda	43.6(42.0, 45.3)	42.6(40.4, 44.9)	44.0(41.7, 46.3)	-1.4(-4.6, 1.8)
Senegal	27.9(26.5, 29.2)	26.5(24.7, 28.3)	28.6(26.7, 30.6)	-2.1(-4.8, 0.5)
Serbia	95.5(94.2, 96.7)	94.5(93.1, 95.9)	94.8(93.3, 96.2)	-0.2(-2.3, 1.8)
Sierra Leone	51.9(46.6, 57.2)	28.1(25.9, 30.2)	28.9(26.7, 31.0)	-0.8(-3.8, 2.3)
Suriname	73.6(70.7, 76.5)	72.1(68.1, 76.0)	75.1(71.3, 78.9)	-3.0(-8.5, 2.4)
Swaziland	39.7(36.2, 43.2)	33.5(29.0, 38.0)	45.1(40.3, 49.9)	-11.6(-18.2, -5.0)
São Tomé and Principe	64.9(61.0, 68.8)	64.3(59.1, 69.5)	63.8(58.5, 69.1)	0.5(-6.9, 8.0)
Thailand	93.9(92.8, 95.1)	94.3(92.8, 95.8)	93.4(91.7, 95.1)	0.9(-1.3, 3.2)
Timor-Leste	84.4(81.8, 86.9)	84.8(81.8, 87.8)	79.7(75.8, 83.5)	5.2(0.3, 10.1)
Togo	62.4(59.7, 65.1)	60.6(56.9, 64.4)	62.7(59.0, 66.5)	-2.1(-7.5, 3.2)
Trinidad and Tobago	96.7(94.9, 98.5)	94.9(91.9, 98.0)	96.4(93.9, 99.0)	-1.5(-5.5, 2.4)
Tunisia	71.2(68.0, 74.4)	68.2(63.9, 72.5)	74.3(70.0, 78.7)	-6.1(-12.3, -0.0)
Turkmenistan	96.2(95.1, 97.2)	95.5(94.0, 97.0)	96.8(95.5, 98.2)	-1.3(-3.3, 0.7)
Uganda	51.7(50.6, 52.9)	49.6(48.0, 51.2)	53.5(51.9, 55.1)	-3.9(-6.2, -1.6)
Ukraine	99.2(98.7, 99.6)	98.7(98.0, 99.5)	99.1(98.5, 99.6)	-0.3(-1.3, 0.6)
Uruguay	93.0(90.1, 95.9)	86.4(82.0, 90.8)	77.9(69.7, 86.1)	8.5(-0.8, 17.8)
Vietnam	77.7(75.1, 80.4)	77.3(73.6, 81.0)	77.2(73.4, 80.9)	0.1(-5.1, 5.4)
Zimbabwe	45.0(43.3, 46.7)	44.0(41.6, 46.3)	44.5(42.2, 46.8)	-0.5(-3.8, 2.8)

*: Gender gaps are defined as the difference between boy and girl averages. A negative gender gap implies a girl advantage, a positive gender gap a boy advantage.

Table 14. Percentage of children exposed to high level of home stimulation by place of residence with 95% confidence intervals, the most recent years in 62 countries

Country	Rural	Urban	Difference*
Afghanistan	80.3(78.0, 82.6)	72.6(71.2, 74.1)	-7.7(-10.4, -5.0)
Algeria	84.0(82.6, 85.4)	70.2(67.5, 72.9)	-13.8(-16.8, -10.8)
Argentina	-	74.1(72.5, 75.6)	-
Bangladesh	83.7(81.1, 86.3)	77.2(76.1, 78.3)	-6.5(-9.4, -3.7)
Belarus	97.3(95.9, 98.6)	92.4(88.1, 96.7)	-4.9(-9.4, -0.3)
Belize	93.7(91.0, 96.5)	87.2(83.9, 90.5)	-6.5(-10.8, -2.2)
Benin	34.4(32.1, 36.8)	23.1(21.2, 25.1)	-11.3(-14.4, -8.2)
Bhutan	64.3(59.6, 69.0)	50.2(47.4, 53.1)	-14.1(-19.6, -8.6)
Bosnia and Herzegovina	97.8(96.4, 99.1)	93.9(91.3, 96.4)	-3.9(-6.8, -1.0)
Burundi	78.2(76.0, 80.4)	58.1(57.2, 58.9)	-20.1(-22.5, -17.7)
Cameroon	46.3(43.2, 49.4)	44.3(41.4, 47.2)	-2.0(-6.3, 2.3)
Central African Republic	73.2(70.0, 76.4)	75.1(73.0, 77.2)	1.9(-2.0, 5.7)
Congo, Rep.	64.7(61.3, 68.0)	50.9(48.6, 53.3)	-13.7(-17.8, -9.6)
Costa Rica	68.2(59.2, 77.2)	67.9(62.0, 73.8)	-0.3(-11.0, 10.5)
Cote d'Ivoire	43.0(39.2, 46.8)	20.7(18.8, 22.6)	-22.3(-26.6, -18.0)
Dominican Republic	63.1(61.1, 65.1)	51.6(48.9, 54.3)	-11.5(-14.9, -8.2)
El Salvador	67.1(64.0, 70.2)	51.5(48.3, 54.7)	-15.6(-20.0, -11.1)
Gambia	45.2(41.6, 48.8)	50.5(48.1, 53.0)	5.3(1.0, 9.7)
Ghana	52.8(48.3, 57.3)	30.1(27.2, 33.0)	-22.7(-28.1, -17.3)
Guinea	42.8(39.3, 46.3)	25.8(23.8, 27.8)	-17.0(-21.0, -12.9)
Guinea-Bissau	42.0(37.8, 46.2)	30.2(28.1, 32.3)	-11.8(-16.5, -7.1)
Guyana	91.9(88.6, 95.2)	88.2(86.1, 90.2)	-3.8(-7.7, 0.1)
Iraq	47.4(45.3, 49.5)	38.4(34.5, 42.4)	-8.9(-13.4, -4.5)
Jamaica	88.2(82.5, 93.9)	87.7(83.1, 92.2)	-0.5(-7.8, 6.8)
Jordan	81.1(79.2, 82.9)	76.3(73.9, 78.8)	-4.7(-7.8, -1.6)
Kazakhstan	92.4(90.7, 94.2)	81.2(76.0, 86.4)	-11.2(-16.7, -5.7)
Kosovo	73.0(67.1, 78.8)	64.6(59.9, 69.2)	-8.4(-15.9, -1.0)
Kyrgyzstan	76.1(71.8, 80.4)	72.9(70.0, 75.7)	-3.3(-8.4, 1.9)
Lao People's Democratic Republic	60.1(57.0, 63.1)	38.2(36.4, 40.0)	-21.9(-25.4, -18.3)
Lebanon	81.9(78.3, 85.5)	90.5(87.0, 94.1)	8.6(3.6, 13.7)
Macedonia	94.2(91.3, 97.1)	89.1(84.8, 93.4)	-5.1(-10.3, 0.1)
Malawi	44.0(38.8, 49.2)	28.0(26.6, 29.5)	-16.0(-21.3, -10.6)
Maldives	98.2(96.1, 100.2)	97.2(96.4, 98.1)	-0.9(-3.2, 1.3)
Mali	61.7(58.5, 65.0)	52.9(51.3, 54.6)	-8.8(-12.5, -5.2)
Mauritania	54.0(51.1, 57.0)	37.1(34.9, 39.4)	-16.9(-20.6, -13.2)
Mexico	79.9(76.2, 83.7)	67.8(63.3, 72.4)	-12.1(-18.0, -6.2)
Moldova	90.0(86.9, 93.1)	88.5(85.0, 92.0)	-1.5(-6.1, 3.2)
Mongolia	60.9(58.1, 63.6)	46.7(43.5, 49.8)	-14.2(-18.4, -10.1)
Montenegro	98.7(97.6, 99.8)	98.3(96.7, 99.8)	-0.4(-2.3, 1.5)
Nepal	86.7(82.9, 90.5)	64.8(62.1, 67.6)	-21.9(-26.5, -17.2)
Nigeria	77.1(74.9, 79.4)	57.5(56.2, 58.8)	-19.7(-22.3, -17.1)

Palestine	79.0(77.3, 80.7)	79.2(76.4, 82.0)	0.2(-3.1, 3.5)
Panama	80.8(76.5, 85.1)	62.1(58.4, 65.8)	-18.7(-24.4, -13.0)
Paraguay	73.2(69.4, 77.0)	52.0(48.0, 56.0)	-21.2(-26.7, -15.6)
Rwanda	55.4(51.4, 59.4)	41.0(39.3, 42.8)	-14.4(-18.7, -10.0)
Senegal	35.3(32.6, 38.1)	23.5(22.1, 24.9)	-11.9(-14.9, -8.8)
Serbia	96.1(95.2, 97.1)	92.1(90.0, 94.2)	-4.0(-6.4, -1.7)
Sierra Leone	40.2(37.1, 43.3)	21.4(19.9, 22.9)	-18.8(-22.2, -15.4)
Suriname	81.8(77.9, 85.7)	61.3(58.1, 64.5)	-20.5(-25.5, -15.4)
Swaziland	53.6(44.3, 62.9)	35.7(32.4, 39.0)	-17.9(-27.8, -8.1)
São Tomé and Principe	65.1(60.3, 69.9)	62.0(56.4, 67.7)	-3.1(-10.5, 4.3)
Thailand	96.9(95.9, 97.8)	91.7(90.0, 93.5)	-5.1(-7.1, -3.1)
Timor-Leste	89.0(84.9, 93.0)	79.5(76.5, 82.5)	-9.4(-14.5, -4.4)
Togo	61.2(55.8, 66.5)	61.9(58.9, 64.9)	0.7(-5.5, 6.8)
Trinidad and Tobago	94.7(91.7, 97.8)	97.0(94.9, 99.1)	2.2(-1.5, 5.9)
Tunisia	82.1(78.7, 85.5)	52.1(46.8, 57.4)	-30.0(-36.3, -23.6)
Turkmenistan	98.3(97.2, 99.4)	95.0(93.6, 96.5)	-3.2(-5.0, -1.4)
Uganda	68.0(65.3, 70.7)	47.8(46.6, 49.1)	-20.2(-23.1, -17.2)
Ukraine	99.0(98.4, 99.5)	98.7(97.9, 99.5)	-0.2(-1.2, 0.7)
Uruguay	83.3(78.7, 87.8)	75.8(64.6, 87.0)	-7.5(-19.6, 4.6)
Vietnam	86.0(82.6, 89.4)	73.6(70.1, 77.0)	-12.4(-17.3, -7.6)
Zimbabwe	56.4(53.1, 59.7)	40.3(38.4, 42.1)	-16.2(-20.0, -12.3)

*: Gaps by residential area are defined as the difference between rural and urban averages. A negative area gap implies urban advantage, a positive area gap rural advantage.

Table 15. Percentage of children exposed to high level of home stimulation by household wealth quintile with 95% confidence intervals, the most recent years in 62 countries

Country	Wealth quintile (%)					Difference (richest - poorest)*
	Poorest	2	3	4	Richest	
Afghanistan	72.57	73.29	69.95	74.50	81.40	8.8(5.2, 12.5)
Algeria	63.98	73.86	77.47	83.95	91.73	27.7(23.6, 31.9)
Argentina	63.36	69.80	75.56	80.23	84.55	21.2(16.5, 25.9)
Bangladesh	63.31	69.85	80.69	85.47	91.20	27.9(24.8, 31.0)
Belarus	91.72	97.01	96.01	95.46	99.10	7.4(2.2, 12.5)
Belize	75.74	85.25	86.27	95.02	96.03	16.0(9.9, 22.2)
Benin	17.76	22.23	23.65	36.61	50.98	33.2(28.1, 38.3)
Bhutan	39.98	48.03	46.87	58.71	71.01	31.0(24.1, 38.0)
Bosnia and Herzegovina	87.63	95.95	94.07	98.26	99.56	11.9(7.3, 16.6)
Burundi	58.11	59.02	57.57	56.01	70.47	12.4(9.7, 15.1)
Cameroon	51.57	41.10	35.47	42.03	52.30	0.7(-5.8, 7.2)
Central African Republic	70.28	74.66	76.72	74.47	75.43	5.1(-0.4, 10.7)
Congo, Rep.	43.41	48.87	54.08	57.65	68.97	25.6(19.7, 31.4)
Costa Rica	56.94	55.48	61.29	72.46	84.06	27.1(14.1, 40.2)
Cote d'Ivoire	16.66	19.51	21.37	25.29	49.16	32.5(27.0, 38.0)
Dominican Republic	35.26	48.94	62.00	65.75	74.61	39.3(34.3, 44.4)
El Salvador	46.12	51.53	59.45	67.81	77.06	30.9(24.1, 37.8)
Gambia	49.35	51.95	45.56	44.13	51.13	1.8(-4.4, 8.0)
Ghana	19.66	24.08	29.92	31.20	61.50	41.8(35.3, 48.4)
Guinea	21.40	23.26	28.41	35.13	47.57	26.2(20.7, 31.7)
Guinea-Bissau	32.47	31.77	27.65	28.58	44.71	12.2(5.9, 18.5)
Guyana	80.68	83.68	88.59	93.70	96.35	15.7(9.9, 21.4)
Iraq	30.02	40.46	46.57	57.11	62.64	23.5(18.3, 28.8)
Jamaica	72.30	90.35	95.78	93.67	86.98	14.7(-2.5, 31.9)
Jordan	68.81	83.40	77.23	84.35	85.12	16.3(11.2, 21.4)
Kazakhstan	81.66	84.34	90.66	94.60	96.27	16.9(8.4, 25.4)
Kosovo	54.38	65.02	70.01	70.18	80.30	25.9(15.1, 36.8)
Kyrgyzstan	62.01	72.70	77.98	76.70	79.03	17.0(9.4, 24.6)
Lao PDR	30.3	32.7	37.2	44.9	68.1	37.8(33.2, 42.4)
Lebanon	78.99	82.42	85.18	86.38	90.19	11.2(2.7, 19.7)
Macedonia	68.03	61.01	59.02	62.35	75.82	13.1(4.5, 21.6)
Malawi	22.43	25.56	29.91	29.41	40.94	18.5(14.0, 23.0)
Maldives	97.34	97.24	97.60	92.84	99.66	2.3(0.5, 4.1)
Mali	53.74	50.69	49.14	55.20	65.46	11.7(7.1, 16.3)
Mauritania	41.88	40.87	47.61	52.94	63.22	32.8(27.3, 38.3)
Mexico	60.07	72.06	75.78	77.89	90.00	29.9(22.7, 37.2)
Moldova	82.88	86.61	93.26	95.68	94.11	11.2(4.0, 18.5)
Mongolia	41.76	44.59	57.26	61.50	72.37	31.8(25.6, 38.0)
Montenegro	94.09	98.69	100.0	99.41	100.0	5.9(1.8, 10.1)

Lu C, *et al. BMJ Global Health* 2020; 5:e002314. doi: 10.1136/bmjgh-2020-002314

Nepal	45.66	57.65	63.15	69.92	86.14	40.5(33.8, 47.2)
Nigeria	46.69	51.89	59.79	71.04	87.54	42.3(39.3, 45.3)
Palestine	63.57	66.71	72.31	77.48	81.85	16.9(12.4, 21.3)
Panama	37.42	56.93	67.26	79.09	86.43	49.0(41.4, 56.6)
Paraguay	39.77	46.07	67.36	74.39	87.70	47.9(40.4, 55.5)
Rwanda	28.44	38.28	45.36	47.00	61.38	32.9(27.8, 38.0)
São Tomé and Principe	17.71	22.94	26.65	26.97	37.26	19.6(15.7, 23.4)
Senegal	87.04	95.76	98.07	96.71	97.48	10.3(6.5, 14.0)
Serbia	32.65	30.54	36.75	39.45	56.11	27.0(22.4, 31.6)
Sierra Leone	18.4	17.8	22.2	28.1	45.5	23.5(19.6, 27.5)
Suriname	30.62	44.23	41.26	48.31	59.30	30.7(20.9, 40.5)
Swaziland	48.91	61.46	60.78	70.46	73.27	24.4(12.8, 35.9)
Thailand	88.25	92.63	94.26	97.18	98.95	11.2(7.7, 14.7)
Timor-Leste	73.45	81.46	79.85	91.30	86.83	13.4(5.6, 21.1)
Togo	52.03	63.09	64.69	59.06	66.93	14.9(6.8, 23.0)
Trinidad and Tobago	95.20	93.22	92.91	97.89	98.58	3.4(-2.1, 8.9)
Tunisia	43.98	57.86	66.06	83.24	90.48	46.5(37.8, 55.2)
Turkmenistan	93.77	95.54	96.60	97.91	98.76	5.0(2.1, 7.9)
Uganda	35.50	44.44	48.76	55.33	71.71	36.2(32.7, 39.7)
Ukraine	98.50	99.60	98.09	98.82	99.47	1.0(-0.4, 2.3)
Uruguay	72.21	82.30	82.90	89.55	91.77	19.6(8.9, 30.2)
Vietnam	54.47	73.71	78.29	81.93	95.08	48.4(40.6, 56.2)
Zimbabwe	36.01	39.24	42.50	48.02	59.71	23.7(18.6, 28.8)

*: Gaps by wealth quintile are defined as the difference between the richest and poorest quintile averages. A negative area gap implies poorest advantage. A positive gap richest advantage.

Table 16. Level and trends of national prevalence (%) of home stimulation with 95% confidence intervals in 13 countries with data available in two rounds

Country	Round 1	Round 2	Round 1-Round 2
Belize	85.7(83.1, 88.2)	89.6(87.3, 92.0)	-3.9(-7.4, -0.5)
Iraq	53.5(52.2, 54.7)	44.5(42.6, 46.4)	9.0(6.7, 11.3)
Kazakhstan	91.5(90.0, 93.0)	86.7(83.7, 89.6)	4.8(1.6, 8.1)
Lao	57.5(55.8, 59.1)	44.3(42.6, 45.9)	13.2(10.9, 15.5)
Mauritania	54.5(52.8, 56.3)	44.4(42.5, 46.2)	10.2(7.6, 12.7)
Mongolia	58.9(55.9, 62.0)	55.0(52.9, 57.1)	4.0(0.2, 7.7)
Nigeria	65.8(64.5, 67.1)	63.5(62.3, 64.6)	2.3(0.5, 4.0)
Palestine	66.6(65.1, 68.2)	79.0(77.6, 80.5)	-12.4(-14.5, -10.3)
Serbia	95.2(93.6, 96.8)	94.6(93.6, 95.7)	0.5(-1.4, 2.4)
Sierra Leone	54.4(52.5, 56.3)	28.5(26.9, 29.9)	25.9(23.5, 28.3)
Swaziland	50.1(47.0, 53.2)	39.3(36.0, 42.6)	10.8(6.2, 15.4)
Thailand	94.3(93.2, 95.4)	93.9(92.7, 95.0)	0.4(-1.1, 2.0)
Vietnam	76.8(74.2, 79.4)	77.2(74.6, 79.8)	-0.4(-4.1, 3.2)

*: A negative sign implies an increase in the % of children exposed to high stimulation, a positive sign a reduction in the % of children exposed to high stimulation.

Table 17. Level and trends of gender inequalities in the prevalence (%) of home stimulation with 95% confidence intervals in 13 countries with data available in two rounds

Country	Round 1			Round 2			Round1 – Round2
	Male	Female	Gap	Male	Female	Gap	
Belize	88.4	83	5.3(0.2, 10.4)	90.8	88.4	2.3(-2.4, 7.0)	-3.0(-9.9, 3.9)
Iraq	52.8	54.1	-1.3(-3.8, 1.2)	43.7	45.4	-1.7(-5.5, 2.1)	0.4(-4.1, 4.9)
Kazakhstan	91.9	91.1	0.8(-2.3, 3.8)	85.2	88.1	-2.9(-8.7, 2.9)	2.1(-4.5, 8.1)
Lao	57.4	57.5	-0.1(-3.4, 3.2)	42.9	45.7	-2.8(-6.0, 0.4)	2.7(-1.9, 7.3)
Mauritania	53.7	55.4	-1.8(-5.2, 1.7)	46.3	42.4	3.9(0.3, 7.5)	2.1(-2.9, 7.2)
Mongolia	55.7	62	-6.3(-12.3, -0.2)	55.2	54.7	0.4(-3.8, 4.6)	-5.9(-13.3, 1.5)
Nigeria	66.7	64.8	2.0(-0.6, 4.6)	63.2	63.8	-0.7(-3.0, 1.6)	1.3(-0.8, 3.1)
Palestine	67	66.2	0.8(-2.3, 3.9)	79	79.1	-0.1(-3.1, 2.8)	-0.7(-4.9, 3.6)
Serbia	95.5	94.9	0.6(-2.6, 3.8)	94.5	94.8	-0.2(-2.3, 1.8)	-0.4(-4.5, 3.1)
Sierra Leone	53.3	55.5	-2.2(-5.9, 58.1)	28.1	28.8	-0.8(-3.8, 2.3)	-1.5(-6.3, 3.3)
Swaziland	50.5	49.7	0.8(-5.4, 7.1)	33.5	45.1	-11.6(-18.2, -5.0)	10.8(1.7, 19.9)
Thailand	93.9	94.7	-0.8(-3.0, 1.4)	94.3	93.4	0.9(-1.3, 3.2)	0.1(-3.0, 2.3)
Vietnam	74.1	79.5	-5.4(-10.6, -0.3)	77.3	77.2	0.1(-5.1, 5.4)	-5.3(-12.6, 2.1)

*: Gender gaps are defined as the difference between boy and girl averages. A negative sign implies a reduction of inequality, a positive sign implies an increase of inequality.

Table 18. Level and trends of area inequalities in the prevalence (%) of home stimulation with 95% confidence intervals in 13 countries with data available in two rounds

Country	Round 1			Round 2			Round1 – Round2
	Rural	Urban	Gap	Rural	Urban	Gap	
Belize	84	88.5	-4.6(-9.7, 0.6)	87.2	93.7	-6.5(-10.8, -2.2)	2.0(-4.8, 8.7)
Iraq	41.2	59.9	-18.7(-21.1, -16.3)	38.4	47.4	-8.9(-13.4, -4.5)	-9.8(-14.8, -4.7)
Kazakhstan	89.3	94	-4.7(-7.7, -1.8)	81.2	92.4	-11.2(-16.7, -5.7)	6.5(-0.2, 12.7)
Lao	52.4	77.6	-25.2(-29.0, -21.4)	38.2	60.1	-21.9(-25.4, -18.3)	-3.3(-8.5, 1.9)
Mauritania	53.6	56.1	-2.5(-6.2, 1.2)	37.1	54	-16.9(-20.6, -13.2)	14.4(9.2, 19.7)
Mongolia	49.7	65.5	-15.8(-21.6, -9.9)	46.7	60.9	-14.2(-18.4, -10.1)	-1.5(-8.7, 5.6)
Nigeria	60	79.6	-19.6(-22.6, -16.5)	57.5	77.1	-19.7(-22.3, -17.1)	0.1(-3.9, 4.1)
Palestine	68.7	65.9	2.8(-0.6, 6.2)	79.2	79	0.2(-3.1, 3.5)	-2.6(-7.3, 2.1)
Serbia	94.4	95.8	-1.5(-4.8, 1.8)	92.1	96.1	-4.0(-6.4, -1.7)	2.5(-1.5, 6.6)
Sierra Leone	62.3	51.5	10.7(6.5, 14.9)	40.2	21.4	18.8(15.3, 22.2)	8.0(2.6, 13.4)
Swaziland	48.1	59.1	-10.9(-18.2, -3.6)	35.7	53.6	-17.9(-27.8, -8.1)	7.0(-5.3, 19.2)
Thailand	94.7	93.7	1.0(-1.1, 3.2)	91.7	96.9	-5.1(-7.1, -3.1)	4.1(1.2, 7.1)
Vietnam	73.7	85.3	-11.5(-16.2, -6.9)	73.6	86	-12.4(-17.3, -7.6)	0.9(-5.8, 7.6)

*: Gaps by residential area are defined as the difference between rural and urban averages. A negative sign implies a reduction of inequality, a positive sign implies an increase of inequality.

Table 19. Level and trends of wealth inequalities in the prevalence (%) of home stimulation with 95% confidence intervals in 13 countries with data available in two rounds

Country	Round 1			Round 2			Round1 – Round2
	Q5	Q1	Gap	Q5	Q1	Gap	
Belize	95.2	67.9	27.3(18.8, 35.9)	96.7	80.7	16.0(9.9, 22.2)	-11.3(-21.8, -0.8)
Iraq	69.6	31.2	38.4(35.0, 41.8)	52.2	28.6	23.5(18.3, 28.8)	-14.9(-21.1, -8.6)
Kazakhstan	96.3	84.4	12.0(7.3, 16.6)	96.2	79.3	16.9(8.4, 25.4)	4.9(-4.7, 14.6)
Lao	84.1	40.8	43.2(38.9, 47.7)	75.6	35.3	37.8(33.2, 42.4)	-5.5(-11.8, 0.8)
Mauritania	63.3	56.9	6.4(1.0, 11.8)	63.1	30.3	32.8(27.4, 38.3)	26.4(18.7, 34.1)
Mongolia	73.4	46.0	27.4(18.7, 36.1)	71.6	39.8	31.8(25.6, 38.0)	4.4(-6.3, 15.1)
Nigeria	87.0	47.9	39.1(35.7, 42.5)	88.1	45.8	42.3(39.3, 45.3)	3.2(-1.3, 7.7)
Palestine	77.4	56.8	20.5(15.8, 25.2)	87.7	70.8	16.9(12.4, 21.3)	-3.7(-10.1, 2.8)
Serbia	97.2	86.5	10.7(3.6, 17.7)	97.7	87.4	10.3(6.5, 14.0)	-0.4(-8.4, 7.6)
Sierra Leone	77.6	46.2	31.45(26.2, 36.7)	45.5	18.5	23.5(19.6, 27.5)	-8.2(-11.4, 2.5)
Swaziland	67.8	36.1	31.7(22.7, 40.8)	53.6	22.9	30.7(21.0, 40.4)	-1.0(-14.4, 12.3)
Thailand	98.8	88.6	10.2(6.7, 13.7)	99.0	87.8	11.2(7.7, 14.7)	1.0(-4.0, 5.9)
Vietnam	94.4	60.1	34.3(27.0, 41.7)	95.7	47.3	48.4(40.6, 56.1)	14.0(3.4, 24.7)

*: Gaps by wealth quintile are defined as the difference between the richest and poorest quintile averages. A negative sign implies a reduction of inequality, a positive sign implies an increase of inequality.

(4) Early Childhood Development Index

Table 20. Prevalence (%) of children on track according to the ECDI by gender with 95% confidence intervals, the most recent years in 60 countries

Country	National	Male	Female	Difference*
Algeria	74.1(72.5, 75.7)	71.3(69.0, 73.6)	77.3(75.2, 79.4)	-6.0(-9.1, -2.9)
Argentina	87.0(85.0, 89.0)	85.0(82.0, 88.0)	89.2(86.6, 91.8)	-4.1(-8.1, -0.2)
Bangladesh	65.6(64.2, 66.9)	63.4(61.6, 65.2)	67.7(65.8, 69.5)	-4.2(-6.9, -1.6)
Belarus	94.2(92.7, 95.7)	92.2(89.4, 94.9)	96.2(95.0, 97.4)	-4.0(-7.0, -1.0)
Belize	84.8(82.2, 87.4)	81.7(77.8, 85.6)	88.2(84.8, 91.5)	-6.5(-11.6, -1.4)
Benin	62.8(61.2, 64.5)	58.7(56.3, 61.1)	66.7(64.5, 69.0)	-8.0(-11.3, -4.7)
Bhutan	72.3(70.0, 74.6)	68.8(65.5, 72.2)	76.1(73.0, 79.2)	-7.3(-11.8, -2.7)
Bosnia and Herzegovina	96.9(95.3, 98.5)	96.1(93.1, 99.2)	97.7(96.4, 98.9)	-1.5(-4.8, 1.7)
Burundi	40.3(39.5, 41.1)	36.9(35.7, 38.0)	43.8(42.6, 45.0)	-6.9(-8.6, -5.3)
Cameroon	62.5(60.4, 64.6)	60.2(57.2, 63.2)	64.6(61.7, 67.5)	-4.4(-8.6, -0.2)
Central African Republic	48.7(46.6, 50.7)	45.1(42.2, 48.0)	51.7(48.8, 54.5)	-6.5(-10.6, -2.5)
Chad	36.3(34.9, 37.8)	34.4(32.3, 36.5)	38.4(36.3, 40.5)	-4.0(-7.0, -1.1)
Congo, Dem. Rep.	67.9(66.1, 69.6)	66.9(64.3, 69.5)	68.7(66.3, 71.2)	-1.8(-5.4, 1.7)
Congo, Rep.	62.3(60.0, 64.6)	58.4(55.2, 61.7)	66.2(63.1, 69.3)	-7.8(-12.3, -3.3)
Costa Rica	83.5(79.9, 87.1)	82.7(77.5, 87.8)	84.2(79.2, 89.1)	-1.5(-8.7, 5.7)
Cote d'Ivoire	64.3(62.3, 66.3)	62.5(59.7, 65.3)	66.5(63.6, 69.3)	-4.0(-8.0, 0.0)
Dominican Republic	87.8(86.7, 89.0)	86.9(85.4, 88.5)	88.8(87.1, 90.4)	-1.8(-4.1, 0.4)
El Salvador	82.8(81.1, 84.5)	80.8(78.2, 83.3)	84.9(82.7, 87.2)	-4.2(-7.6, -0.8)
Gambia	69.2(67.3, 71.1)	66.6(63.8, 69.3)	71.3(68.8, 73.9)	-4.8(-8.5, -1.0)
Ghana	74.4(72.0, 76.7)	70.5(67.1, 74.0)	78.2(75.1, 81.3)	-7.7(-12.3, -3.0)
Guinea	49.7(47.7, 51.6)	48.1(45.4, 50.7)	50.6(47.8, 53.4)	-2.6(-6.4, 1.3)
Guinea-Bissau	63.0(60.8, 65.2)	58.2(55.1, 61.3)	67.6(64.5, 70.7)	-9.4(-13.8, -5.0)
Guyana	87.4(85.5, 89.3)	86.9(84.2, 89.6)	87.8(85.1, 90.5)	-0.8(-4.7, 3.0)
Jamaica	90.3(87.7, 93.0)	87.0(82.7, 91.3)	94.6(92.0, 97.2)	-7.6(-12.6, -2.6)
Jordan	67.3(65.3, 69.3)	64.2(61.4, 67.0)	70.8(68.1, 73.5)	-6.6(-10.5, -2.7)
Kazakhstan	87.2(84.9, 89.6)	85.8(82.4, 89.2)	88.7(85.5, 91.8)	-2.9(-7.6, 1.8)
Kosovo	85.9(83.1, 88.7)	81.7(77.4, 86.1)	90.3(87.0, 93.7)	-8.6(-14.1, -3.1)
Kyrgyzstan	81.8(79.6, 84.0)	78.6(75.4, 81.8)	85.1(82.2, 88.1)	-6.5(-10.9, -2.2)
Lebanon	86.6(84.0, 89.1)	86.2(82.6, 89.7)	86.9(83.3, 90.6)	-0.7(-5.8, 4.3)
Macedonia	93.7(91.5, 95.9)	93.9(91.2, 96.7)	93.4(90.0, 96.8)	0.6(-3.8, 5.0)
Malawi	61.7(60.1, 63.2)	57.7(55.5, 59.9)	65.9(63.8, 68.1)	-8.2(-11.3, -5.1)
Maldives	92.9(91.3, 94.6)	91.9(89.4, 94.4)	94.0(92.0, 96.1)	-2.1(-5.4, 1.1)
Mali	62.8(61.3, 64.2)	61.3(59.4, 63.3)	65.4(63.5, 67.4)	-4.1(-6.9, -1.4)
Mauritania	62.1(60.2, 64.0)	61.0(58.5, 63.6)	64.3(61.8, 66.8)	-3.3(-6.9, 0.3)
Mexico	83.3(80.6, 86.0)	79.6(76.9, 82.3)	86.5(82.5, 90.4)	-6.8(-11.7, -2.0)
Moldova	84.5(81.4, 87.5)	84.2(80.0, 88.4)	84.8(80.4, 89.3)	-0.6(-6.8, 5.5)
Mongolia	77.5(75.6, 79.3)	74.7(71.9, 77.4)	80.3(77.9, 82.8)	-5.7(-9.3, -2.0)
Montenegro	96.0(93.9, 98.1)	95.4(92.0, 98.7)	96.1(93.5, 98.7)	-0.8(-5.0, 3.5)
Nepal	65.6(63.1, 68.2)	63.5(59.9, 67.1)	68.2(64.6, 71.9)	-4.8(-9.9, 0.4)

Nigeria	62.7(61.6, 63.9)	61.6(60.0, 63.2)	63.8(62.2, 65.5)	-2.3(-4.6, 0.1)
Palestine	73.9(72.3, 75.5)	69.4(67.1, 71.7)	79.0(76.8, 81.1)	-9.6(-12.7, -6.4)
Panama	80.2(77.0, 83.4)	79.6(75.0, 84.2)	81.0(76.7, 85.2)	-1.4(-7.6, 4.9)
Paraguay	83.0(80.8, 85.2)	81.3(78.2, 84.5)	84.8(81.8, 87.8)	-3.5(-7.8, 0.9)
Rwanda	72.1(70.6, 73.6)	70.8(68.7, 72.9)	73.6(71.6, 75.7)	-2.9(-5.8, 0.1)
Senegal	66.0(64.6, 67.3)	64.1(62.1, 66.0)	67.9(66.0, 69.8)	-3.8(-6.6, -1.1)
Serbia	96.6(95.5, 97.8)	96.5(94.7, 98.2)	96.9(95.4, 98.4)	-0.4(-2.7, 1.9)
Suriname	74.0(71.1, 76.9)	68.5(64.0, 72.9)	78.1(74.4, 81.9)	-9.7(-15.5, -3.8)
Swaziland	66.7(63.5, 69.9)	64.8(60.2, 69.4)	68.6(64.1, 73.1)	-3.8(-10.2, 2.6)
São Tomé and Principe	58.2(54.1, 62.2)	57.2(51.6, 62.8)	59.0(53.2, 64.8)	-1.8(-9.9, 6.2)
Thailand	92.6(91.1, 94.1)	91.4(89.3, 93.6)	93.8(91.8, 95.8)	-2.3(-5.3, 0.6)
Timor-Leste	56.1(52.8, 59.4)	54.6(49.9, 59.3)	57.8(53.2, 62.4)	-3.2(-9.8, 3.3)
Togo	55.8(53.0, 58.6)	53.7(49.8, 57.5)	58.4(54.5, 62.3)	-4.8(-10.3, 0.7)
Trinidad and Tobago	92.4(89.7, 95.1)	91.3(87.2, 95.3)	93.5(89.9, 97.1)	-2.2(-7.7, 3.2)
Tunisia	77.6(74.7, 80.6)	73.9(69.6, 78.2)	81.9(78.0, 85.9)	-8.1(-13.9, -2.2)
Turkmenistan	92.8(91.3, 94.3)	92.6(90.6, 94.6)	93.0(90.9, 95.1)	-0.4(-3.4, 2.5)
Uganda	66.2(65.2, 67.3)	66.0(64.4, 67.5)	66.5(65.0, 68.1)	-0.6(-2.7, 1.6)
Ukraine	90.9(89.1, 92.7)	91.5(89.3, 93.7)	90.5(87.7, 93.3)	1.0(-2.5, 4.6)
Uruguay	86.9(80.7, 93.0)	88.0(80.5, 95.5)	85.7(75.8, 95.5)	2.3(-10.1, 14.7)
Vietnam	90.6(88.7, 92.5)	89.9(87.0, 92.7)	91.3(88.8, 93.8)	-1.4(-5.2, 2.4)
Zimbabwe	63.8(62.2, 65.5)	60.8(58.4, 63.2)	67.0(64.7, 69.2)	-6.2(-9.5, -2.9)

*: Gender gaps are defined as the difference between boy and girl averages. A negative gender gap implies a girl advantage, a positive gender gap a boy advantage.

Table 21. Percentage (%) of children on track according to the ECDI by place of residence with 95% confidence intervals, the most recent years in 60 countries

Country	Rural	Urban	Difference*
Algeria	74.5(72.6, 76.5)	73.7(71.0, 76.4)	-0.8(-4.1, 2.5)
Bangladesh	72.2(68.9, 75.5)	63.7(62.3, 65.1)	-8.4(-12.0, -4.9)
Belarus	95.4(94.0, 96.7)	91.1(87.1, 95.1)	-4.3(-8.6, -0.0)
Belize	88.5(84.9, 92.0)	82.6(79.1, 86.1)	-5.9(-10.9, -0.8)
Benin	65.9(63.5, 68.3)	60.3(58.1, 62.6)	-5.6(-8.9, -2.3)
Bhutan	77.7(73.5, 81.9)	70.1(67.4, 72.9)	-7.6(-12.6, -2.6)
Bosnia and Herzegovina	98.4(97.2, 99.7)	96.1(93.7, 98.5)	-2.3(-5.0, 0.4)
Burundi	56.0(53.2, 58.9)	38.9(38.1, 39.8)	-17.1(-20.1, -14.1)
Cameroon	70.2(67.3, 73.0)	57.1(54.2, 60.0)	-13.1(-17.2, -9.0)
Central African Republic	49.2(45.6, 52.8)	48.2(45.7, 50.6)	-1.0(-5.4, 3.3)
Chad	46.0(42.3, 49.6)	34.4(32.8, 36.0)	-11.6(-15.6, -7.6)
Congo, Dem. Rep.	73.0(70.1, 75.8)	65.6(63.4, 67.8)	-7.4(-11.0, -3.8)
Congo, Rep.	69.4(66.2, 72.7)	49.5(47.1, 51.9)	-19.9(-23.9, -15.9)
Costa Rica	86.5(82.0, 91.0)	79.9(74.5, 85.3)	-6.6(-13.6, 0.5)
Cote d'Ivoire	67.8(64.2, 71.4)	62.4(60.1, 64.8)	-5.4(-9.7, -1.1)
Dominican Republic	88.8(87.5, 90.1)	84.9(82.8, 87.1)	-3.9(-6.4, -1.3)
El Salvador	83.6(81.2, 86.0)	81.8(79.5, 84.2)	-1.8(-5.1, 1.6)
Gambia	69.9(66.6, 73.3)	68.2(66.0, 70.4)	-1.7(-5.7, 2.3)
Ghana	78.2(74.4, 82.1)	71.3(68.5, 74.2)	-6.9(-11.7, -2.1)
Guinea	54.0(50.4, 57.6)	47.0(44.8, 49.3)	-7.0(-11.2, -2.7)
Guinea-Bissau	61.2(56.7, 65.8)	63.9(61.6, 66.2)	2.7(-2.4, 7.7)
Guyana	90.8(87.2, 94.4)	86.4(84.2, 88.6)	-4.4(-8.6, -0.2)
Jamaica	91.2(87.6, 94.7)	89.4(85.4, 93.3)	-1.8(-7.1, 3.5)
Jordan	67.4(65.1, 69.7)	67.1(64.5, 69.8)	-0.3(-3.8, 3.3)
Kazakhstan	87.6(85.3, 89.9)	86.9(82.9, 90.9)	-0.7(-5.3, 3.9)
Kosovo	84.8(80.0, 89.7)	86.4(83.0, 89.8)	1.6(-4.4, 7.5)
Kyrgyzstan	81.2(77.4, 84.9)	82.0(79.4, 84.7)	0.8(-3.8, 5.4)
Lebanon	86.7(83.5, 89.9)	86.2(82.0, 90.4)	-0.5(-5.8, 4.8)
Macedonia	91.7(88.5, 95.0)	95.8(92.9, 98.6)	4.1(-0.3, 8.4)
Malawi	71.3(66.3, 76.4)	60.5(58.9, 62.1)	-10.8(-16.1, -5.5)
Maldives	95.6(91.8, 99.4)	91.6(90.1, 93.1)	-4.0(-8.1, 0.1)
Mali	67.4(64.5, 70.3)	62.3(60.7, 63.8)	-5.2(-8.4, -1.9)
Mauritania	66.7(63.8, 69.5)	59.7(57.4, 62.0)	-7.0(-10.6, -3.3)
Mexico	83.5(80.2, 86.9)	83.0(79.7, 86.3)	-0.5(-5.2, 4.2)
Moldova	87.3(83.8, 90.8)	82.9(78.5, 87.3)	-4.4(-10.0, 1.3)
Mongolia	78.3(75.8, 80.7)	76.4(73.7, 79.2)	-1.9(-5.6, 1.8)
Montenegro	95.7(92.6, 98.8)	95.7(93.1, 98.3)	0.0(-4.0, 4.1)
Nepal	83.6(79.4, 87.7)	63.0(60.2, 65.9)	-20.5(-25.6, -15.5)
Nigeria	76.1(73.9, 78.4)	56.8(55.5, 58.2)	-19.3(-21.9, -16.7)
Palestine	74.1(72.3, 76.0)	73.8(70.7, 76.8)	-0.3(-3.9, 3.2)
Panama	81.1(76.4, 85.8)	78.7(75.2, 82.3)	-2.4(-8.3, 3.5)

Paraguay	82.7(79.7, 85.7)	83.4(80.3, 86.6)	0.7(-3.6, 5.1)
Rwanda	78.2(75.0, 81.4)	71.1(69.5, 72.7)	-7.1(-10.7, -3.5)
Senegal	71.5(68.9, 74.1)	63.1(61.5, 64.6)	-8.4(-11.4, -5.4)
Serbia	97.8(96.8, 98.8)	94.4(91.7, 97.2)	-3.4(-6.3, -0.5)
Suriname	80.5(76.3, 84.7)	63.3(59.9, 66.6)	-17.3(-22.6, -11.9)
Swaziland	77.2(69.3, 85.1)	63.9(60.5, 67.4)	-13.3(-21.9, -4.6)
São Tomé and Principe	57.7(52.5, 63.0)	58.7(52.7, 64.7)	1.0(-7.0, 9.0)
Thailand	92.8(90.1, 95.5)	92.4(90.8, 94.1)	-0.3(-3.5, 2.8)
Timor-Leste	73.1(66.7, 79.6)	50.0(46.3, 53.6)	-23.2(-30.6, -15.7)
Togo	63.1(57.7, 68.5)	52.6(49.5, 55.7)	-10.5(-16.8, -4.3)
Trinidad and Tobago	90.8(86.8, 94.9)	94.5(91.4, 97.7)	3.7(-1.5, 8.8)
Tunisia	80.6(76.9, 84.2)	72.7(67.7, 77.6)	-7.9(-14.1, -1.7)
Turkmenistan	94.0(91.9, 96.0)	92.2(90.3, 94.2)	-1.8(-4.6, 1.1)
Uganda	80.6(78.3, 82.9)	63.0(61.8, 64.2)	-17.6(-20.2, -15.0)
Ukraine	91.4(89.1, 93.7)	90.0(87.3, 92.7)	-1.4(-5.0, 2.1)
Uruguay	87.7(81.4, 94.1)	76.9(56.5, 97.2)	-10.8(-32.2, 10.5)
Vietnam	91.9(89.0, 94.8)	90.0(87.6, 92.4)	-1.9(-5.6, 1.9)
Zimbabwe	66.2(63.0, 69.4)	63.2(61.2, 65.1)	-3.1(-6.8, 0.7)

*: Gaps by residential area are defined as the difference between rural and urban averages. A negative area gap implies urban advantage, a positive area gap rural advantage.

Table 22. Prevalence (%) of children on track according to the ECDI by household wealth quintile with 95% confidence intervals, the most recent years in 60 countries

Country	Wealth quintile (%)					Difference (richest - poorest)*
	Poorest	2	3	4	Richest	
Algeria	74.17	73.92	71.85	72.38	77.95	3.8(-1.0, 8.6)
Argentina	79.61	84.35	89.10	88.79	95.43	15.8(10.4, 21.3)
Bangladesh	58.36	60.81	65.77	63.77	74.49	16.1(12.3, 20.0)
Belarus	93.20	92.40	94.54	95.34	95.28	2.1(-2.6, 6.8)
Belize	78.19	79.86	83.13	88.32	94.95	16.8(9.9, 23.6)
Benin	58.52	61.77	62.23	61.87	74.05	15.5(10.5, 20.6)
Bhutan	68.95	69.30	71.88	71.03	78.35	9.4(2.6, 16.2)
Bosnia and Herzegovina	97.08	98.56	96.63	95.36	97.30	0.2(-2.9, 3.4)
Burundi	33.92	37.56	38.97	40.77	53.86	19.9(17.1, 22.8)
Cameroon	57.56	58.20	55.16	69.74	75.92	18.4(12.3, 24.4)
Central African Republic	45.44	44.56	48.50	53.83	50.42	5(-1.3, 11.2)
Chad	38.87	32.73	32.61	34.87	43.82	5(0.1, 9.8)
Congo, Rep.	45.61	47.94	53.65	64.40	70.70	25.1(19.3, 30.9)
Congo, Dem. Rep.	61.89	59.94	65.08	69.92	78.08	16.2(10.8, 21.5)
Costa Rica	70.76	76.66	83.84	93.77	87.41	16.6(5.0, 28.3)
Cote d'Ivoire	65.59	60.16	62.17	60.83	69.87	4.3(-1.7, 10.2)
Dominican Republic	78.44	86.12	88.32	90.11	91.42	13(9.0, 17.0)
El Salvador	82.73	78.26	80.68	84.50	87.77	5(0.1, 10.0)
Gambia	68.51	63.69	67.64	70.46	72.58	4.1(-1.4, 9.6)
Ghana	63.50	71.14	71.43	72.87	80.35	16.9(10.1, 23.6)
Guinea	44.91	50.55	46.78	51.47	52.72	7.8(1.8, 13.8)
Guinea-Bissau	69.42	63.85	63.81	56.04	62.70	-6.7(-13.1, -0.4)
Guyana	75.90	82.80	90.70	93.05	92.45	16.5(9.9, 23.2)
Jamaica	79.36	94.01	89.04	94.20	94.41	15.1(5.4, 24.7)
Jordan	57.91	62.27	62.77	76.46	73.62	15.7(9.8, 21.7)
Kazakhstan	83.16	88.75	87.49	88.10	89.66	6.5(-1.5, 14.5)
Kosovo	84.85	81.35	80.22	89.40	91.16	6.3(-1.7, 14.3)
Kyrgyzstan	78.86	84.94	79.51	79.36	85.77	6.9(0.1, 13.7)
Lebanon	83.46	85.00	86.01	88.00	89.51	6.1(-2.1, 14.2)
Macedonia	94.13	90.00	92.94	94.65	96.28	2.1(-3.9, 8.2)
Malawi	54.08	58.37	62.72	62.66	70.13	16(11.1, 21.0)
Maldives	88.55	90.27	94.71	93.97	94.58	6(1.0, 11.1)
Mali	56.73	63.06	65.12	62.64	68.50	11.8(7.3, 16.2)
Mauritania	54.74	58.89	64.98	66.45	69.71	15(9.4, 20.6)
Mexico	80.90	82.96	78.89	82.33	89.03	8.1(1.7, 14.6)
Moldova	81.78	82.92	88.18	86.36	86.44	4.7(-4.2, 13.5)
Mongolia	78.46	75.98	76.59	77.18	79.19	0.7(-5.0, 6.4)
Montenegro	97.29	92.49	96.96	91.30	99.74	2.5(-0.3, 5.3)
Nepal	55.74	67.37	57.60	61.97	80.70	25(17.9, 32.0)

Nigeria	48.68	49.56	58.52	70.74	86.88	38.2(35.3, 41.1)
Palestine	64.80	70.30	75.29	77.88	83.30	18.5(13.6, 23.3)
Panama	70.59	79.47	74.09	74.62	89.58	19(11.9, 26.0)
Paraguay	74.91	82.72	79.72	82.65	91.62	16.7(10.0, 23.4)
Rwanda	66.01	66.40	71.89	76.50	82.68	16.7(12.2, 21.2)
São Tomé and Principe	51.01	52.84	64.86	57.73	61.15	10.1(-2.5, 22.8)
Senegal	63.65	61.45	63.23	65.85	71.97	8.3(4.2, 12.4)
Serbia	93.63	93.72	97.51	98.23	98.79	5.2(1.1, 9.2)
Suriname	53.23	66.48	70.40	77.22	83.20	30(21.8, 38.1)
Swaziland	62.42	62.62	65.83	63.24	75.54	13.1(3.4, 22.8)
Thailand	87.47	90.84	95.04	90.98	98.00	10.5(5.7, 15.4)
Timor-Leste	41.65	46.28	53.29	63.62	76.39	34.7(25.3, 44.1)
Togo	48.05	48.81	54.33	59.32	65.21	17.2(9.0, 25.3)
Trinidad and Tobago	92.08	89.08	92.73	93.95	93.61	1.5(-6.9, 9.9)
Tunisia	67.46	76.58	78.83	75.09	85.73	18.3(8.7, 27.8)
Turkmenistan	88.27	92.53	97.04	96.17	92.43	4.2(-0.9, 9.2)
Uganda	55.36	60.50	62.65	69.26	82.28	26.9(23.6, 30.3)
Ukraine	91.04	89.67	89.05	93.33	91.83	0.8(-4.5, 6.0)
Uruguay	76.69	85.64	96.32	87.80	96.44	19.8(2.5, 37.0)
Vietnam	84.19	92.65	89.90	91.88	93.15	9(2.4, 15.6)
Zimbabwe	62.46	62.91	64.54	63.08	67.04	4.6(-0.5, 9.7)

*: Gaps by wealth quintile are defined as the difference between the richest and poorest quintile averages. A negative area gap implies poorest advantage. A positive gap richest advantage.

Table 23. Level and trends of national prevalence (%) of ECDI with 95% confidence intervals in 13 countries with data available in two rounds

Country	Round 1	Round 2	Round 1-Round 2*
Belize	88.6(86.1, 91.1)	84.9(82.3, 87.4)	3.7(0.1, 7.4)
Cameroon	52.0(49.8, 54.1)	62.4(60.3, 64.5)	-10.4(-13.4, -7.4)
Chad	34.1(32.6, 35.6)	36.4(34.9, 37.8)	-2.3(-4.4, -0.2)
Congo, Dem. Rep.	52.0(49.9, 54.1)	67.8(66.1, 69.6)	-15.8(-18.6, -13.1)
Kazakhstan	89.3(87.6, 91.0)	87.2(84.9, 89.6)	2.1(-0.8, 4.9)
Mauritania	62.0(60.2, 63.8)	62.7(60.9, 64.5)	-0.8(-3.3, 1.8)
Mongolia	89.4(87.5, 91.4)	77.5(75.6, 79.3)	12.0(9.3, 14.7)
Nigeria	62.5(61.1, 63.8)	62.7(61.5, 63.9)	-0.3(-2.1, 1.5)
Palestine	73.6(72.1, 75.0)	74.0(72.4, 75.6)	-0.4(-2.6, 1.7)
Serbia	95.3(93.6, 97.1)	96.7(95.5, 97.8)	-1.3(-3.4, 0.8)
Swaziland	63.1(60.1, 66.2)	66.7(63.4, 69.9)	-3.5(-8.0, 0.9)
Thailand	94.4(93.2, 95.5)	92.6(91.1, 94.1)	1.8(-0.1, 3.6)
Vietnam	85.3(83.2, 87.4)	90.6(88.7, 92.5)	-5.3(-8.1, -2.4)

*: A negative sign implies an increase in the % of children on track according to the ECDI, a positive sign a reduction in the % of children on track according to the ECDI.

Table 24. Level and trends of gender inequalities in the prevalence (%) of ECDI with 95% confidence intervals in 13 countries with data available in two rounds

Country	Round 1			Round 2			Round1 – Round2
	Male	Female	Gap	Male	Female	Gap	
Belize	88.7	88.5	0.1(-4.9, 5.2)	81.7	88.2	-6.5(-11.6, -1.4)	6.4(-0.4, 13.6)
Cameroon	50.8	53.1	-2.3(-6.5, 2.0)	60.2	64.6	-4.4(-8.6, -0.2)	2.2(-3.8, 8.1)
Chad	32.9	35.2	-2.2(-5.2, 0.7)	34.4	38.4	-4.0(-7.0, -1.1)	1.8(-2.4, 6.0)
Congo, Dem. Rep.	50.6	53.4	-2.8(-6.9, 1.3)	66.9	68.7	-1.8(-5.3, 1.7)	-0.9(-6.4, 4.5)
Kazakhstan	86.8	92.1	-5.3(-8.6, -2.0)	85.8	88.7	-2.9(-7.6, 1.8)	-2.4(-8.1, 3.3)
Mauritania	61.6	62.3	-0.7(-4.3, 2.9)	61	64.3	-3.3(-6.9, 0.3)	2.6(-2.6, 7.7)
Mongolia	85.9	92.8	-6.8(-10.7, -2.9)	74.7	80.3	-5.7(-9.3, -2.0)	-1.2(-6.5, 4.2)
Nigeria	61.2	63.8	-2.5(-5.3, 0.2)	61.6	63.8	-2.3(-4.6, 0.1)	-0.3(-3.9, 3.3)
Palestine	72.1	75.2	-3.2(-6, -0.3)	69.4	79	-9.6(-12.7, -6.4)	6.4(2.2, 10.7)
Serbia	94.4	96.2	-1.8(-5.3, 1.7)	96.5	96.9	-0.4(-2.7, 1.9)	-1.4(-5.6, 2.8)
Swaziland	61.8	64.4	-2.6(-8.7, 3.5)	64.8	68.6	-3.8(-10.2, 2.6)	1.2(-7.7, 10.1)
Thailand	92.9	95.8	-2.9(-5.1, -0.7)	91.4	93.8	-2.3(-5.3, 0.6)	-0.6(-4.3, 3.1)
Vietnam	85.8	84.8	1.0(-3.3, 5.3)	89.9	91.3	-1.4(-5.2, 2.4)	0.4(-1.3, 2.1)

*: Gender gaps are defined as the difference between boy and girl averages. A negative sign implies a reduction of inequality, a positive sign implies an increase of inequality.

Lu C, *et al. BMJ Global Health* 2020; 5:e002314. doi: 10.1136/bmjgh-2020-002314

Table 25. Level and trends of area inequalities in the prevalence (%) of ECDI with 95% confidence intervals in 13 countries with data available in two rounds

Country	Round 1			Round 2			Round1 – Round2
	Rural	Urban	Gap	Rural	Urban	Gap	
Belize	86.4	92.1	-5.7(-10.7, -0.8)	82.6	88.5	-5.9(-10.9, -0.8)	0.1(-6.9, 7.2)
Cameroon	43.9	63.8	-19.9(-24.1, -15.7)	57.1	70.2	-13.1(-17.2, -9.0)	-6.8(-12.7, -1.0)
Chad	32.0	42.3	-10.2(-13.9, -6.6)	34.4	46.0	-11.6(-15.6, -7.6)	1.4(-4.0, 6.8)
Congo Dem. Rep.	48.0	62.9	-14.9(-18.9, -10.9)	65.6	73.0	-7.4(-11.0, -3.8)	-7.6(-13.0, -2.2)
Kazakhstan	87.6	91.2	-3.5(-6.9, -0.2)	86.9	87.6	-0.7(-5.3, 3.9)	-2.8(-8.5, 2.9)
Mauritania	59.2	67.1	-7.9(-11.7, -4.0)	59.7	66.7	-7.0(-10.6, -3.3)	-0.9(-6.2, 4.4)
Mongolia	89.3	89.5	-0.2(-4.0, 3.5)	76.4	78.3	-1.9(-5.6, 1.8)	1.6(-3.7, 6.9)
Nigeria	57.0	75.4	-18.4(-21.8, -15.1)	56.8	76.1	-19.3(-21.9, -16.7)	0.8(-3.4, 5.1)
Palestine	74.2	73.3	0.9(-2.3, 4.0)	73.8	74.1	-0.3(-3.9, 3.2)	-0.6 (-1.3, 0.2)
Serbia	95.2	95.5	-0.3(-3.9, 3.2)	94.4	97.8	-3.4(-6.3, -0.5)	3.1(-1.5, 7.6)
Swaziland	60.3	75.7	-15.4(-22.1, -8.7)	63.9	77.2	-13.3(-21.9, -4.6)	-2.1(-13.0, 8.8)
Thailand	94.3	94.4	-0.1(-2.3, 2.1)	92.4	92.8	-0.3(-3.5, 2.8)	0.2(-3.6, 4.1)
Vietnam	83.4	90.3	-6.9(-10.8, -2.9)	90.0	91.9	-1.9(-5.6, 1.9)	-5.0(-10.5, 0.4)

*: Gaps by residential area are defined as the difference between rural and urban averages. A negative sign implies a reduction of inequality, a positive sign implies an increase of inequality.

Table 26. Level and trends of wealth inequalities in the prevalence (%) of ECDI with 95% confidence intervals in 13 countries with data available in two rounds

Country	Round 1			Round 2			Round1 – Round2
	Q5	Q1	Gap	Q5	Q1	Gap	
Belize	93.3	81.6	11.8(3.5, 20.0)	95.0	78.2	16.8(10.0, 23.6)	5.0(-5.7, 15.7)
Cameroon	67.8	38.0	29.8(23.4, 36.1)	75.9	57.6	18.4(12.3, 24.4)	-11.4(-20.2, -2.7)
Chad	43.1	29.0	14.1(9.4, 18.9)	43.8	38.9	5.0(0.1, 9.8)	-9.2(-16.0, -2.4)
Congo, Dem. Rep.	70.3	44.0	26.3(20.5, 32.1)	78.1	61.9	16.2(10.8, 21.5)	-10.1(-18.0, -2.2)
Kazakhstan	95.0	86.6	8.4(3.6, 13.3)	89.7	83.2	6.5(-1.5, 14.5)	-1.9(-11.3, 7.4)
Mauritania	72.6	50.3	22.3(16.7, 27.8)	69.7	54.7	15.0(9.4, 20.6)	-7.3(-15.2, 0.5)
Mongolia	88.1	87.7	0.4(-5.9, 6.7)	79.2	78.5	0.7(-4.9, 6.4)	0.4(-8.1, 8.9)
Nigeria	80.4	48.9	31.5(27.5, 35.4)	86.9	48.7	38.2(35.3, 41.1)	6.8(1.9, 11.6)
Palestine	80.5	65.8	14.6(10.1, 19.1)	83.3	64.8	18.5(13.7, 23.3)	3.9(-2.7, 10.5)
Serbia	96.8	91.6	5.3(-1.1, 11.6)	98.8	93.6	5.2(1.1, 9.2)	-0.1(-7.7, 7.4)
Swaziland	78.2	51.1	27.1(18.1, 36.0)	75.5	62.4	13.1(3.4, 22.8)	-14.0(-27.1, -0.8)
Thailand	96.7	92.1	4.6(1.3, 7.9)	98.0	87.5	10.5(5.7, 15.4)	5.9(0.1, 11.8)
Vietnam	91.7	80.6	11.1(4.5, 17.7)	93.2	84.2	9.0(2.4, 15.5)	-2.1(-11.5, 7.2)

*: Gaps by wealth quintile are defined as the difference between the richest and poorest quintile averages. A negative sign implies a reduction of inequality, a positive sign implies an increase of inequality.
